# Author Correction: Embryological cellular origins and hypoxia-mediated mechanisms in *PIK3CA*-Driven refractory vascular malformations

**DOI:** 10.1038/s44321-025-00246-y

**Published:** 2025-05-07

**Authors:** Sota Torii, Keiki Nagaharu, Nanako Nakanishi, Hidehito Usui, Yumiko Hori, Katsutoshi Hirose, Satoru Toyosawa, Eiichi Morii, Mitsunaga Narushima, Yoshiaki Kubota, Osamu Nakagawa, Kyoko Imanaka-Yoshida, Kazuaki Maruyama

**Affiliations:** 1 Department of Pathology and Matrix Biology, Graduate School of Medicine, Mie University, 2-174 Edobashi, Tsu, Mie 514-8507 Japan; 2 Department of Hematology and Oncology, Mie University Graduate School of Medicine, 2-174 Edobashi, Tsu, 514-8507 Japan; 3 Department of Surgery, Kanagawa Children’s Medical Center, 2-138-4 Mutsukawa, Minami-ku, Yokohama, Kanagawa Japan; 4 Department of Pathology, Osaka University Graduate School of Medicine, 2-2 Yamadaoka, Suita, Osaka, 565-0871 Japan; 5 Department of Central Laboratory and Surgical Pathology, NHO Osaka National Hospital, 2-1-14 Hoenzaka, Chuo-ku, Osaka, 540-0006 Japan; 6 Department of Oral and Maxillofacial Pathology, Osaka University Graduate School of Dentistry, 1-8 Yamadaoka, Suita, Osaka, 565-0871 Japan; 7 Department of Plastic and Reconstructive Surgery, Mie University Graduate School of Medicine, 2-174 Edobashi, Tsu, Mie 514-8507 Japan; 8 Department of Anatomy, Keio University School of Medicine, 35 Shinanomachi, Shinjuku-ku, Tokyo, 160-8582 Japan; 9 Department of Molecular Physiology, National Cerebral and Cardiovascular Center Research Institute, 6-1 Kishibe-shimmachi, Suita, Osaka, 564-8565 Japan

## Abstract

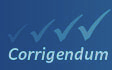

**Correction to:** *EMBO Molecular Medicine* (2025). 10.1038/s44321-025-00235-1 | Published online 16 April 2025

The Synopsis bullet point list is corrected.

From:

- Phosphatase DUSP22 was identified as a regulator of skeletal muscle atrophy and treatment with the small molecule BML-260 produced therapeutic effects in multiple models of muscle wasting.
- DUSP22 gene knockdown or BML-260 treatment suppressed activation of the stress kinase JNK and its downstream target FOXO3a, which is a master regulator of muscle wasting.
- In aged skeletal muscle, DUSP22 gene knockdown reduced wasting-related gene expression by >50% and BML-260 therapy increased grip strength by >20%.
- These results demonstrate that the DUSP22-JNK-FOXO3a axis may be utilized to treat sarcopenia or related muscle wasting disorders and BML-260 can be an attractive lead compound for further drug development.

To:

- A mouse model was developed to mimic PIK3CAH1047R-driven vascular malformations restricted to the head and neck.
- Single-cell RNA-seq revealed significant upregulation of hypoxia pathways, including HIF-1α stabilization and VEGF-A expression.
- HIF-1α and VEGF-A inhibition reduced abnormal vessel growth, suggesting a potential therapeutic approach for refractory lesions.

