# Supplementary material for: Embryological cellular origins and hypoxia-mediated mechanisms in PIK3CA-driven refractory vascular malformations
Source: EMBO Mol Med. 2025 Apr 16;17(6):1289–324. doi: 10.1038/s44321-025-00235-1 (PMC12162881; doi:10.1038/s44321-025-00235-1)
Supplement: Supplementary file 1 — Table EV1 [file 44321_2025_235_MOESM1_ESM.docx]

|  | Forward(5'-3') | Reverse(5'-3') |
| --- | --- | --- |
| R26R-eYFP KI | AAAGTCGCTCTGAGTTGTTAT | AAGACCGCGAAGACTTTGTC |
| R26R-eYFP WT | AAAGTCGCTCTGAGTTGTTAT | GGAGCGGGAGAAATGGATATG |
| R26R-tdTomato KI | GGCATTAAAGCATATCC | CTGTTCCTGTACGGCATGG |
| R26R-tdTomato KI | AAGGGAGCTGCAGTGGAGTA | CCGAAAATCTGTGGGAAGTC |
| Cre | ACATGTTCAGGGATCGCCAG | TAACCAGTGAAACAGCATTGC |
| PIK3CA^H1047R^  Mutant | CTGGCTTCTGAGGACCG | CGAAGAGTTTGTCCTCAACCG |
| PIK3CA^H1047R^  WT | CTGGCTTCTGAGGACCG | AATCTGTGGGAAGTCTTGTCC |
| Isl1-CreERT2 Mutant | GCCACTATTTGCCACCTAGC | CGGTTCAGCATCCAACAAG |
| Isl1-CreERT2  WT | GCCACTATTTGCCACCTAGC | CGGTTCAGCATCCAACAAG |
| Myf5-CreERT2  Mutant | GCTTCCAATTGCTTTAGATAC ATGA | GCTTCCAATTGCTTTAGATACATG A |
| Myf5-CreERT2  WT | GCTTCCAATTGCTTTAGATAC ATGA | CCTGTAATGGATTCCAAGCTG |
| Pax3-CreERT2  Mutant | GAGCCTGTGGACTTGGATCT | TCACCCTGAAGTTCTCAGGATC |
| Pax3-CreERT2  WT | GAGCCTGTGGACTTGGATCT | GCTGCGTGGGTAATTCTG |

Table EV1: Primers used for genotyping
