# Supplementary material for: Embryological cellular origins and hypoxia-mediated mechanisms in PIK3CA-driven refractory vascular malformations
Source: EMBO Mol Med. 2025 Apr 16;17(6):1289–324. doi: 10.1038/s44321-025-00235-1 (PMC12162881; doi:10.1038/s44321-025-00235-1)
Supplement: Supplementary file 2 — Table EV2 [file 44321_2025_235_MOESM2_ESM.docx]

| Age | Sex | Location of the lesion | Variant Allele Frequency (VAF) | Ethnicity | Corresponding Figures |
| --- | --- | --- | --- | --- | --- |
| 64 | M | Heart | Not Available (control) | Japanese (asian) | Figure 5A-C’’’” |
| 12 | F | Body skin | 11.2% | Japanese (asian) | Figure 5D-K |
| 27 | M | Left upper limb | 9.4% | Japanese (asian) | Figure 5L-S |
| 69 | M | Mesentery | 7.0% | Japanese (asian) | Figure EV 8A-H |
| 2 | F | Buttock | 7.7% | Japanese (asian) | Figure EV 8I-P |
| 3 | M | Scrotum | 2.8% | Japanese (asian) | Figure EV 8Q-X |

Table EV2. Brief clinical information for human vascular malformation
