# Supplementary figures and images for: Embryological cellular origins and hypoxia-mediated mechanisms in PIK3CA-driven refractory vascular malformations
Source: EMBO Mol Med. 2025 Apr 16;17(6):1289–324. doi: 10.1038/s44321-025-00235-1 (PMC12162881; doi:10.1038/s44321-025-00235-1)

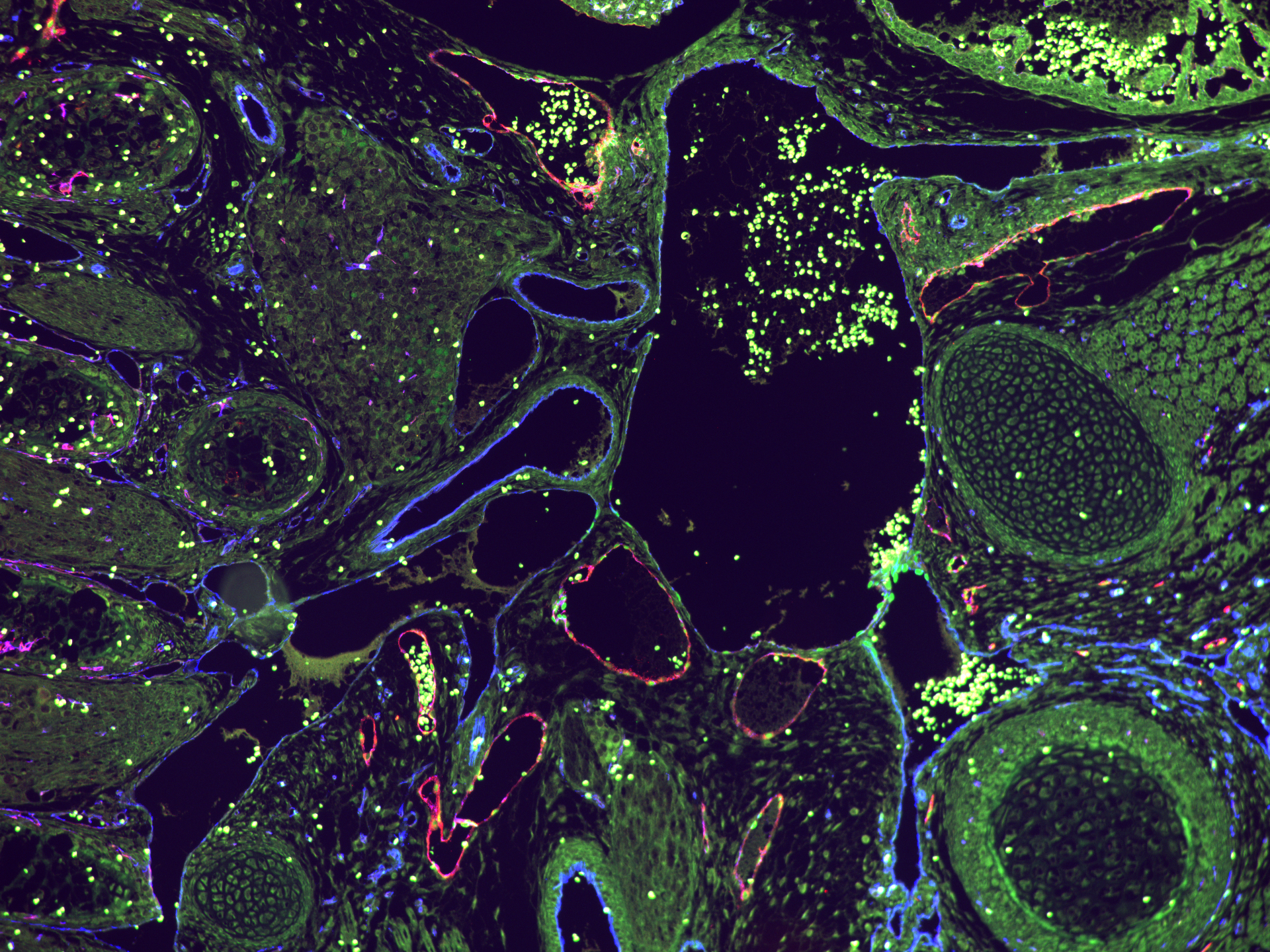

Supplement: Supplementary file 8 — Source data Fig. 2 [file 44321_2025_235_MOESM8_ESM.zip › Figure 2/Figure 2E.tif]

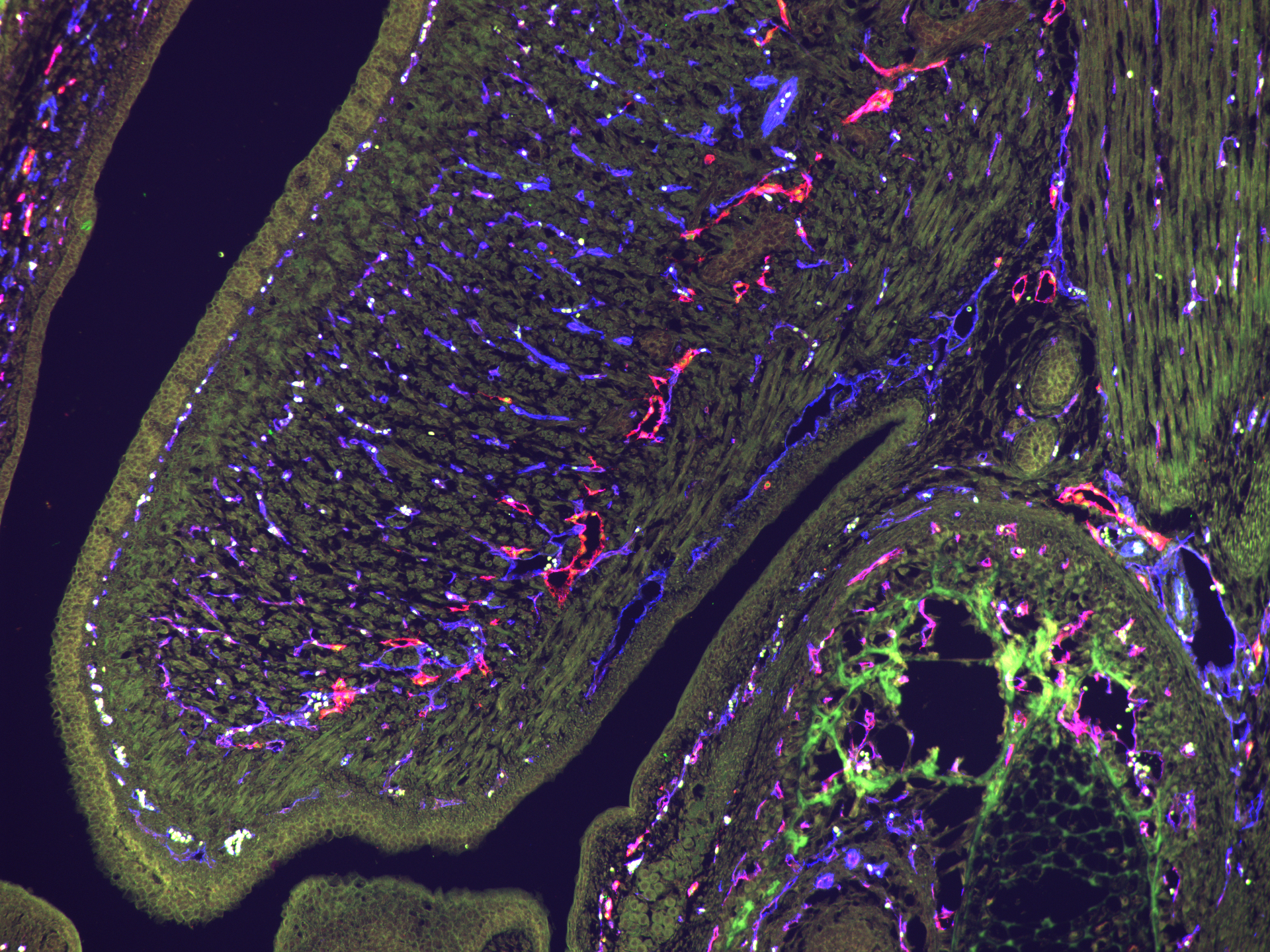

Supplement: Supplementary file 8 — Source data Fig. 2 [file 44321_2025_235_MOESM8_ESM.zip › Figure 2/Figure 2R.tif]

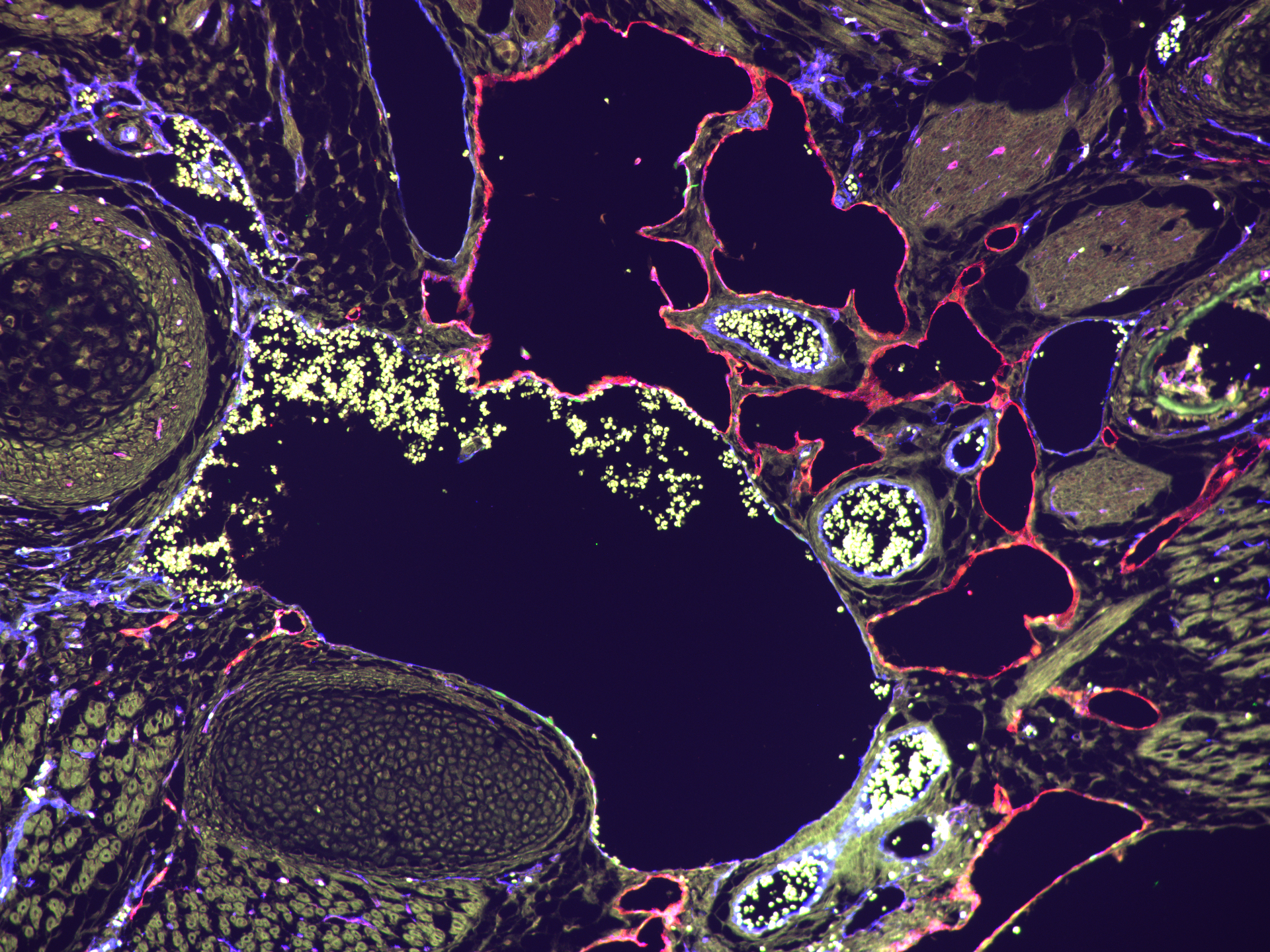

Supplement: Supplementary file 8 — Source data Fig. 2 [file 44321_2025_235_MOESM8_ESM.zip › Figure 2/Figure 2S.tif]

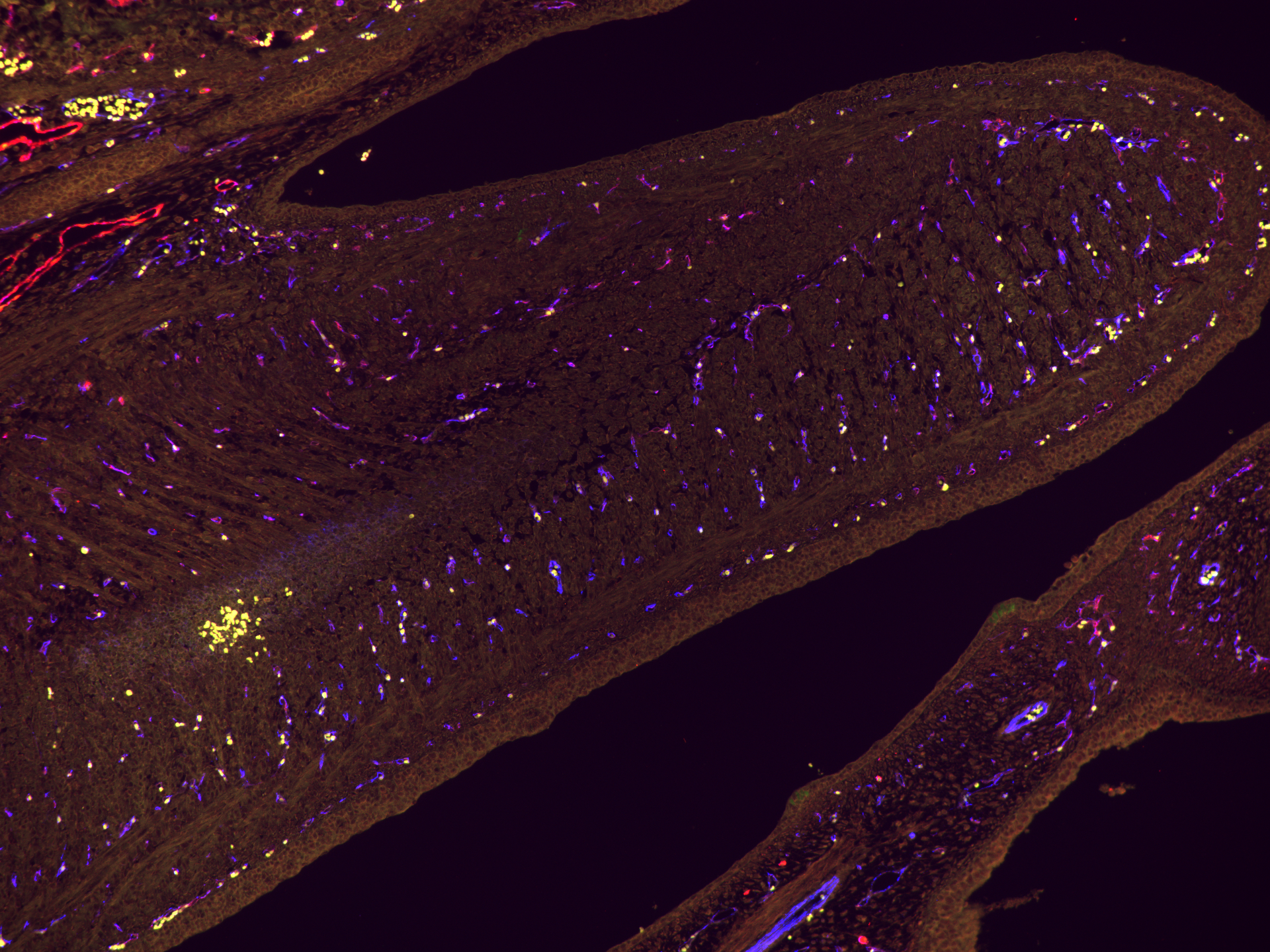

Supplement: Supplementary file 8 — Source data Fig. 2 [file 44321_2025_235_MOESM8_ESM.zip › Figure 2/Figure 2D.tif]

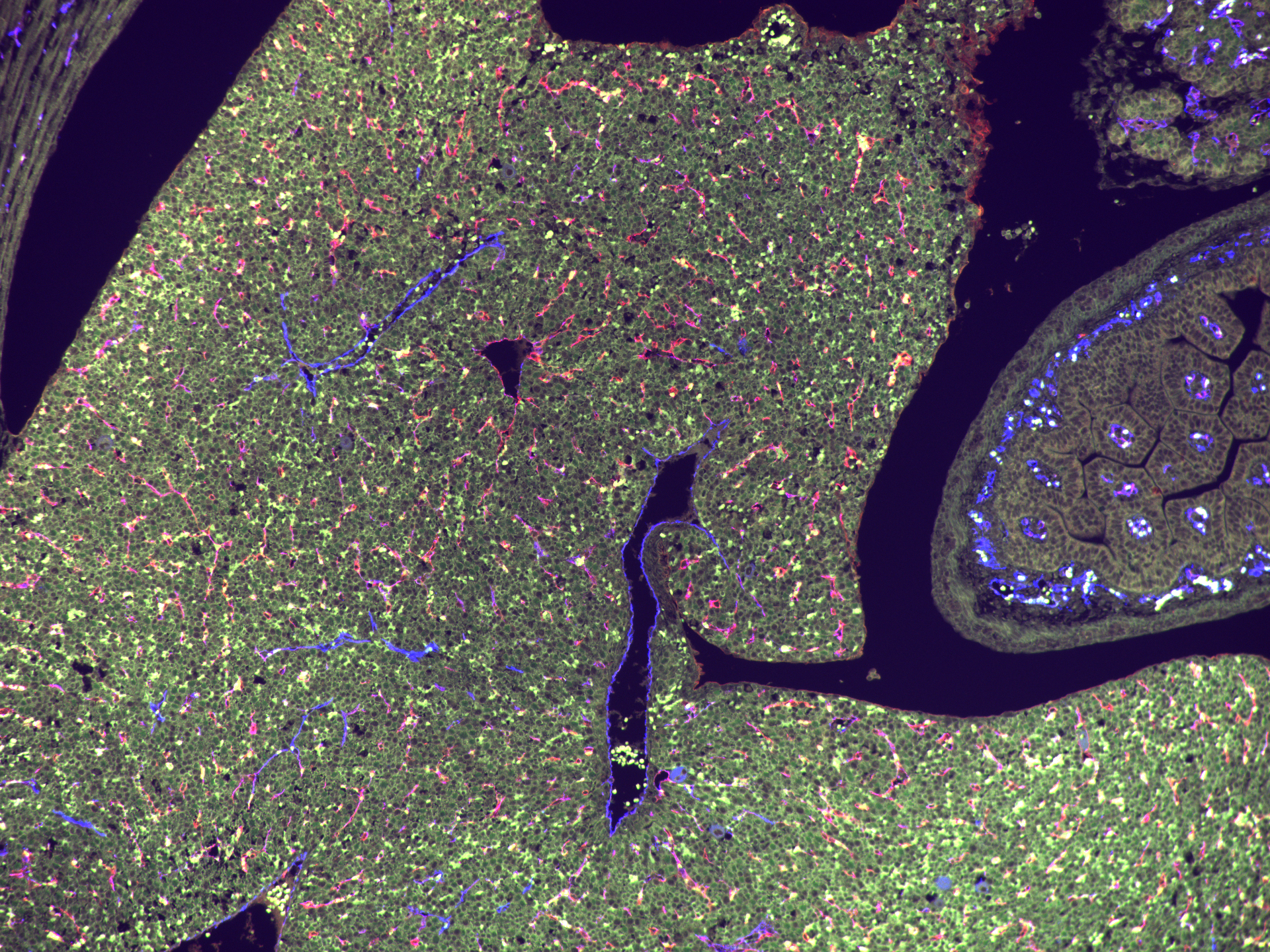

Supplement: Supplementary file 8 — Source data Fig. 2 [file 44321_2025_235_MOESM8_ESM.zip › Figure 2/Figure 2F.tif]

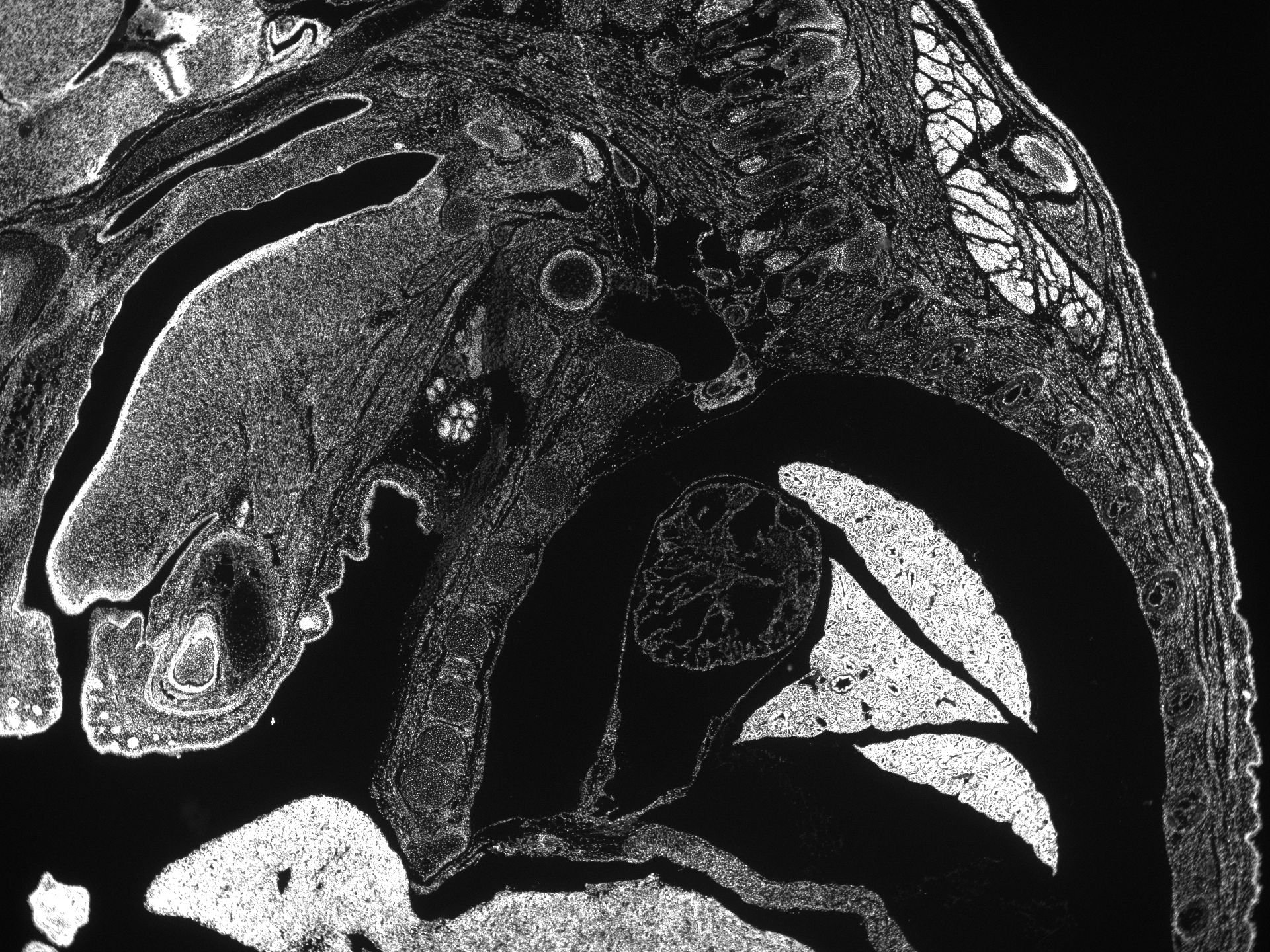

Supplement: Supplementary file 8 — Source data Fig. 2 [file 44321_2025_235_MOESM8_ESM.zip › Figure 2/figure 2Q.tif]

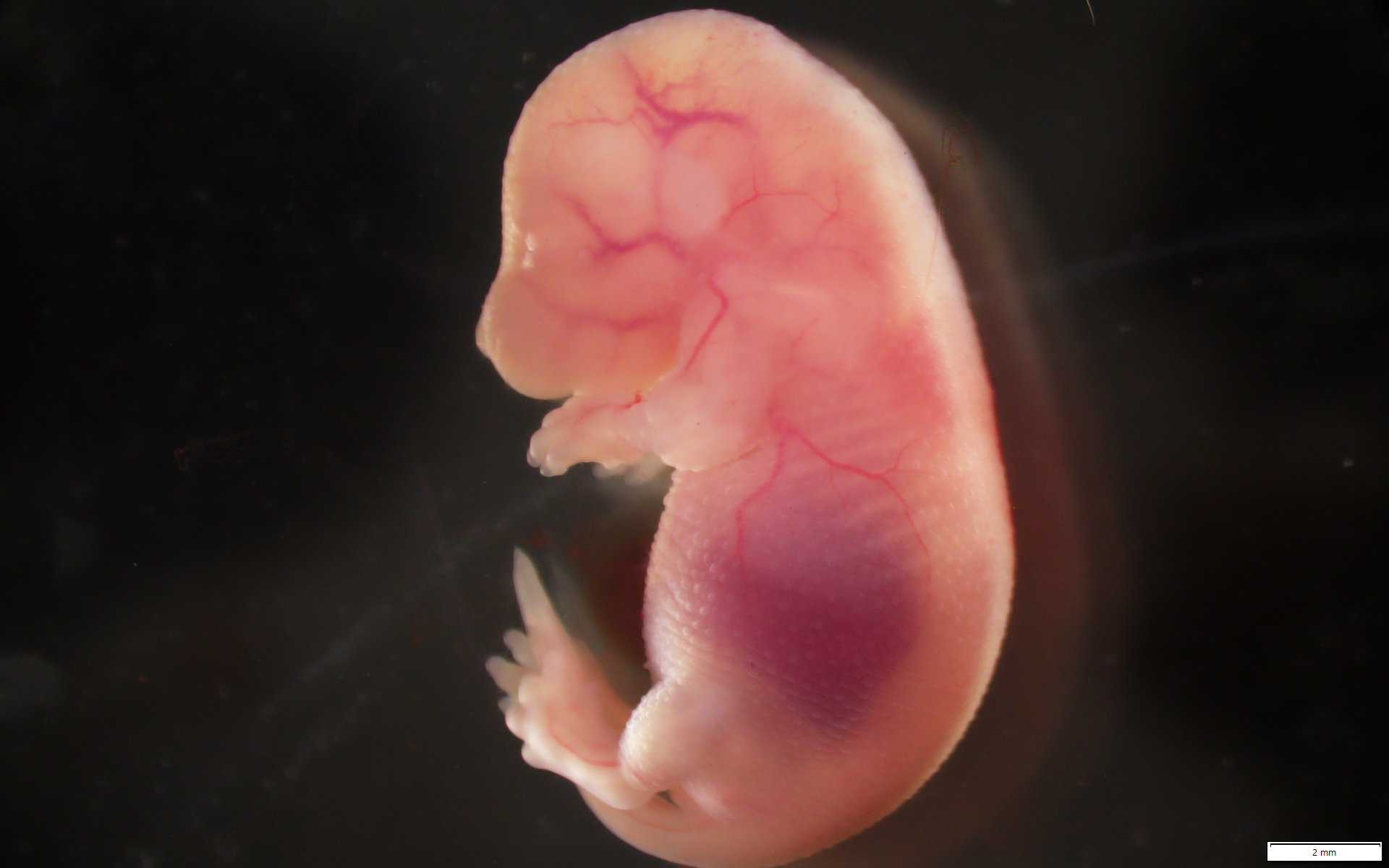

Supplement: Supplementary file 8 — Source data Fig. 2 [file 44321_2025_235_MOESM8_ESM.zip › Figure 2/Figure 2P.tif]

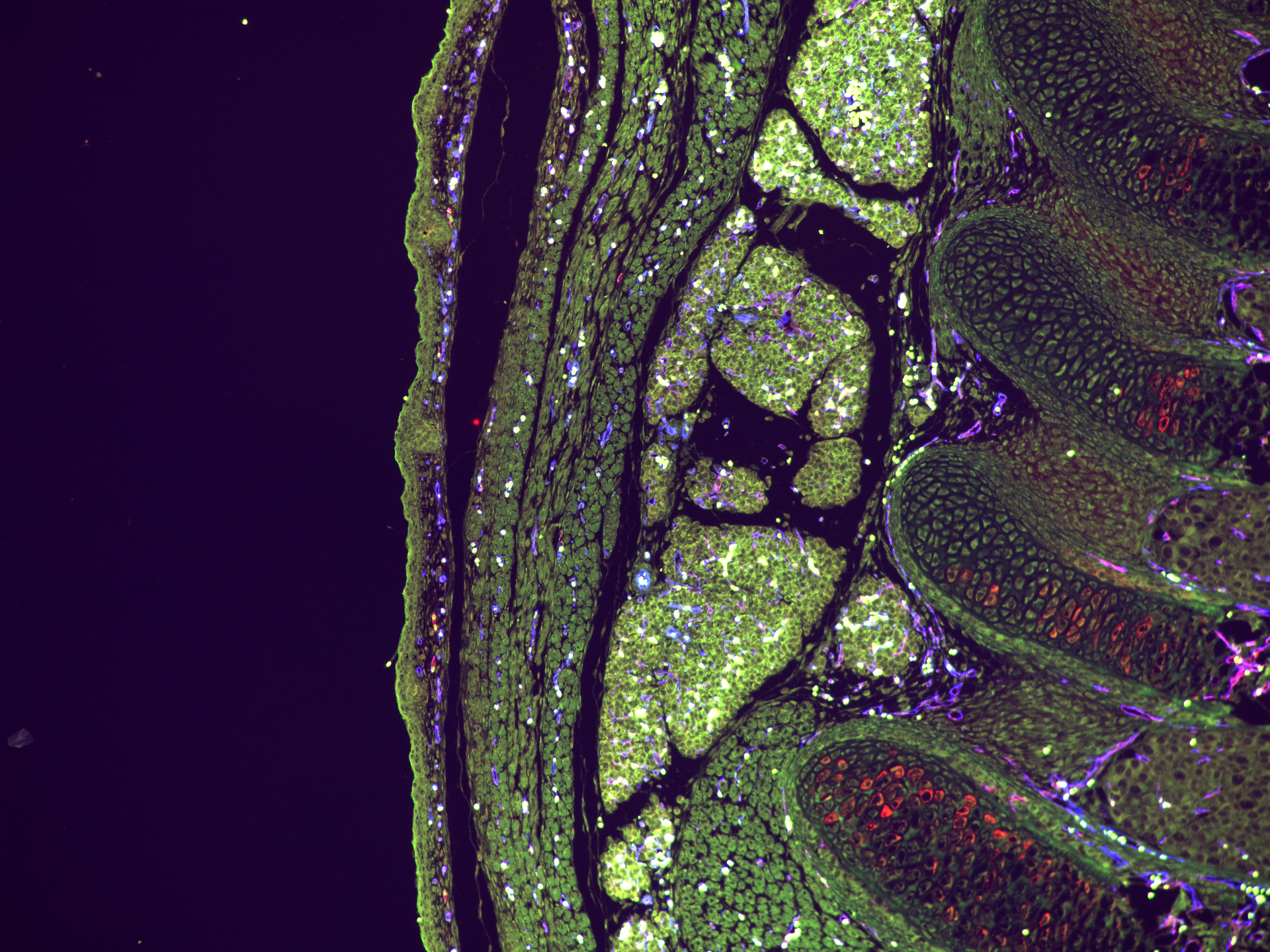

Supplement: Supplementary file 8 — Source data Fig. 2 [file 44321_2025_235_MOESM8_ESM.zip › Figure 2/Figure 2G.tif]

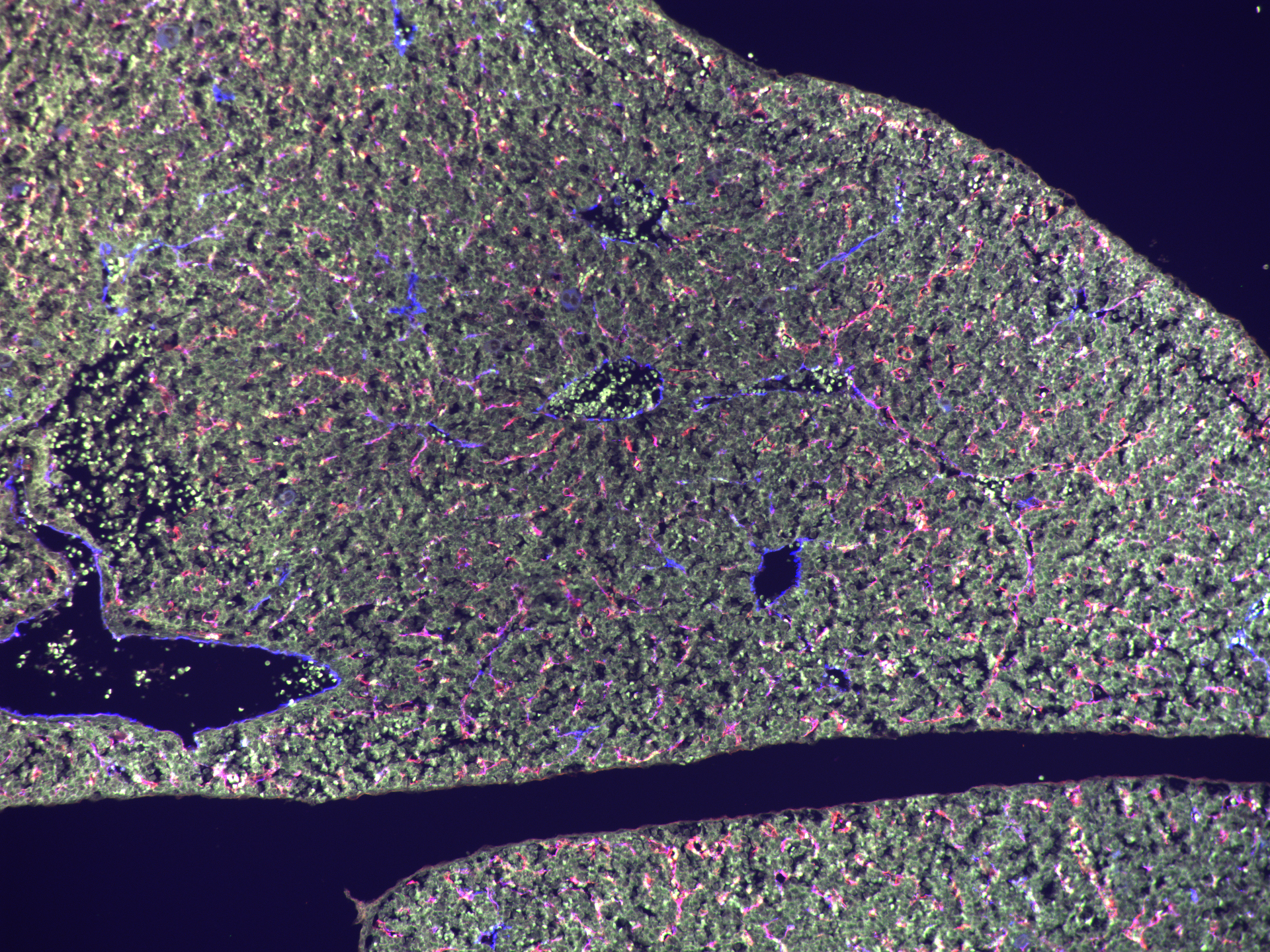

Supplement: Supplementary file 8 — Source data Fig. 2 [file 44321_2025_235_MOESM8_ESM.zip › Figure 2/Figure 2T.tif]

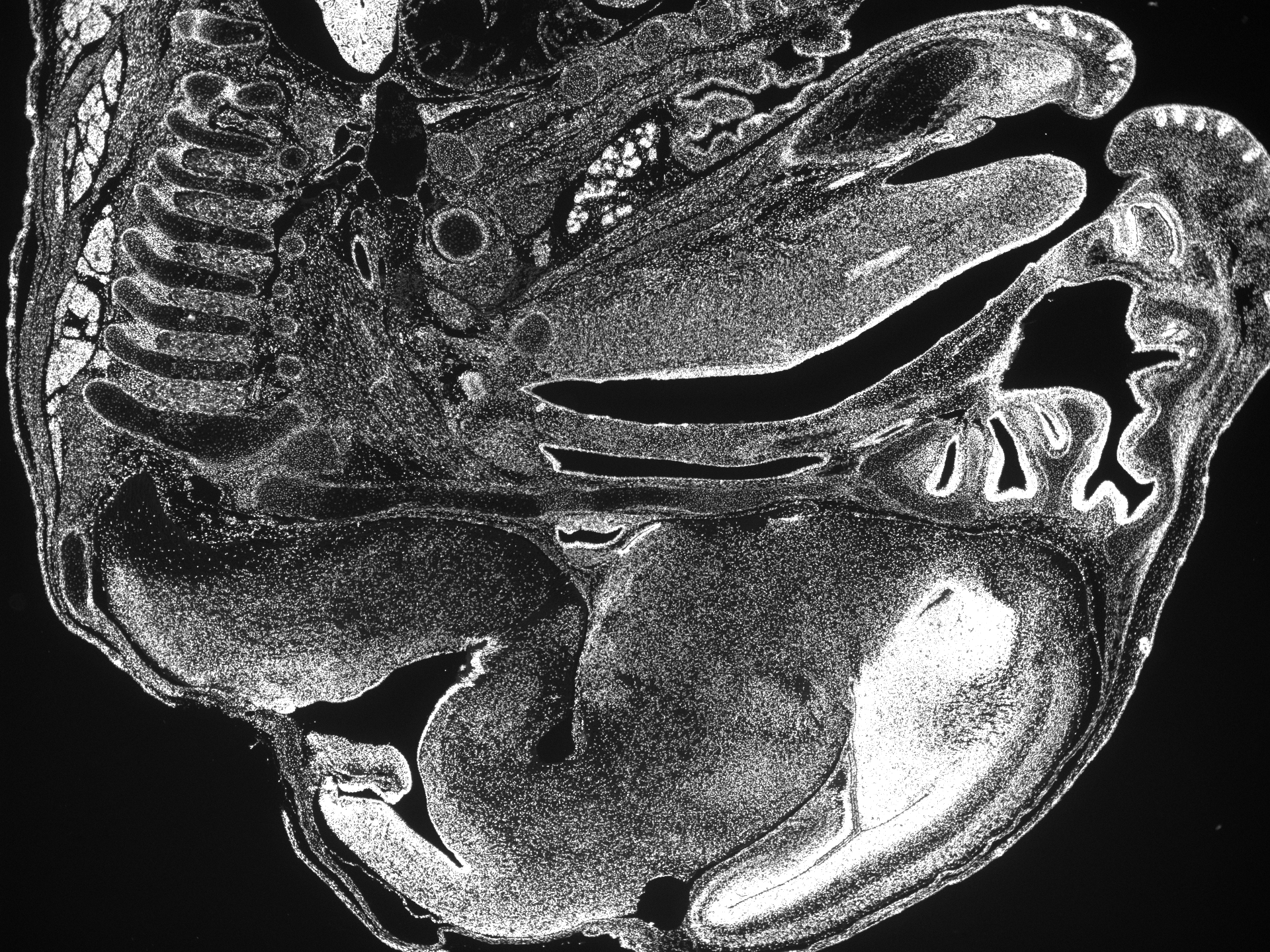

Supplement: Supplementary file 8 — Source data Fig. 2 [file 44321_2025_235_MOESM8_ESM.zip › Figure 2/Figure 2C.tif]

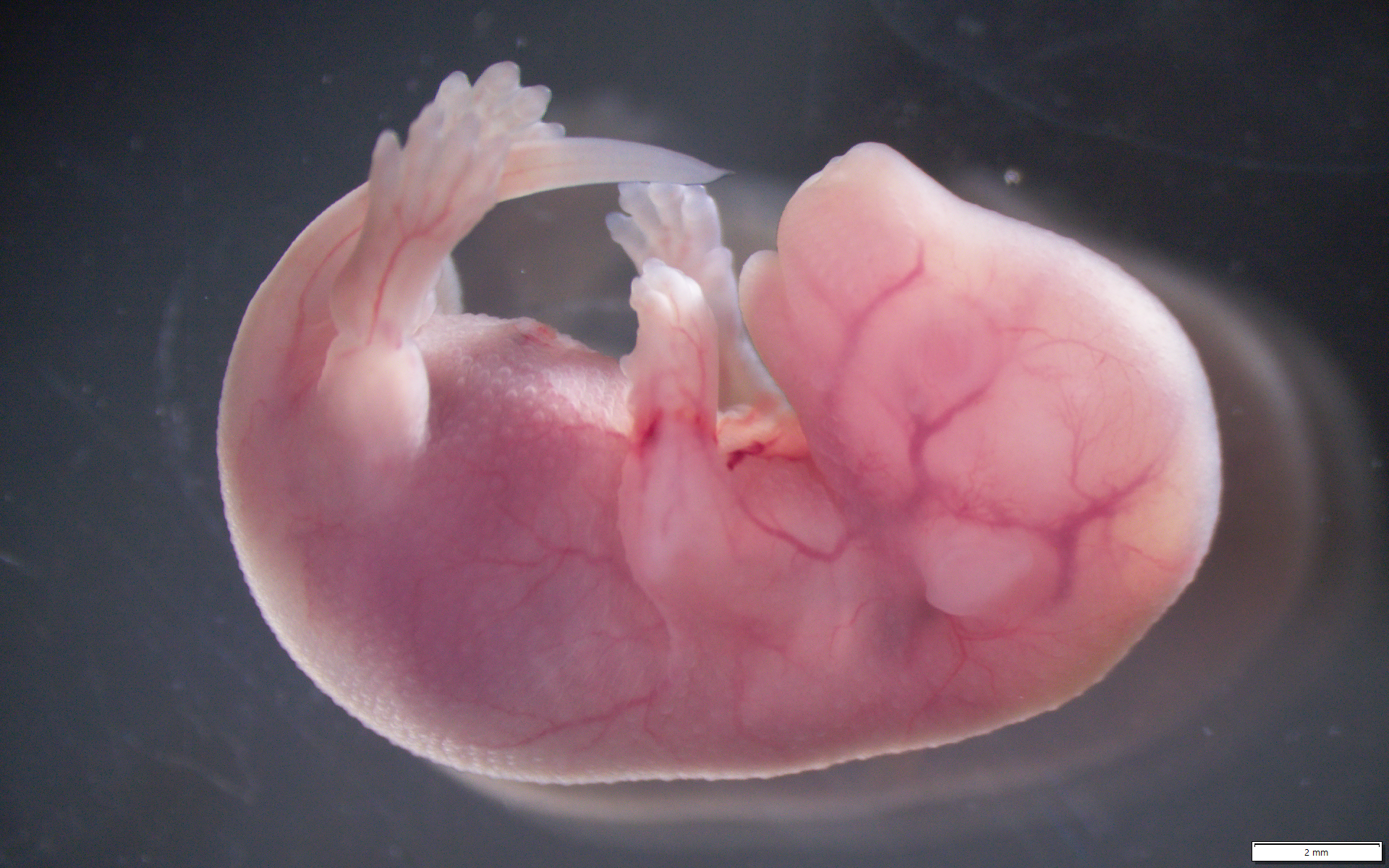

Supplement: Supplementary file 8 — Source data Fig. 2 [file 44321_2025_235_MOESM8_ESM.zip › Figure 2/Figure 2B.tif]

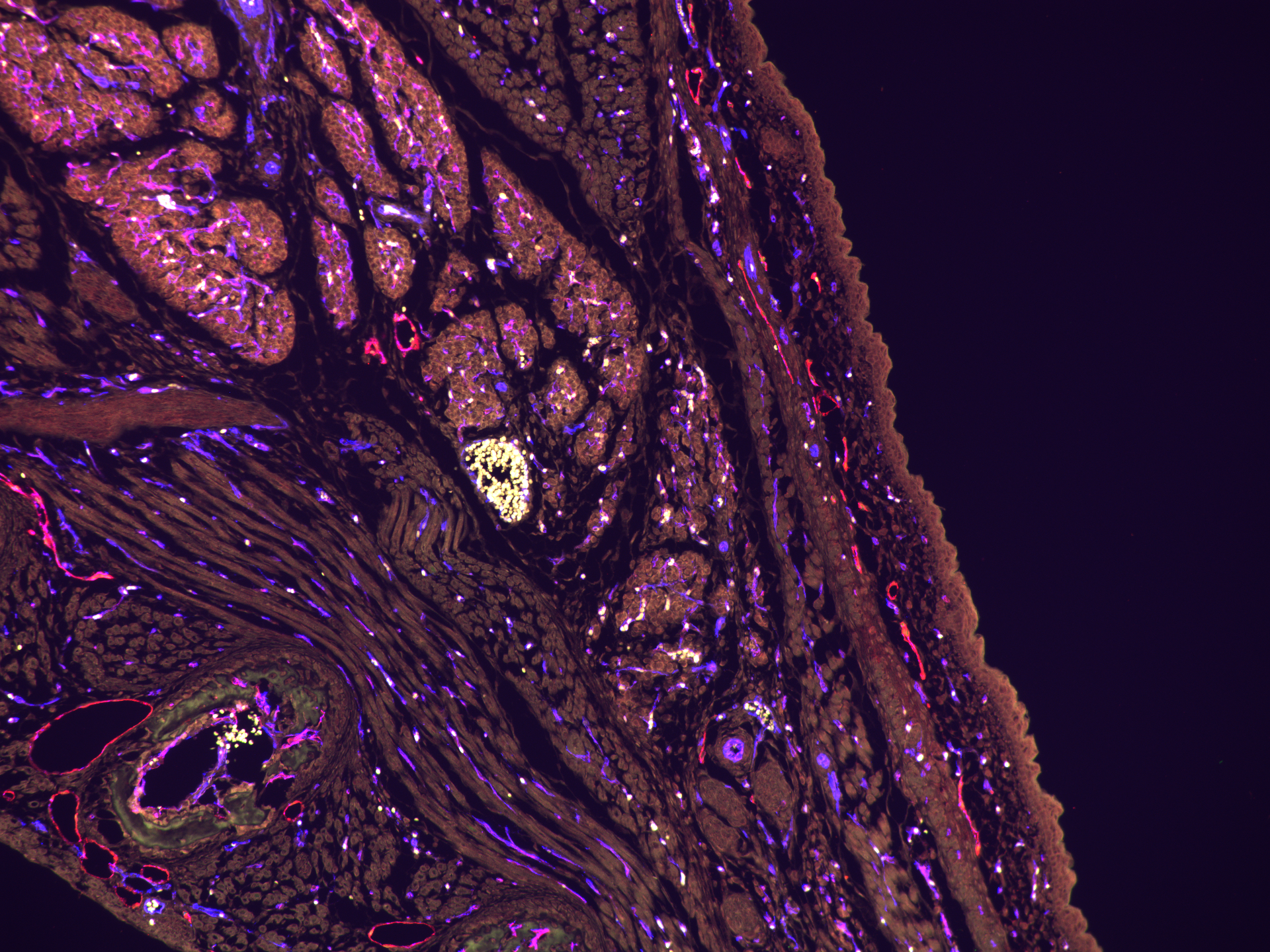

Supplement: Supplementary file 8 — Source data Fig. 2 [file 44321_2025_235_MOESM8_ESM.zip › Figure 2/Figure 2U.tif]

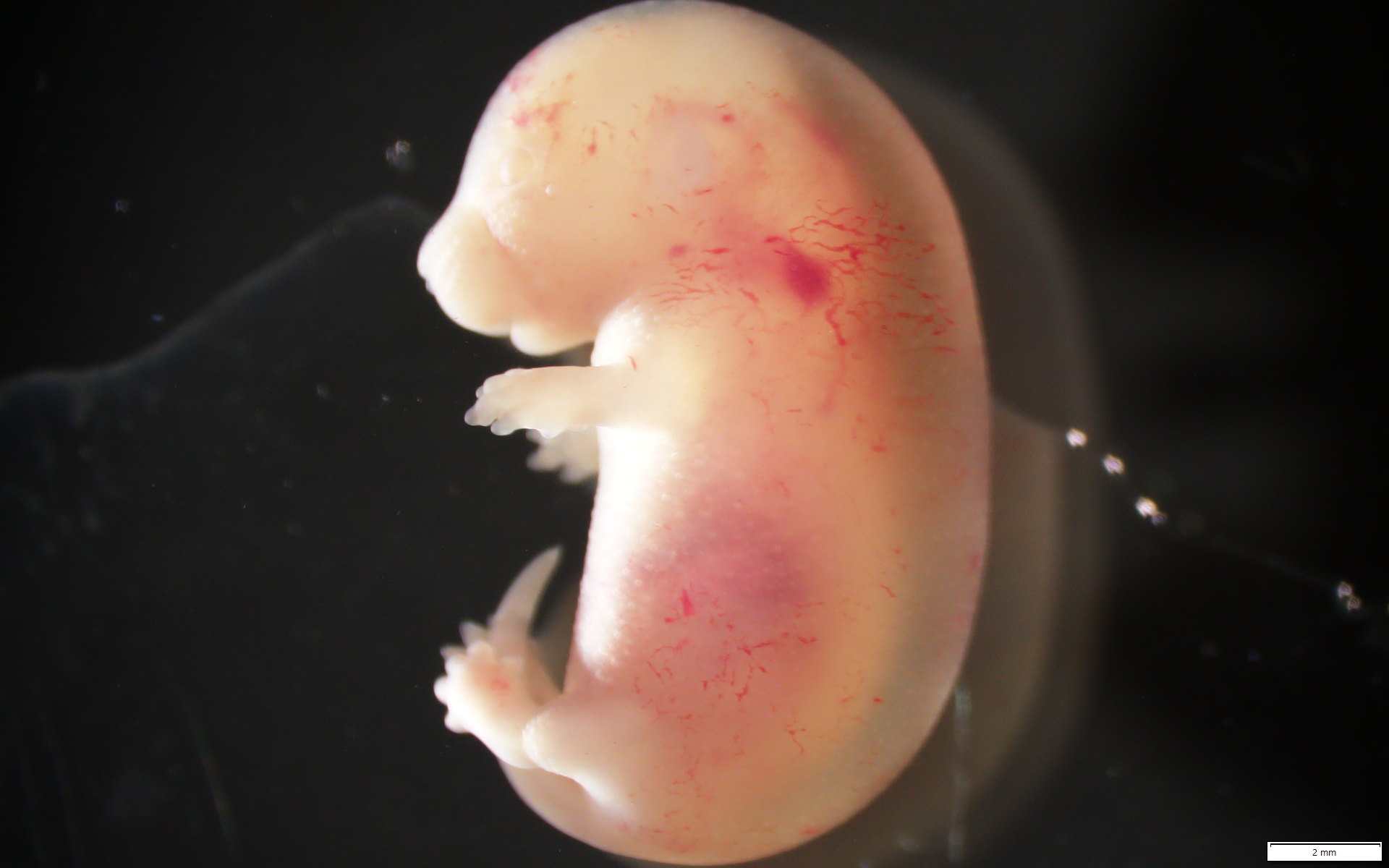

Supplement: Supplementary file 8 — Source data Fig. 2 [file 44321_2025_235_MOESM8_ESM.zip › Figure 2/Figure 2W.tif]

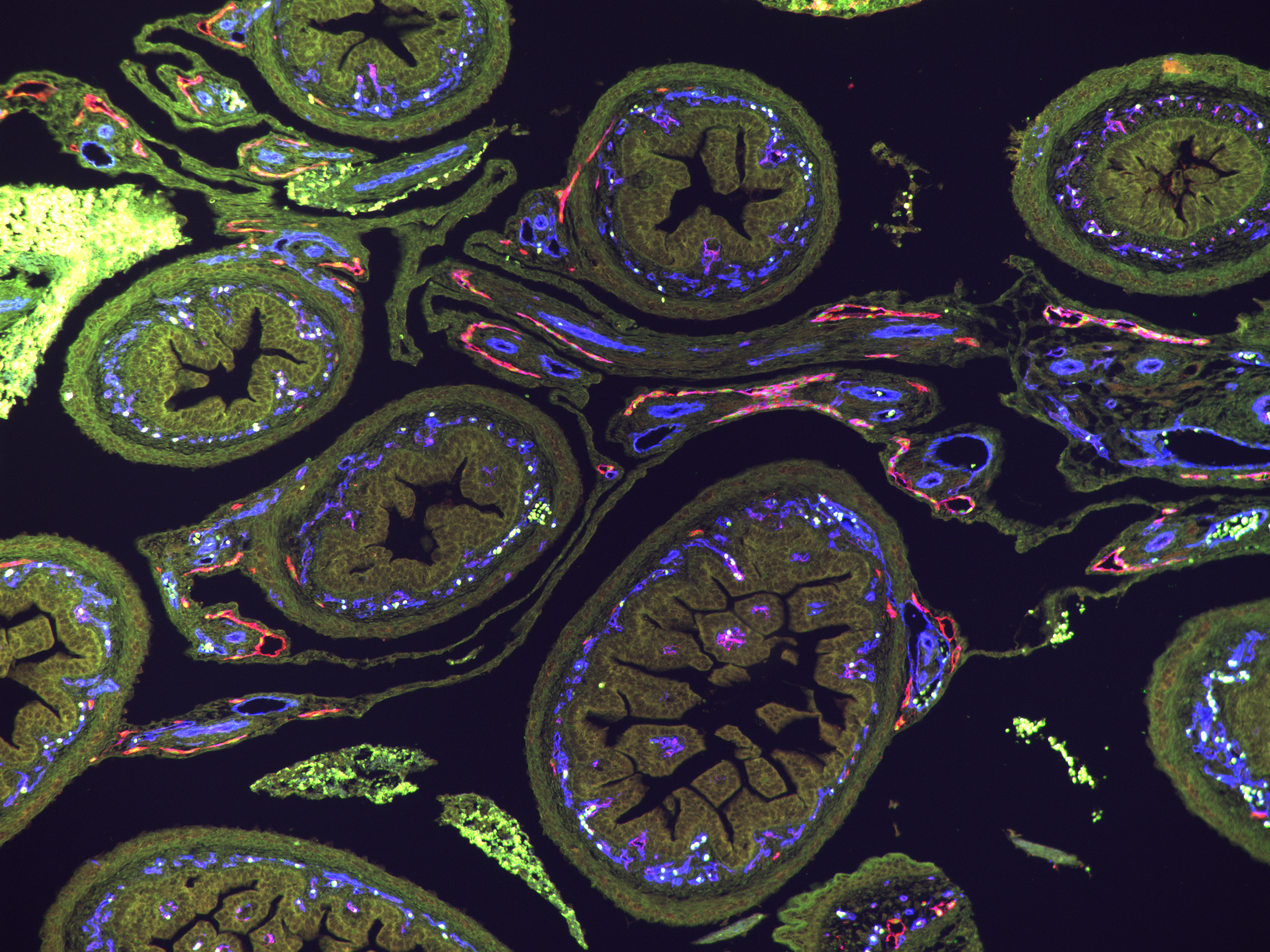

Supplement: Supplementary file 8 — Source data Fig. 2 [file 44321_2025_235_MOESM8_ESM.zip › Figure 2/Figure 2V.tif]

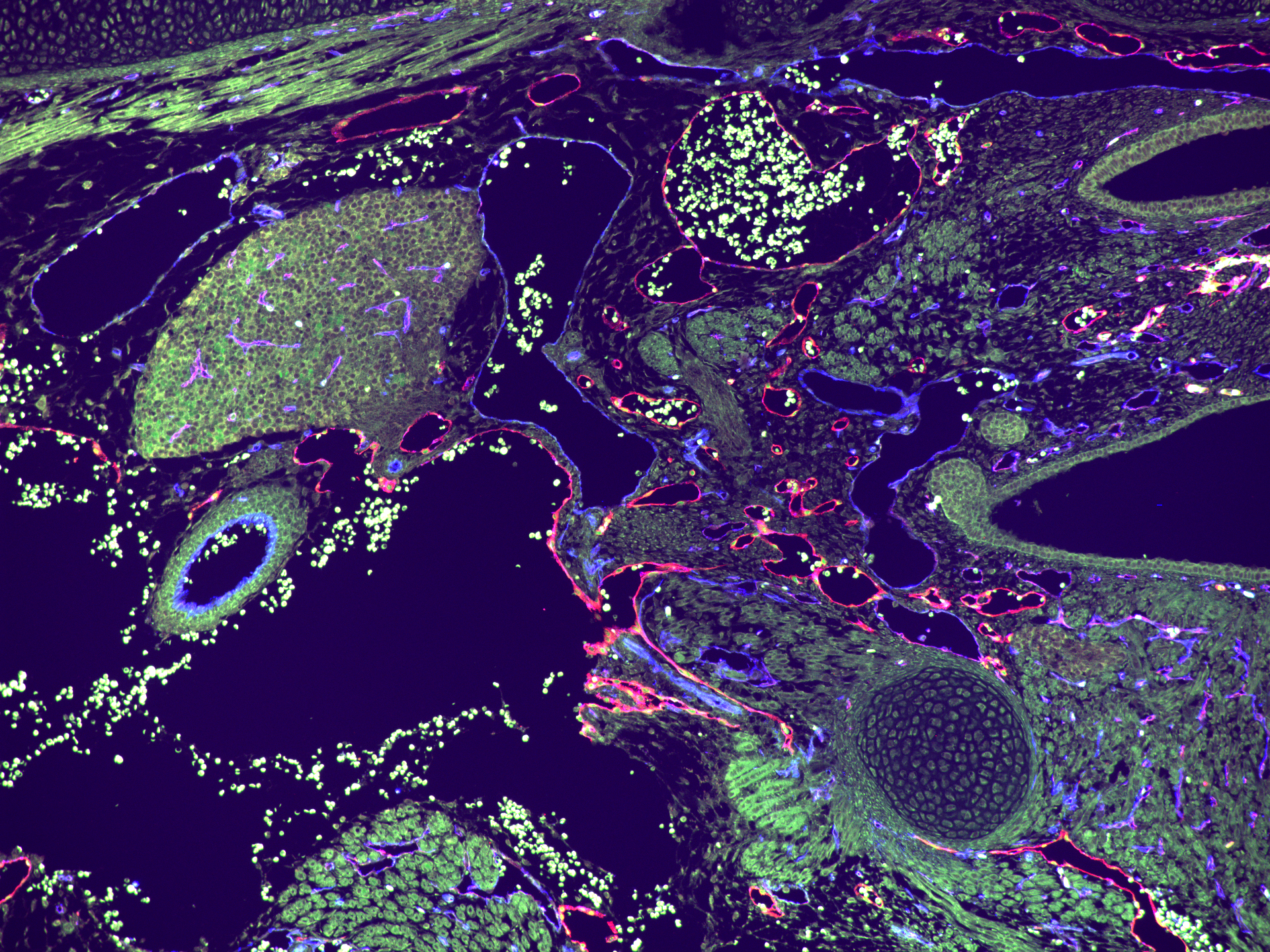

Supplement: Supplementary file 8 — Source data Fig. 2 [file 44321_2025_235_MOESM8_ESM.zip › Figure 2/Figure 2L.tif]

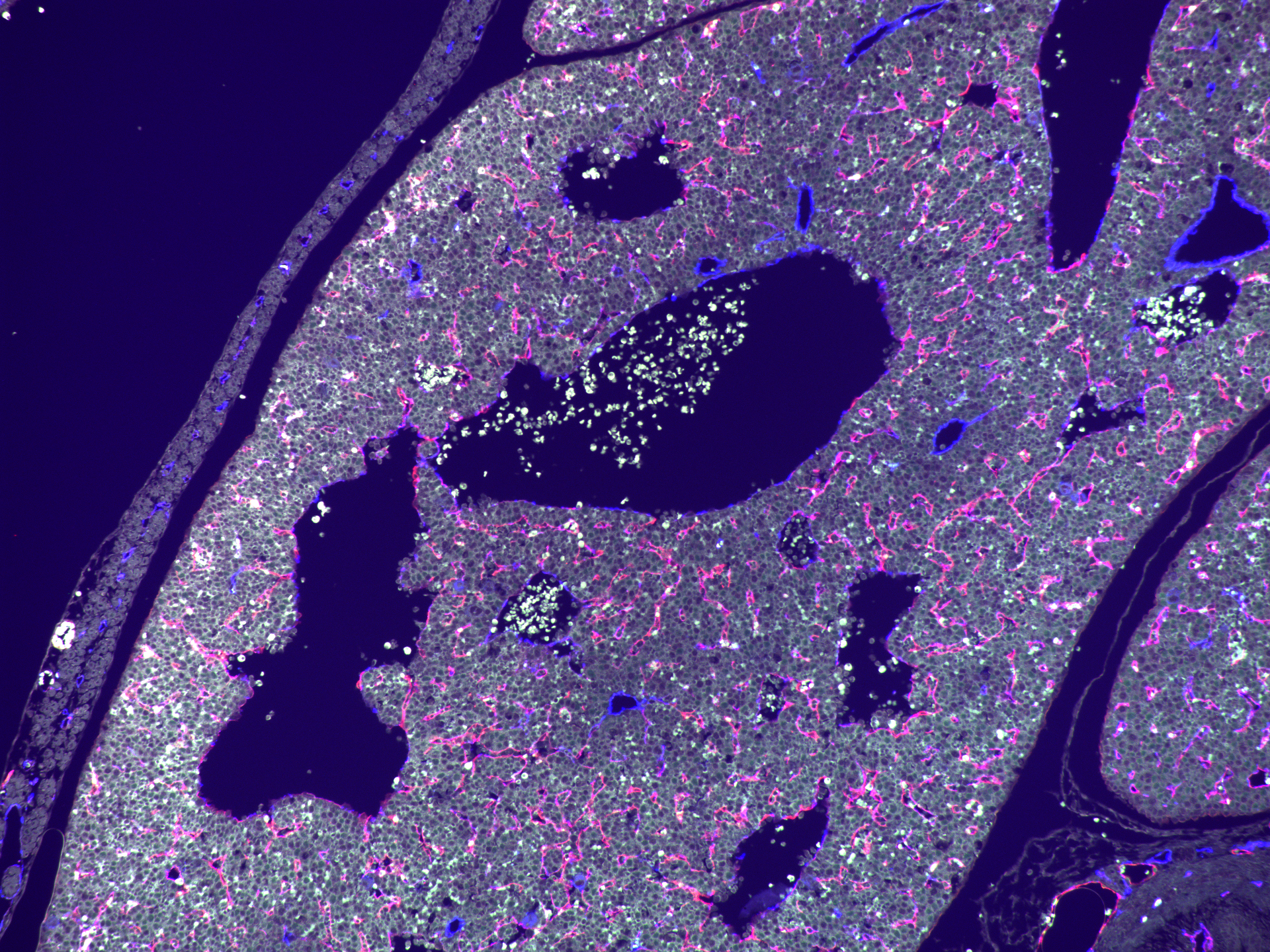

Supplement: Supplementary file 8 — Source data Fig. 2 [file 44321_2025_235_MOESM8_ESM.zip › Figure 2/Figure 2M.tif]

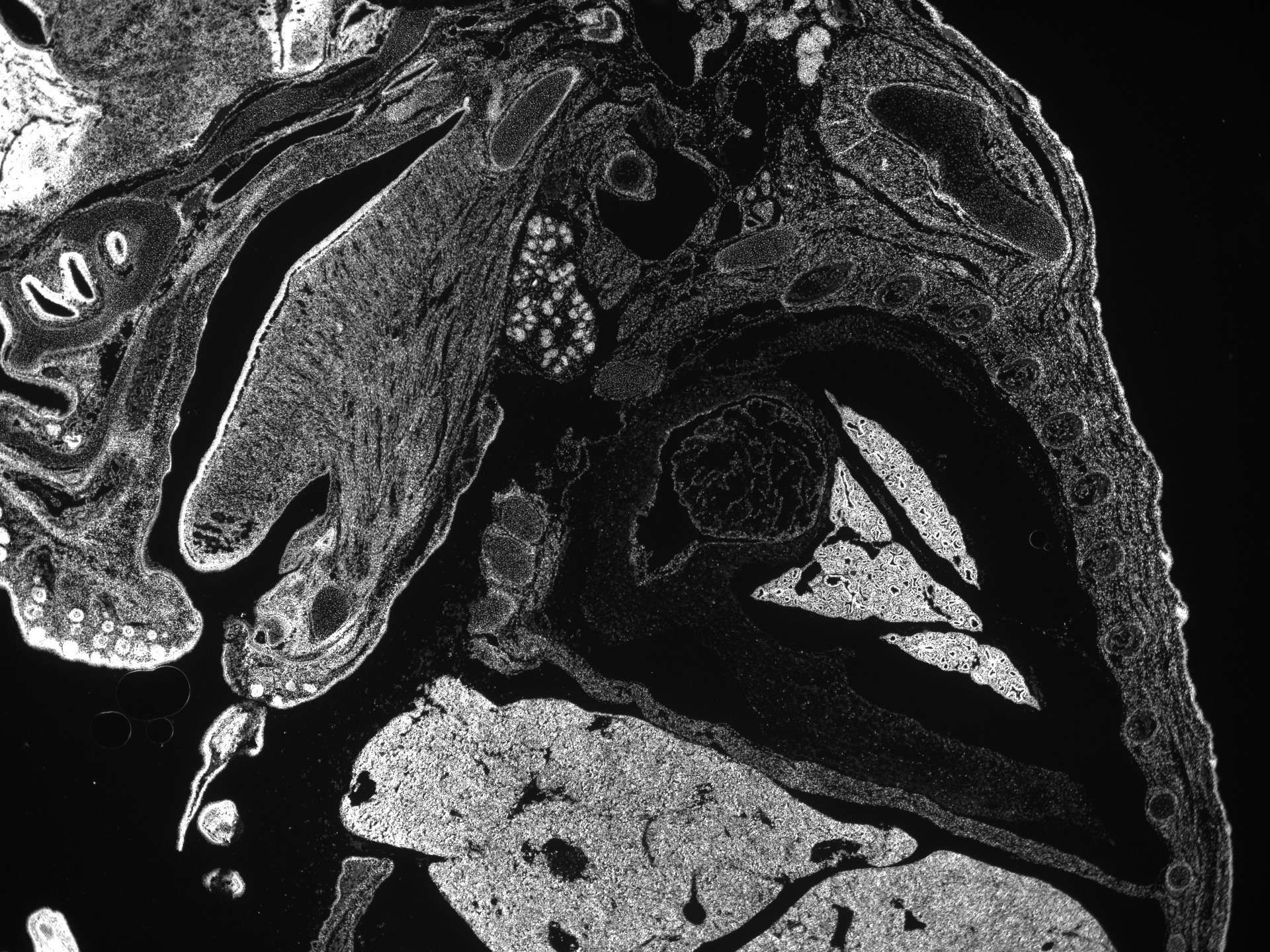

Supplement: Supplementary file 8 — Source data Fig. 2 [file 44321_2025_235_MOESM8_ESM.zip › Figure 2/Figure 2X.tif]

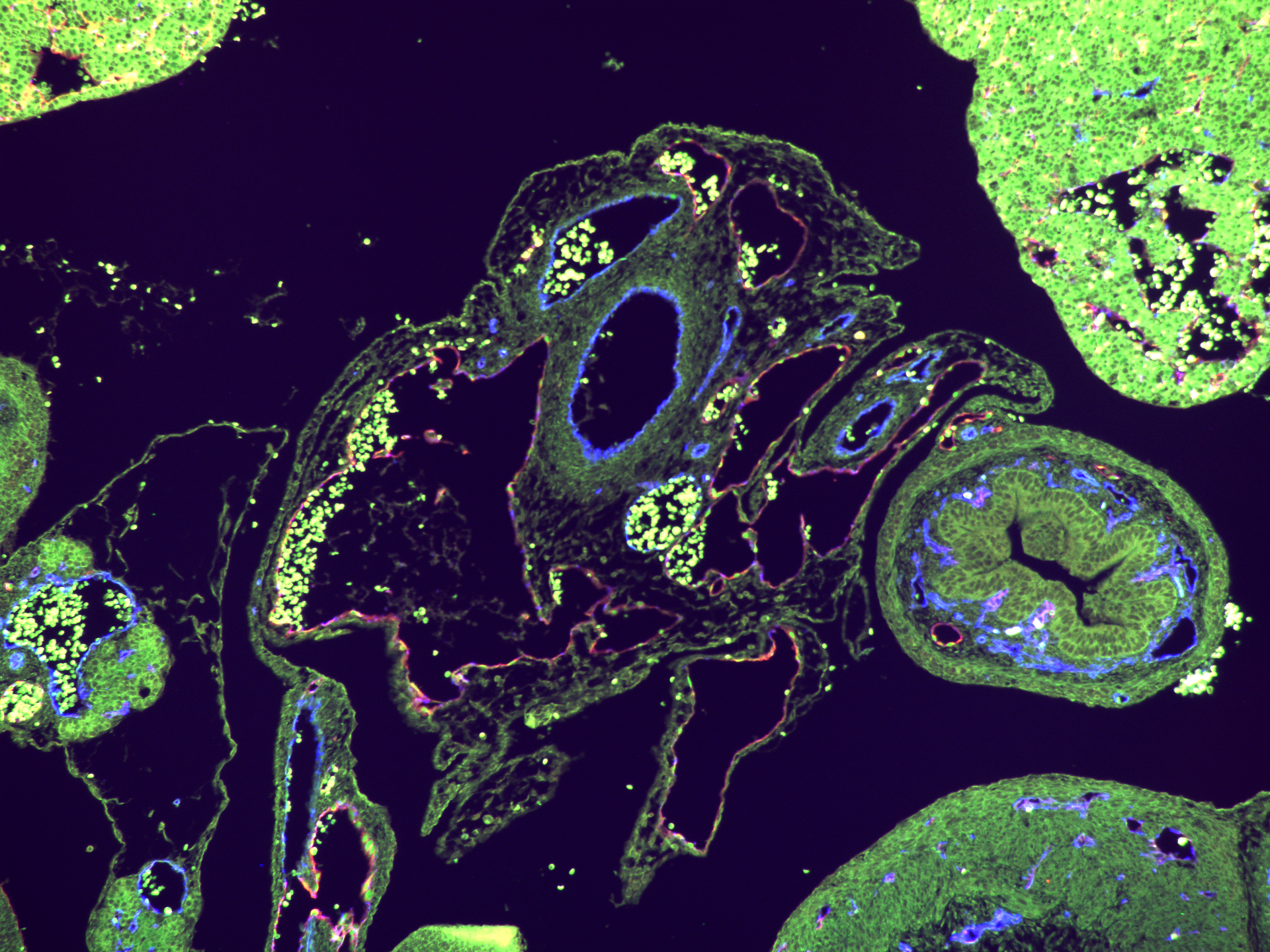

Supplement: Supplementary file 8 — Source data Fig. 2 [file 44321_2025_235_MOESM8_ESM.zip › Figure 2/Figure 2O.tif]

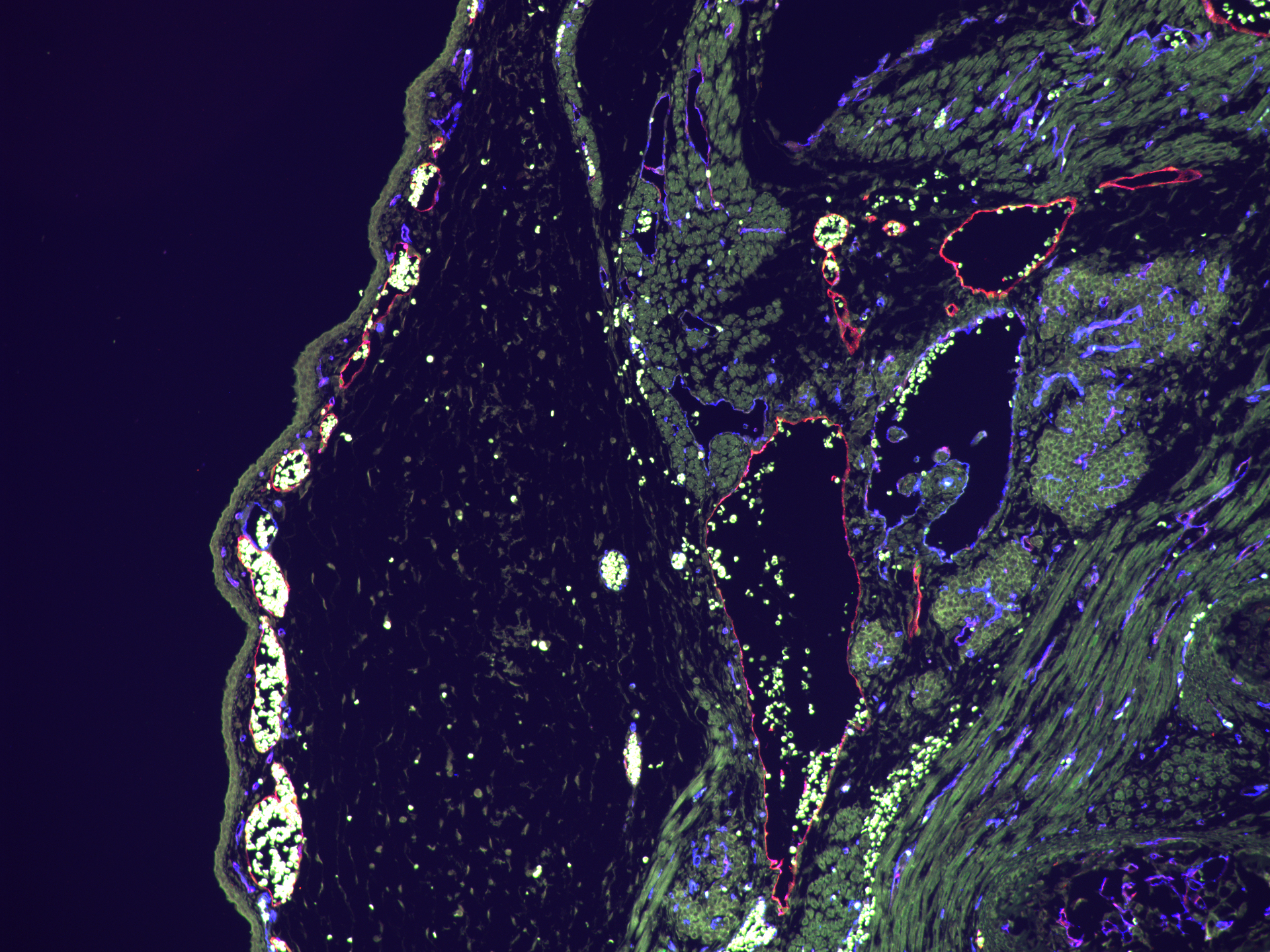

Supplement: Supplementary file 8 — Source data Fig. 2 [file 44321_2025_235_MOESM8_ESM.zip › Figure 2/Figure 2N.tif]

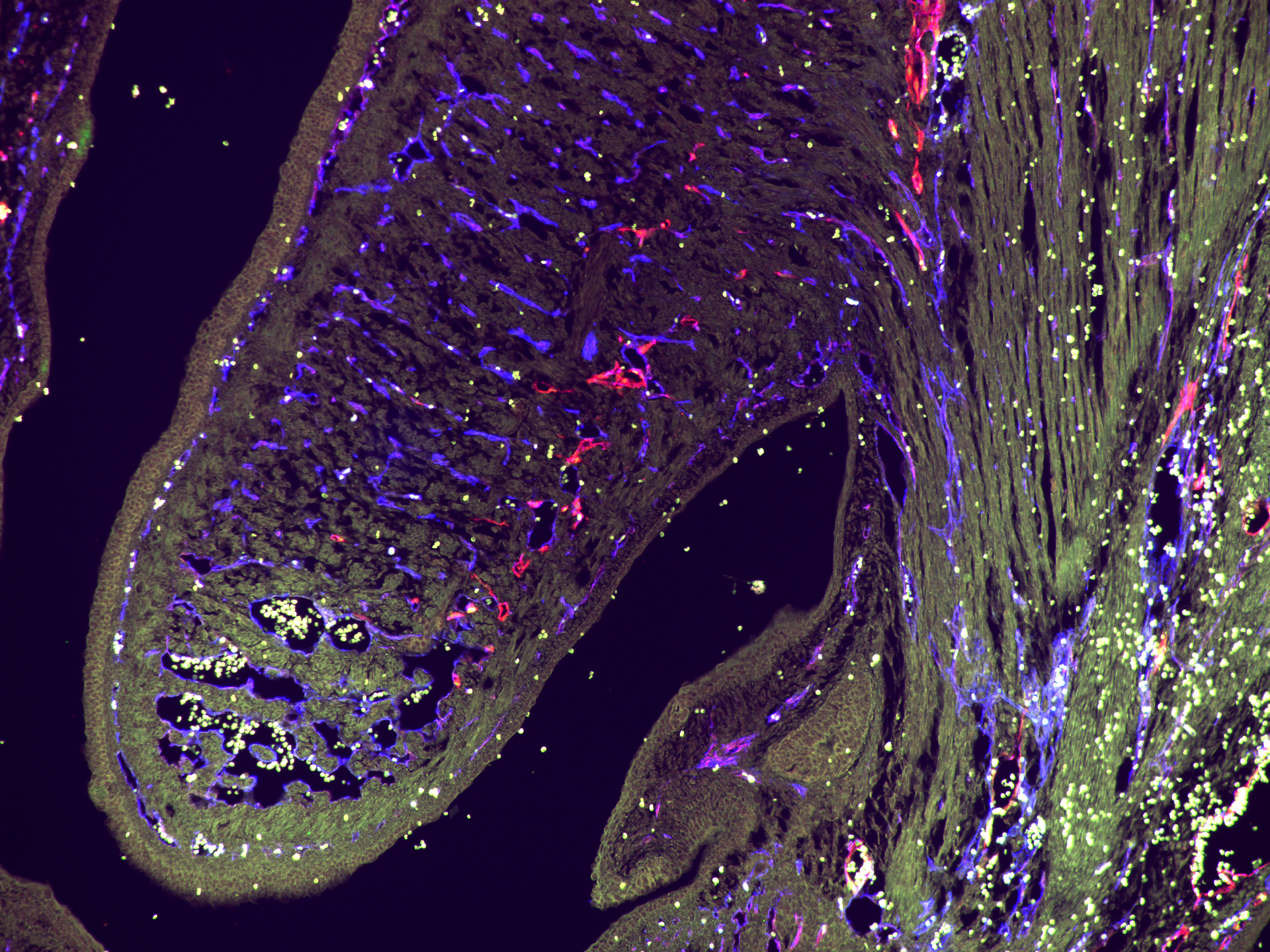

Supplement: Supplementary file 8 — Source data Fig. 2 [file 44321_2025_235_MOESM8_ESM.zip › Figure 2/Figure 2Y.tif]

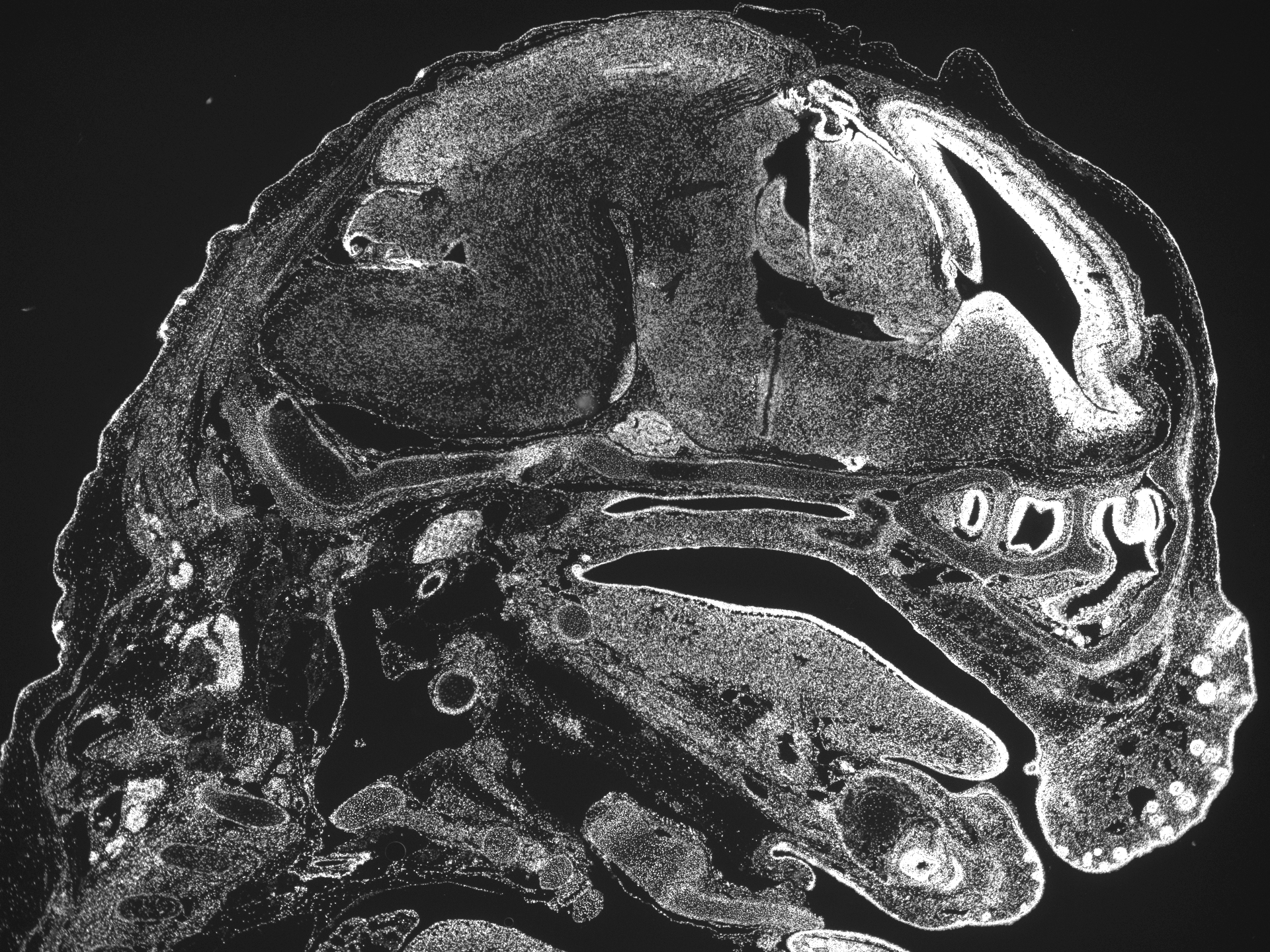

Supplement: Supplementary file 8 — Source data Fig. 2 [file 44321_2025_235_MOESM8_ESM.zip › Figure 2/Figure 2J.tif]

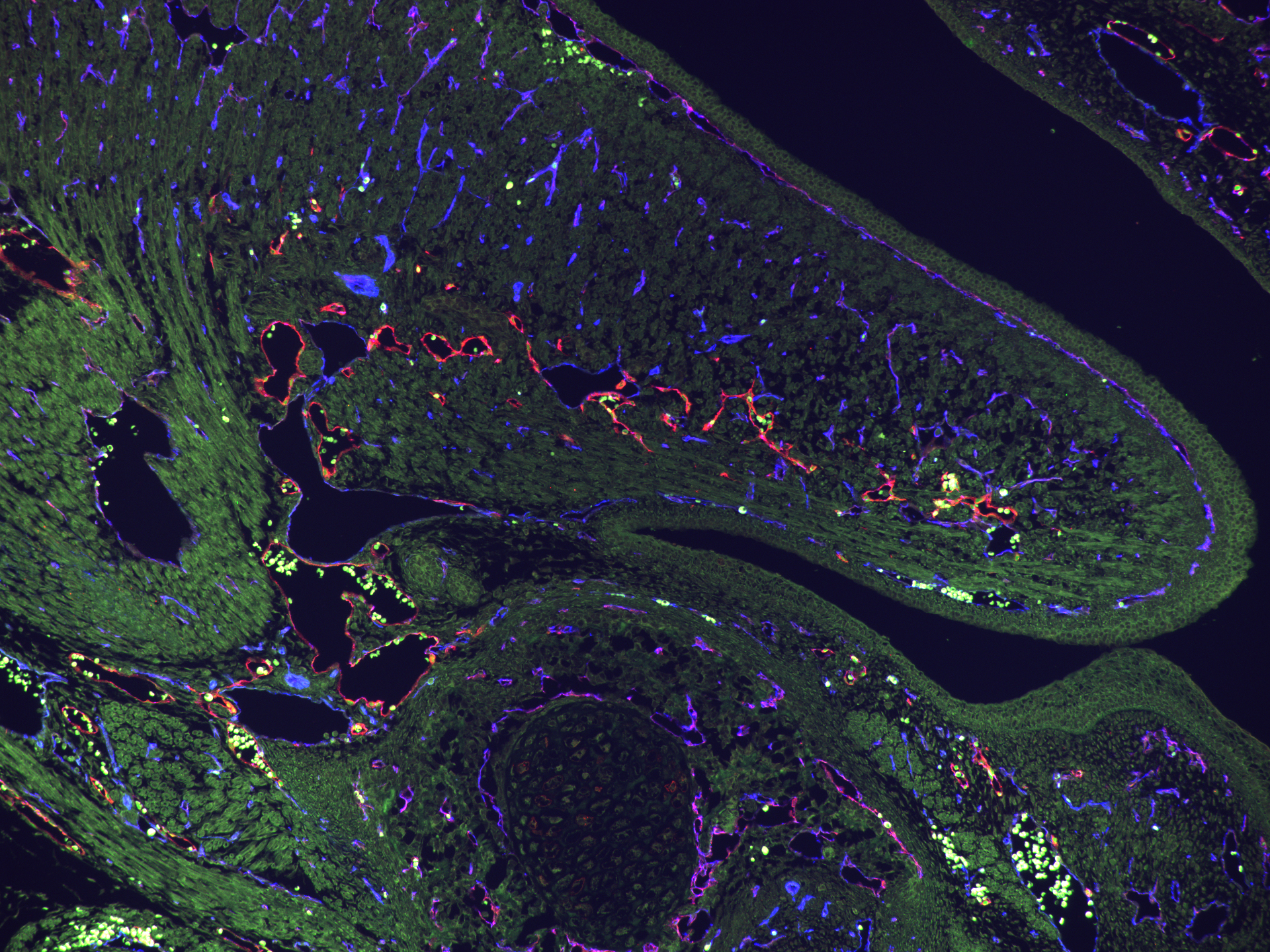

Supplement: Supplementary file 8 — Source data Fig. 2 [file 44321_2025_235_MOESM8_ESM.zip › Figure 2/Figure 2K.tif]

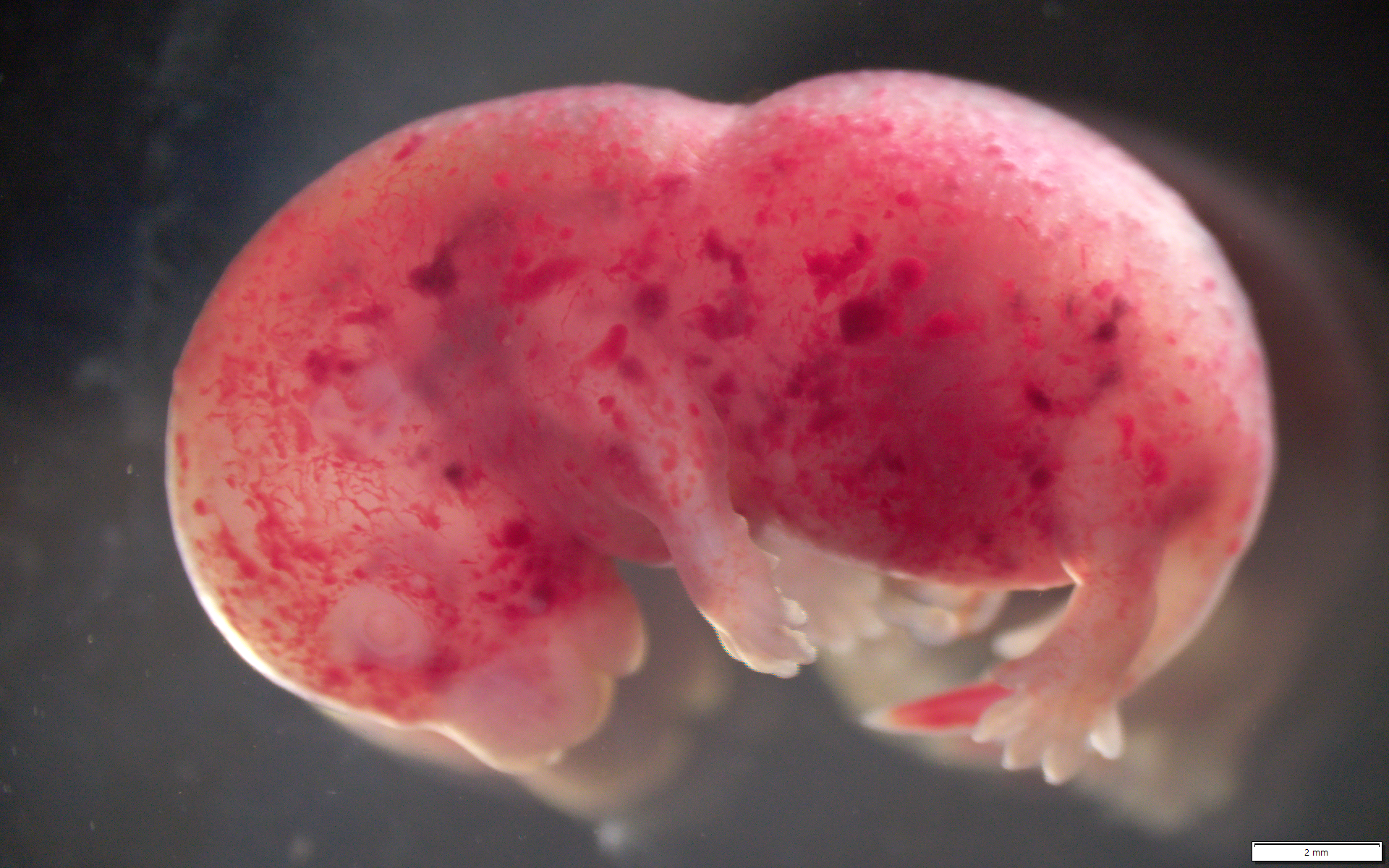

Supplement: Supplementary file 8 — Source data Fig. 2 [file 44321_2025_235_MOESM8_ESM.zip › Figure 2/figure 2I.tif]

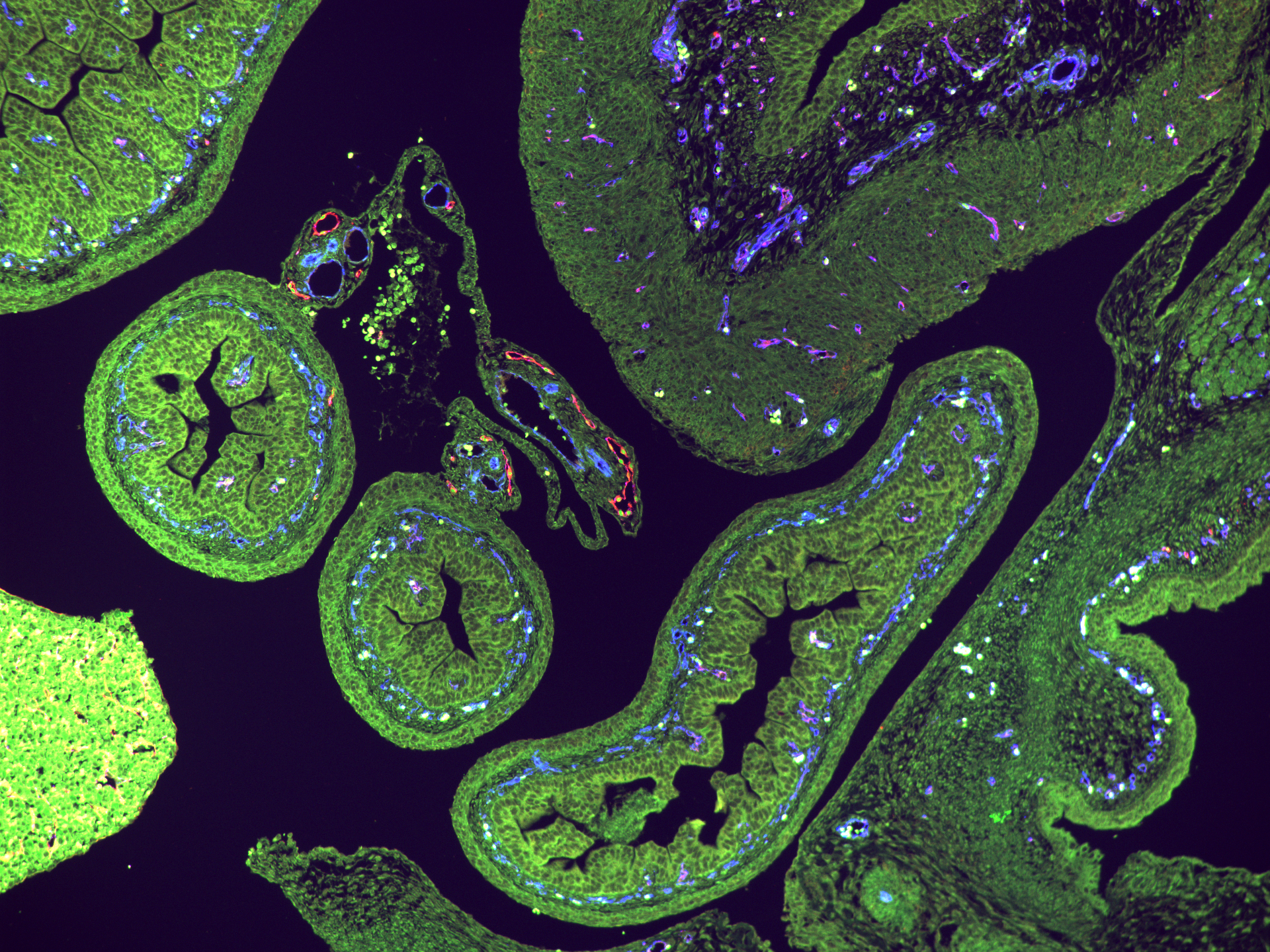

Supplement: Supplementary file 8 — Source data Fig. 2 [file 44321_2025_235_MOESM8_ESM.zip › Figure 2/Figure 2H.tif]

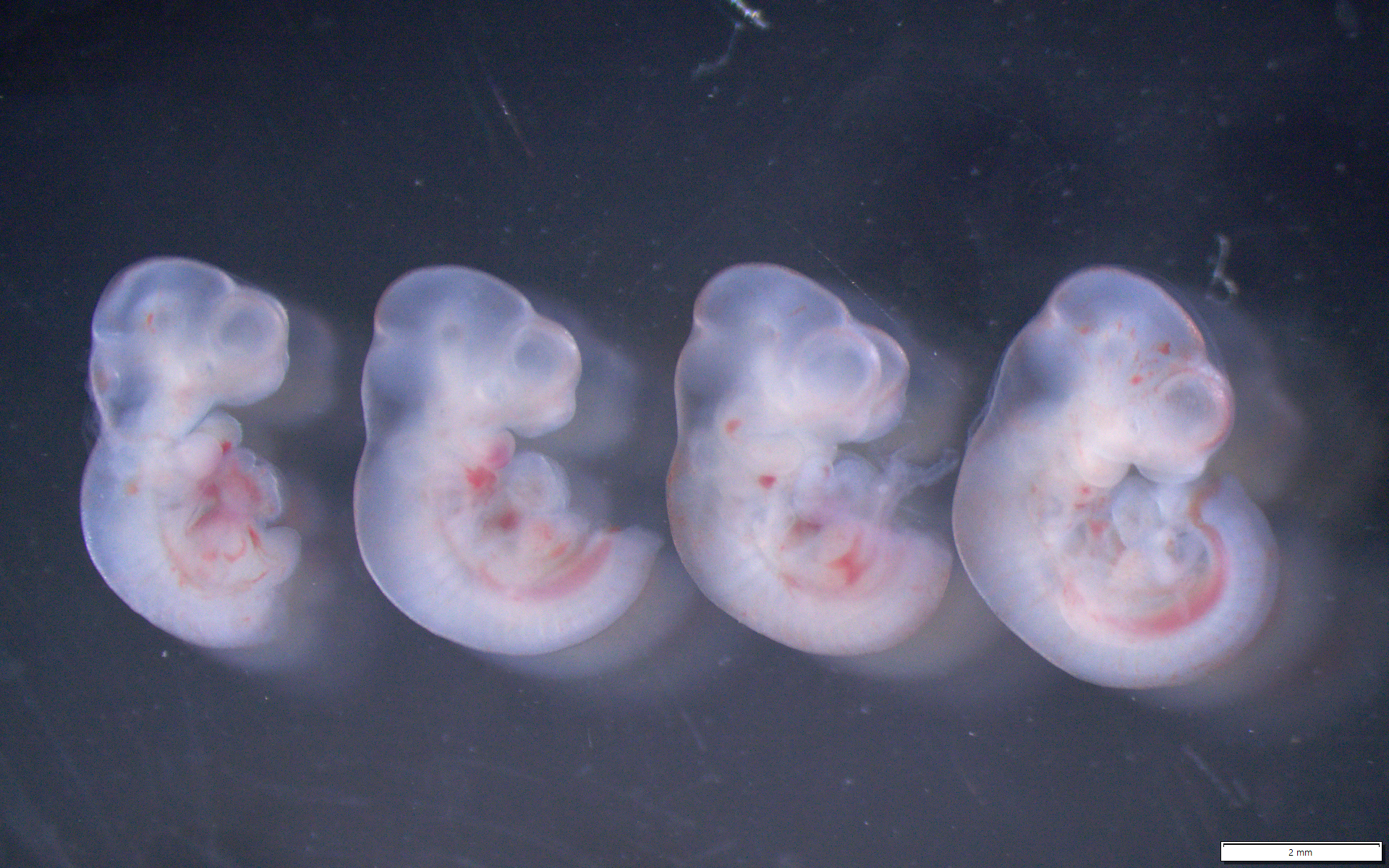

Supplement: Supplementary file 9 — Source data Fig. 3 [file 44321_2025_235_MOESM9_ESM.zip › Figure 3/Figure 3A.tif]

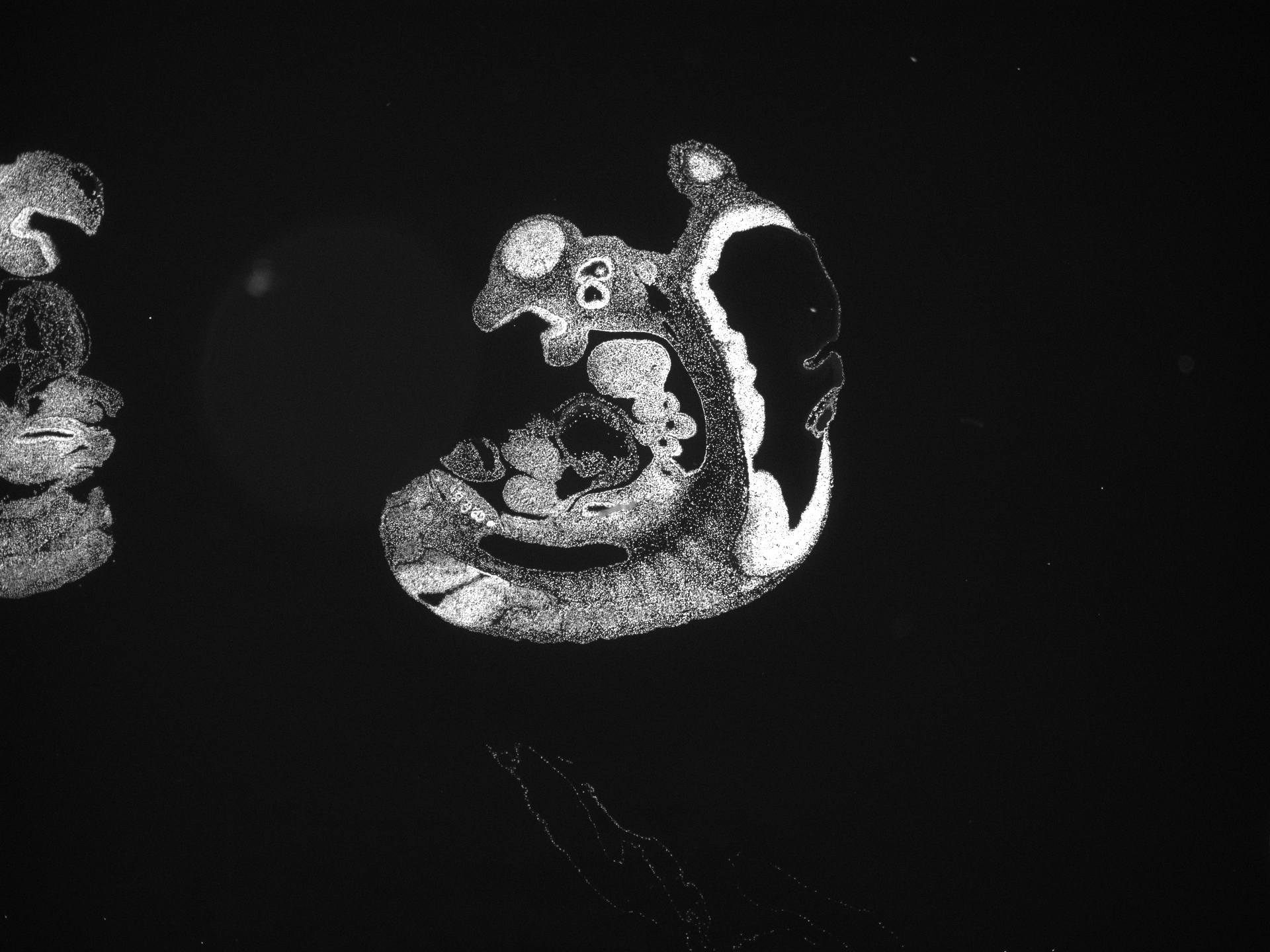

Supplement: Supplementary file 9 — Source data Fig. 3 [file 44321_2025_235_MOESM9_ESM.zip › Figure 3/Figure 3B.tif]

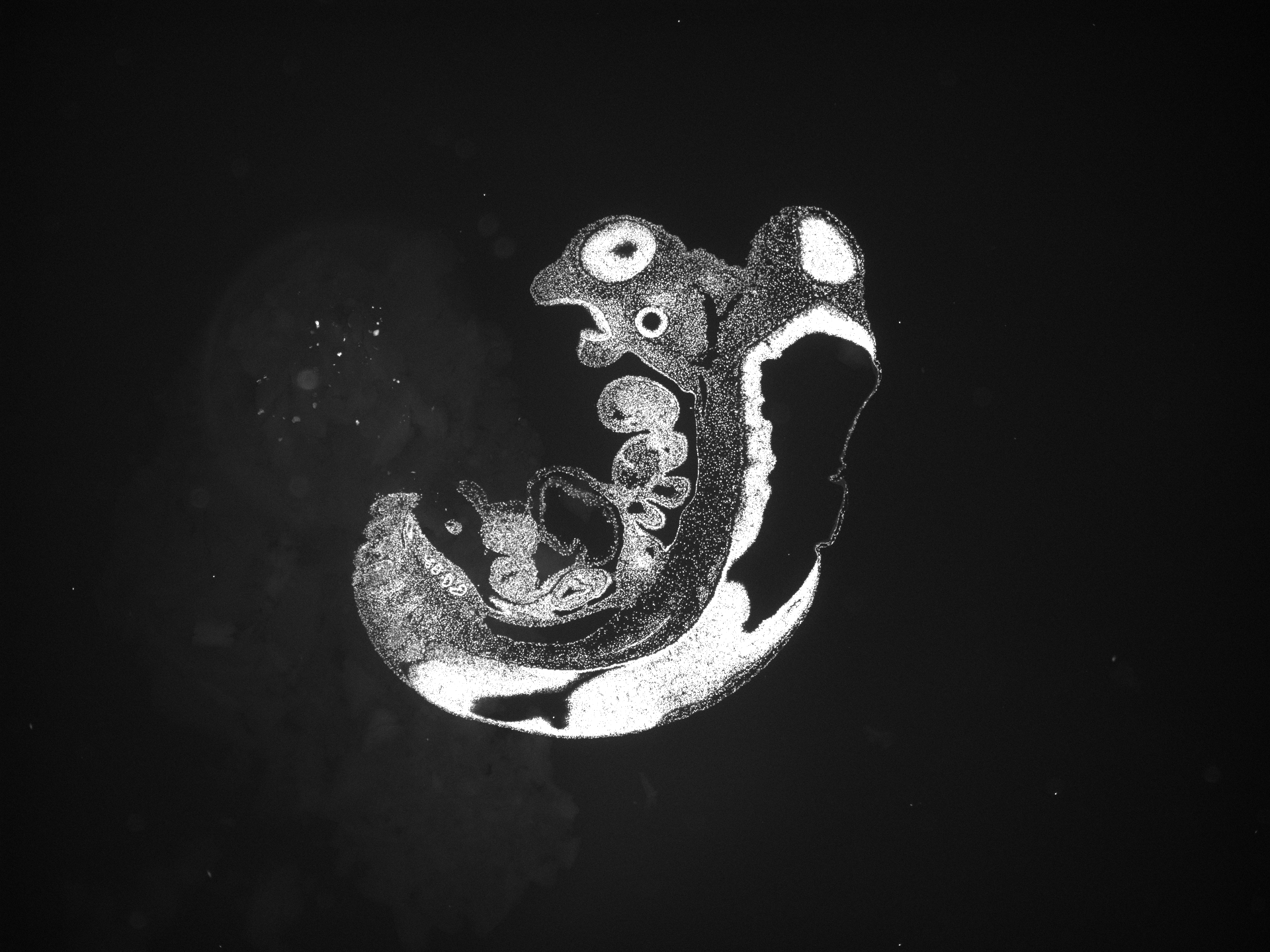

Supplement: Supplementary file 9 — Source data Fig. 3 [file 44321_2025_235_MOESM9_ESM.zip › Figure 3/Figure 3C.tif]

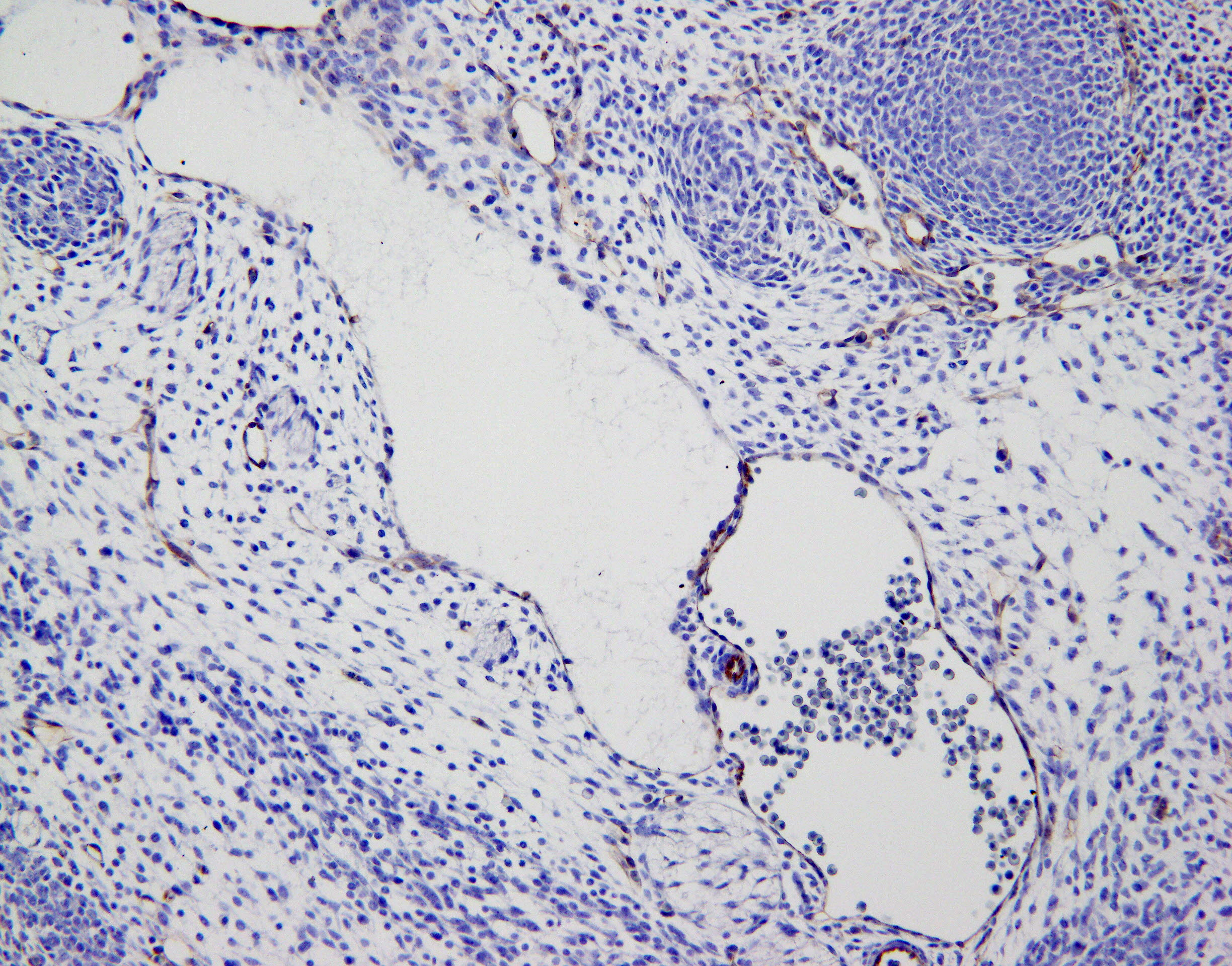

Supplement: Supplementary file 9 — Source data Fig. 3 [file 44321_2025_235_MOESM9_ESM.zip › Figure 3/Figure 3G.tif]

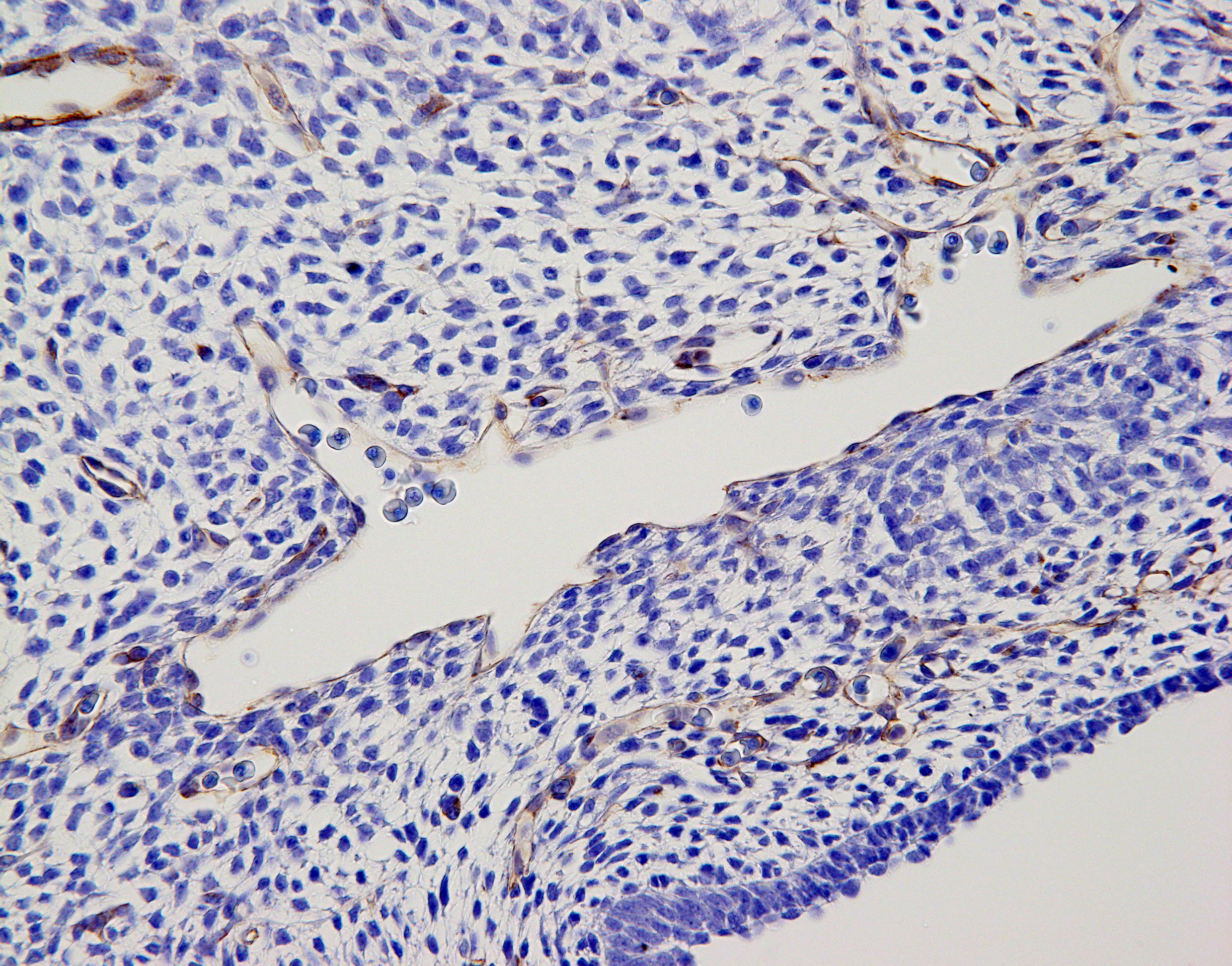

Supplement: Supplementary file 9 — Source data Fig. 3 [file 44321_2025_235_MOESM9_ESM.zip › Figure 3/Figure 3F.tif]

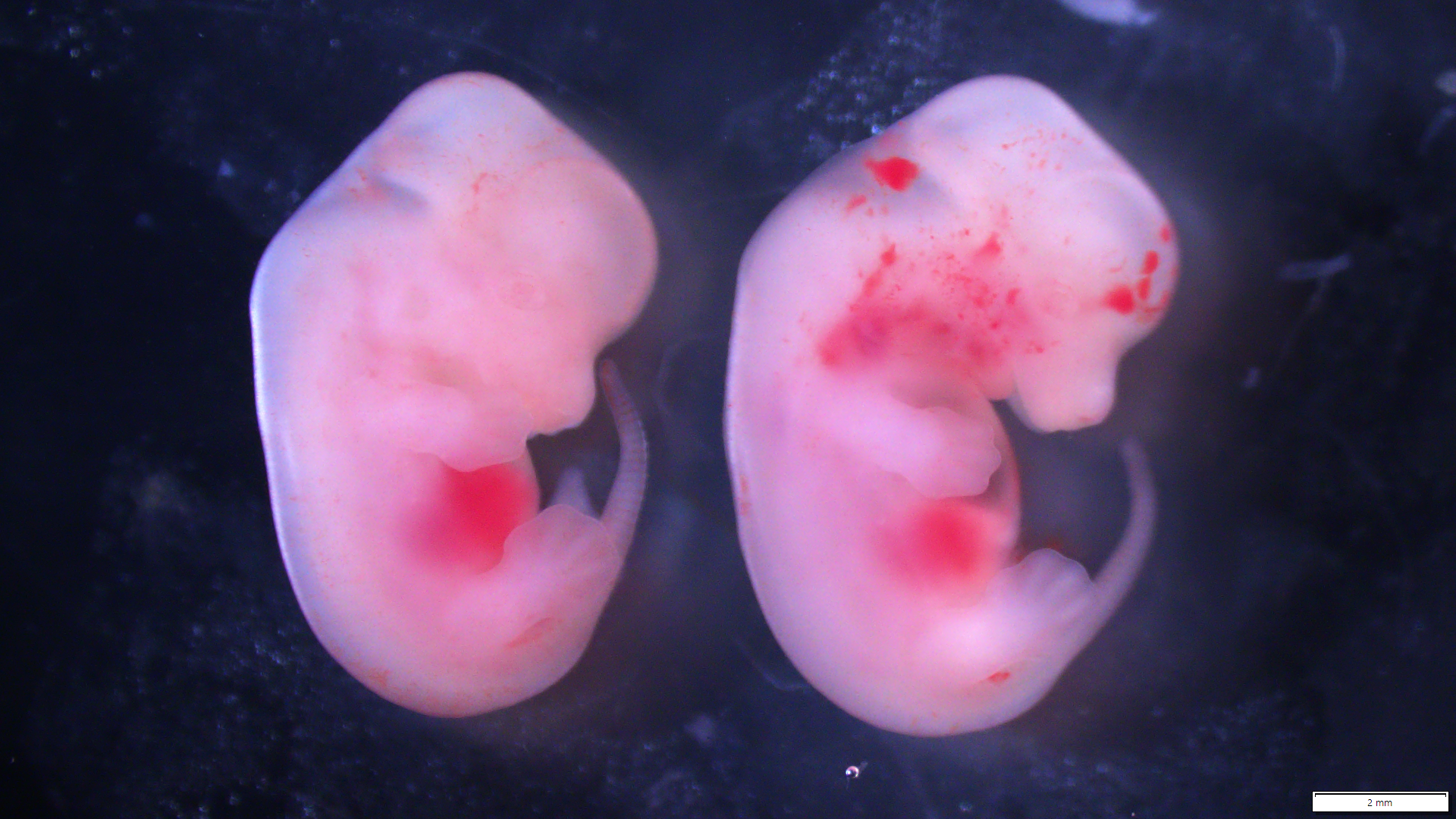

Supplement: Supplementary file 9 — Source data Fig. 3 [file 44321_2025_235_MOESM9_ESM.zip › Figure 3/Figure 3D.tif]

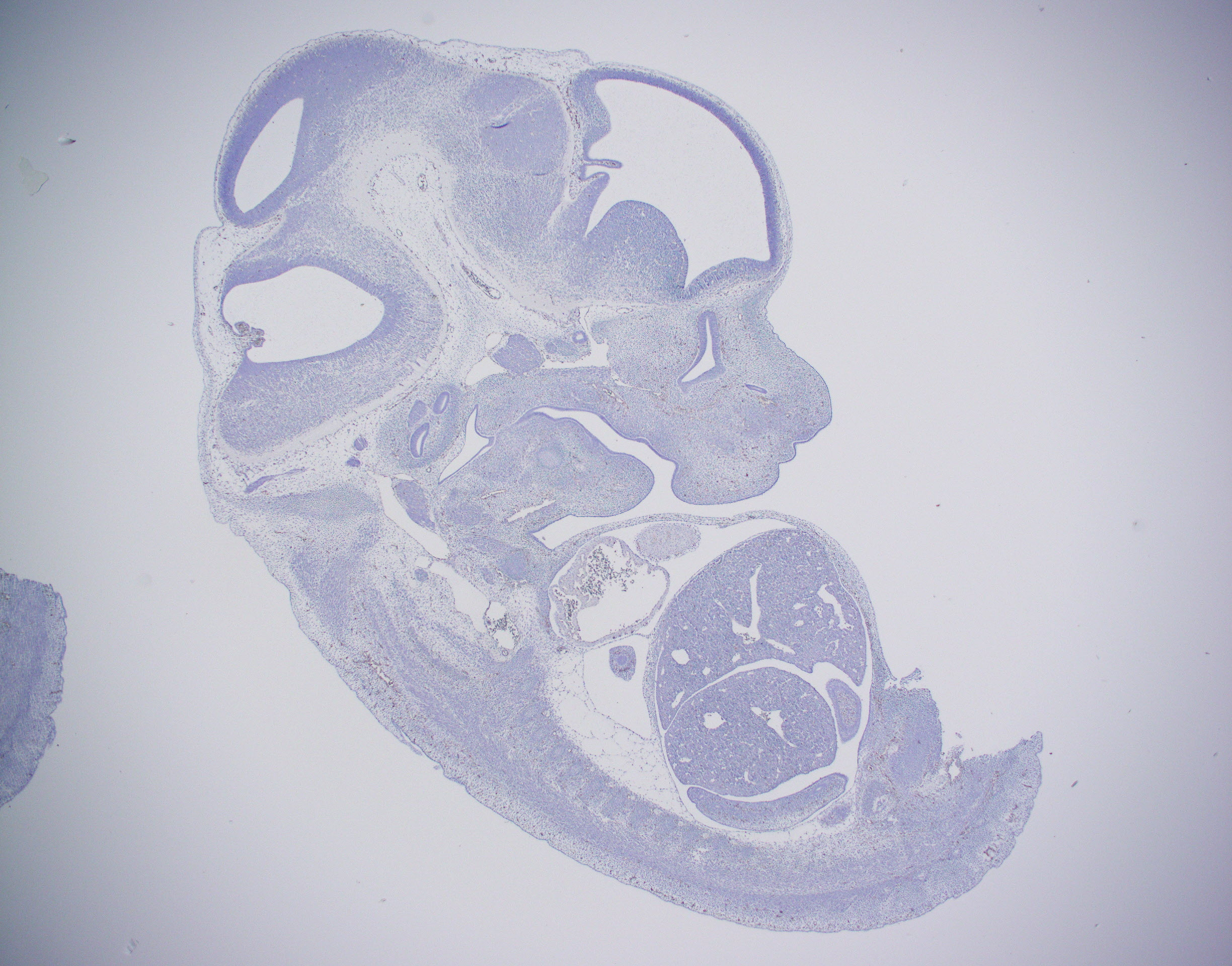

Supplement: Supplementary file 9 — Source data Fig. 3 [file 44321_2025_235_MOESM9_ESM.zip › Figure 3/Figure 3E.tif]

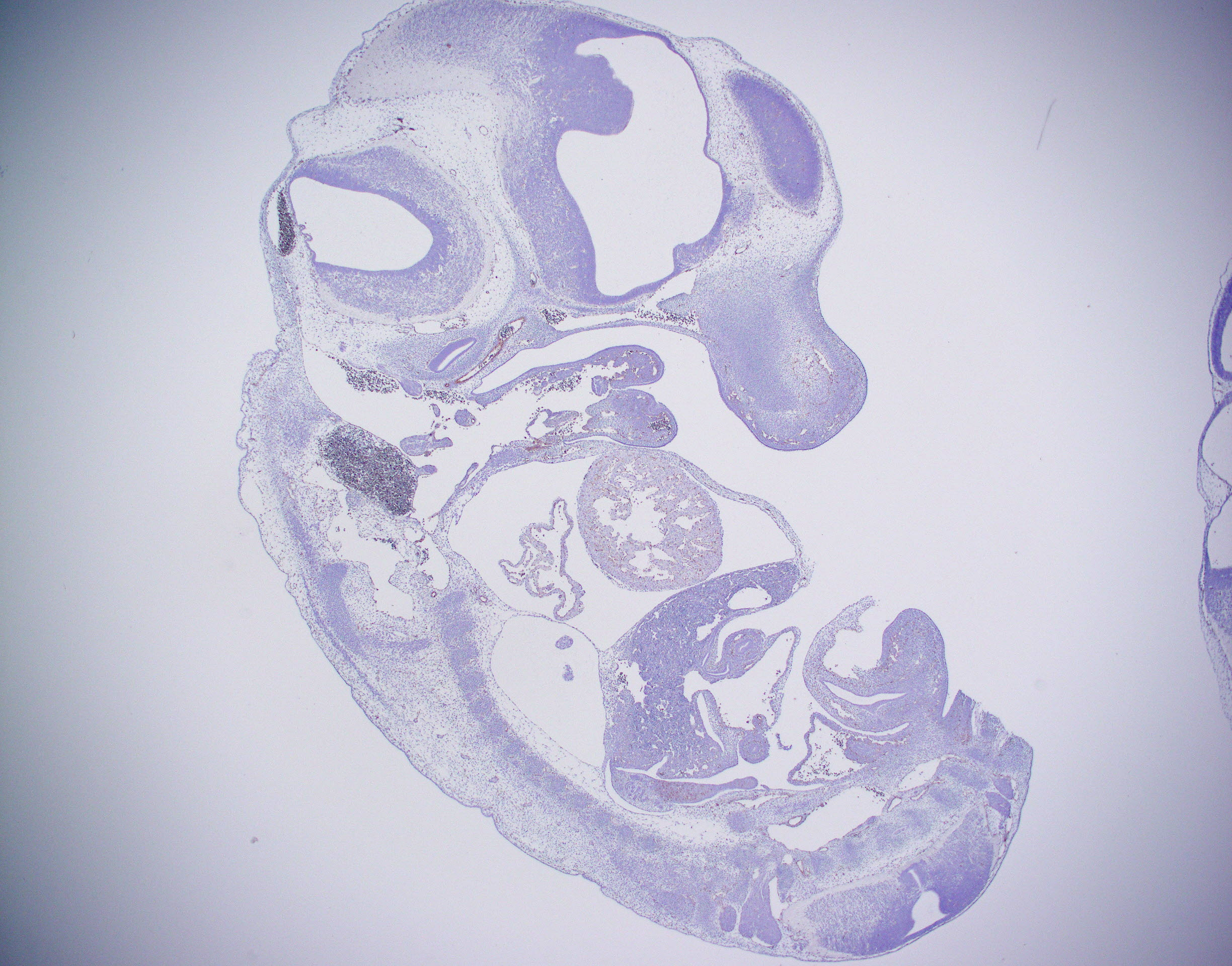

Supplement: Supplementary file 9 — Source data Fig. 3 [file 44321_2025_235_MOESM9_ESM.zip › Figure 3/Figure 3H.tif]

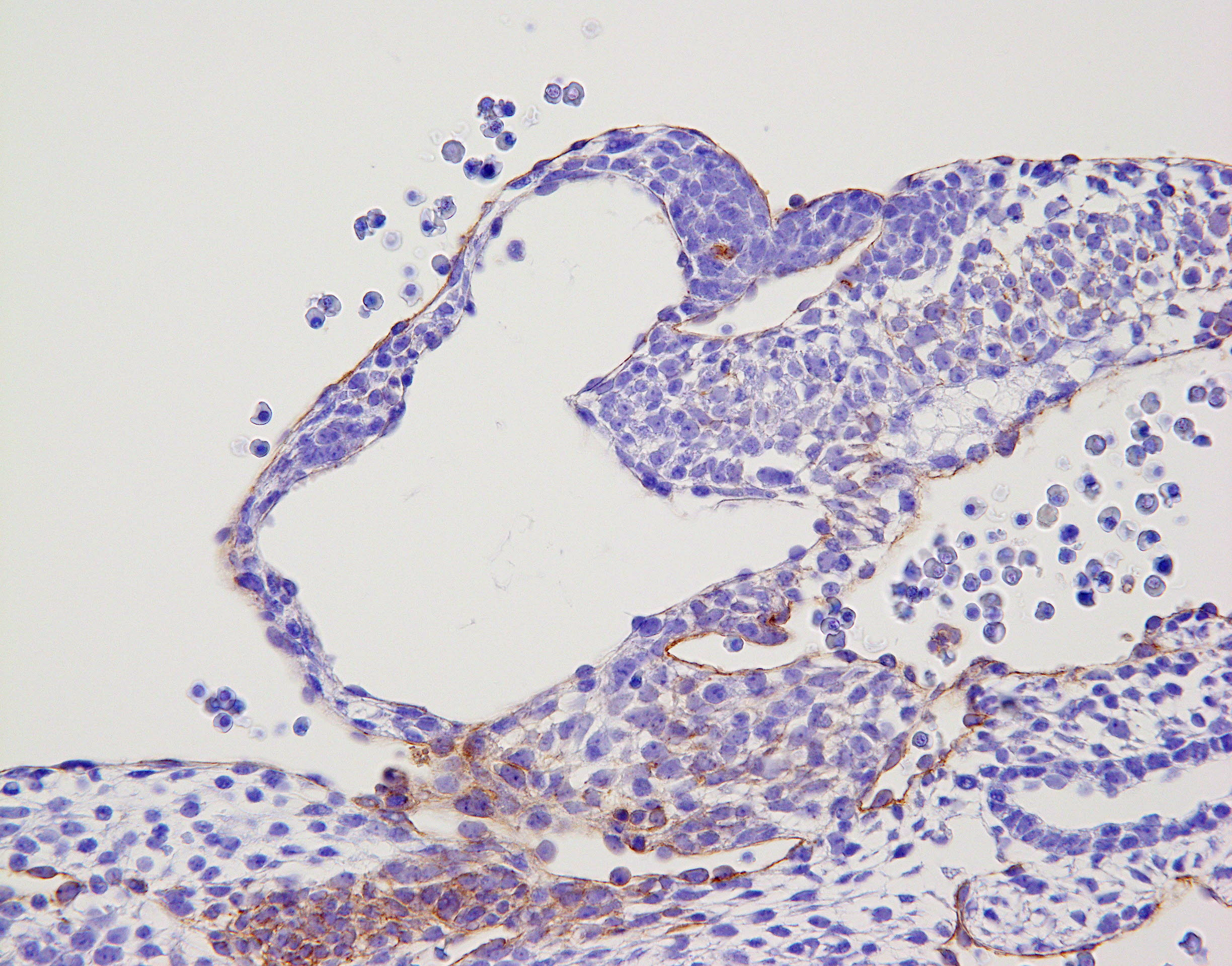

Supplement: Supplementary file 9 — Source data Fig. 3 [file 44321_2025_235_MOESM9_ESM.zip › Figure 3/figure 3I.tif]

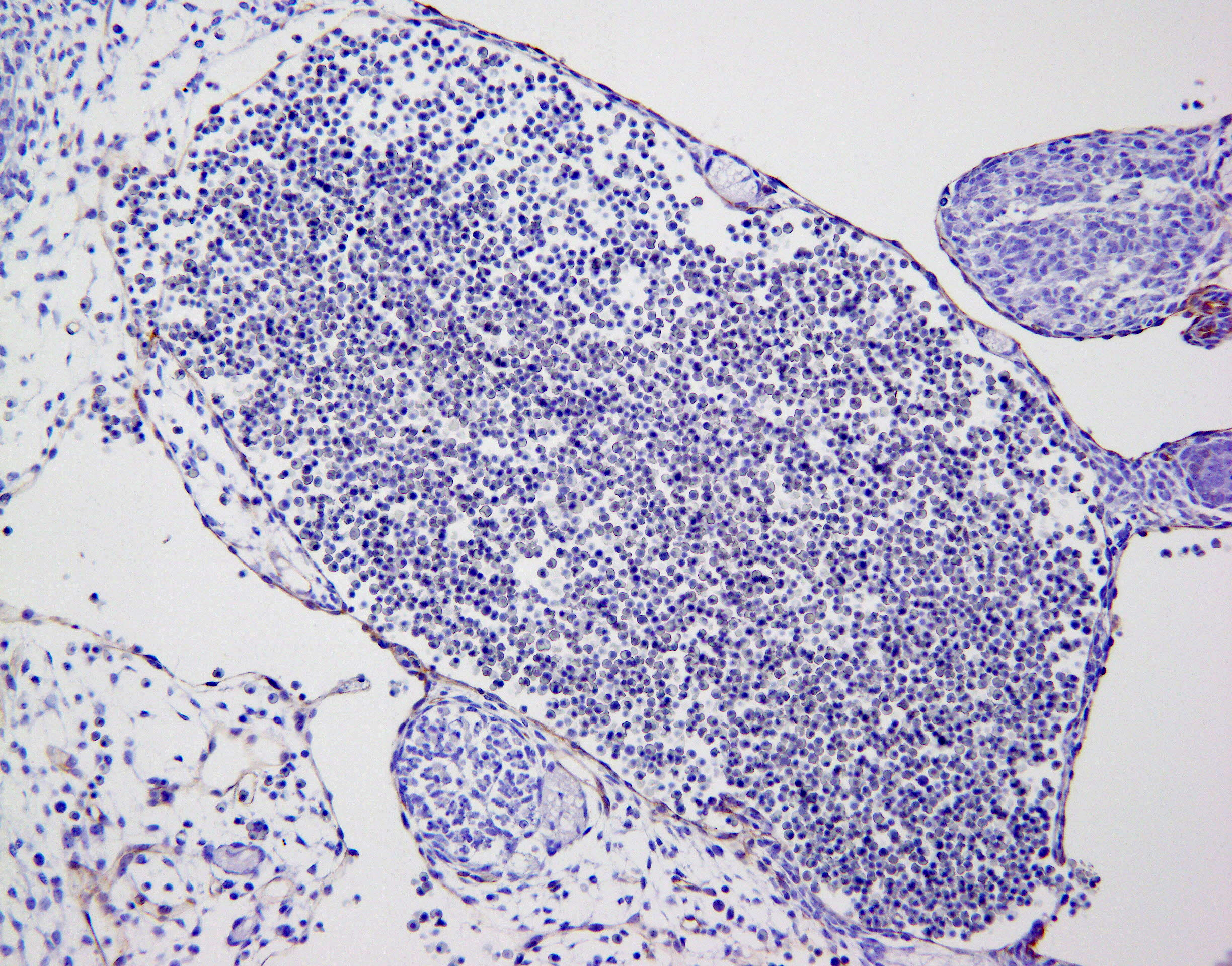

Supplement: Supplementary file 9 — Source data Fig. 3 [file 44321_2025_235_MOESM9_ESM.zip › Figure 3/Figure 3J.tif]

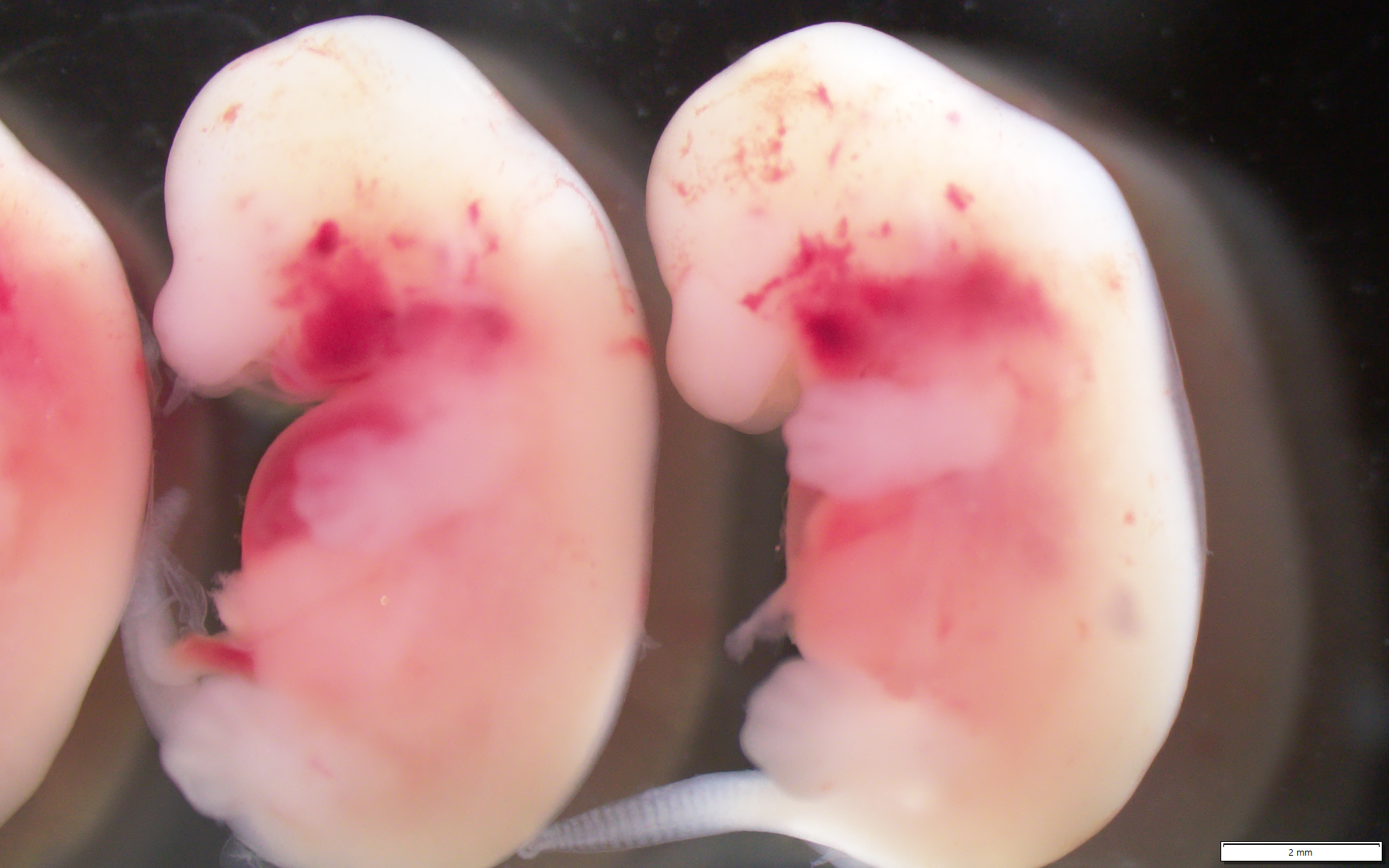

Supplement: Supplementary file 9 — Source data Fig. 3 [file 44321_2025_235_MOESM9_ESM.zip › Figure 3/Figure 3N.tif]

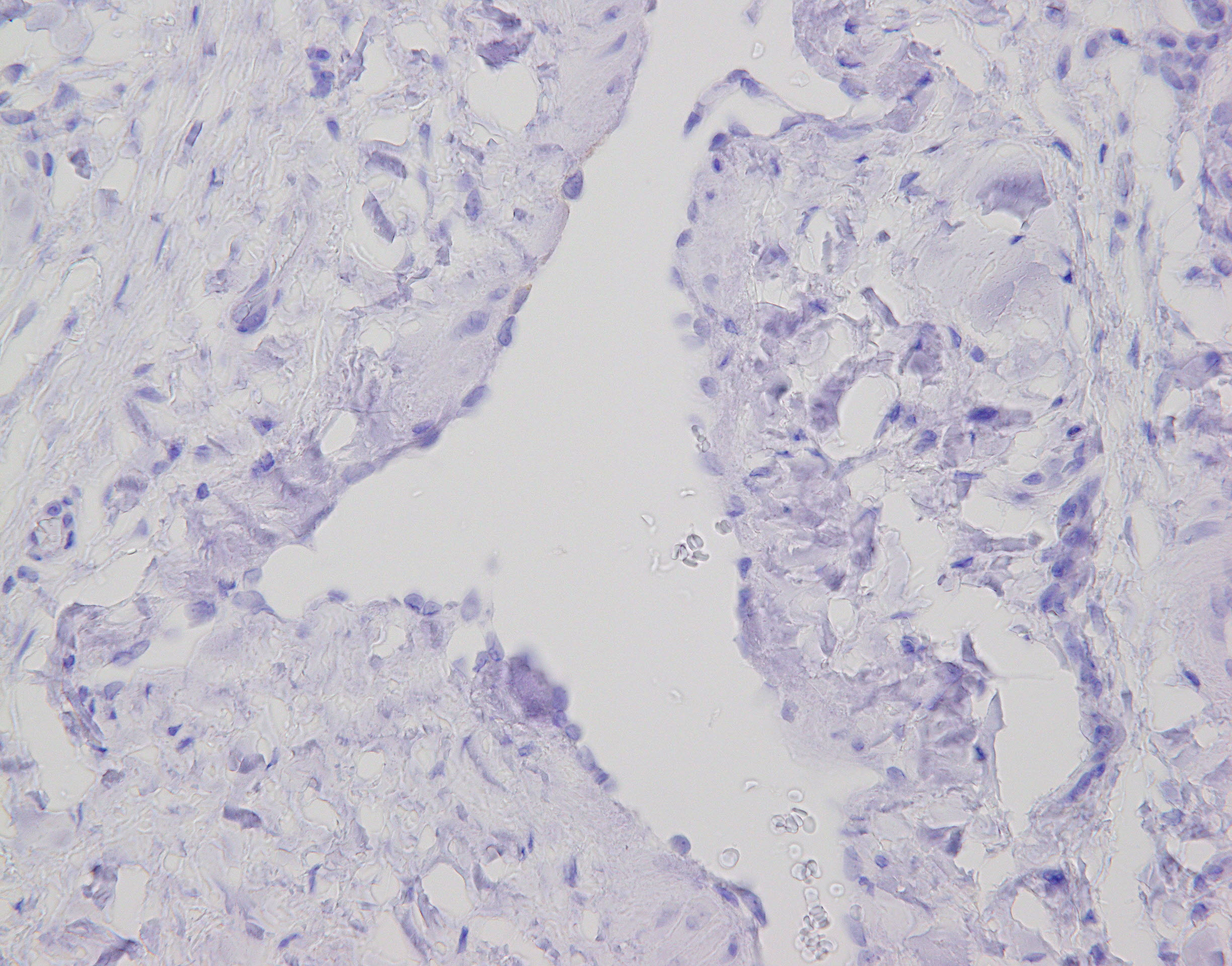

Supplement: Supplementary file 10 — Source data Fig. 5 [file 44321_2025_235_MOESM10_ESM.zip › Figure 5/Figure 5S.tif]

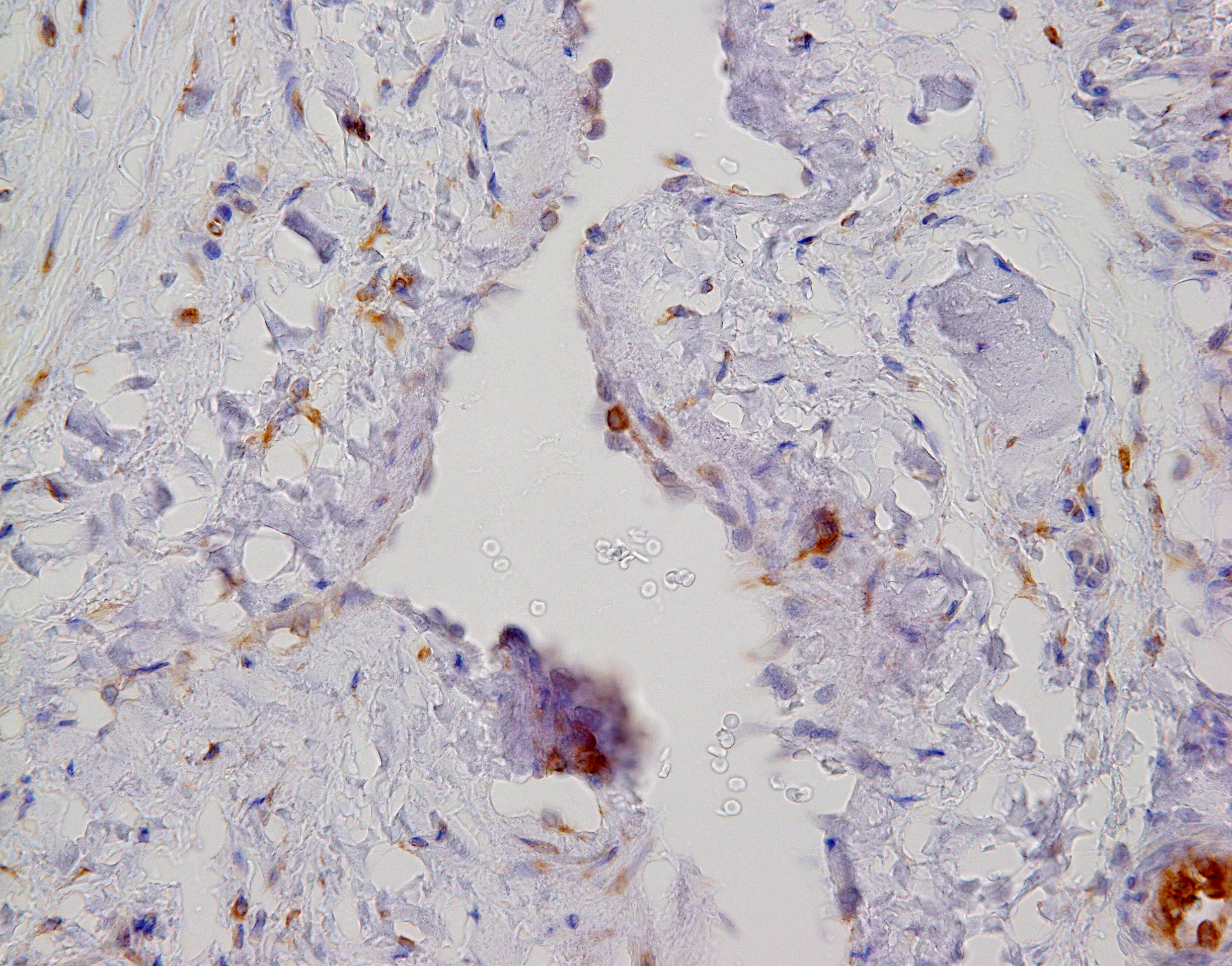

Supplement: Supplementary file 10 — Source data Fig. 5 [file 44321_2025_235_MOESM10_ESM.zip › Figure 5/Figure 5R.tif]

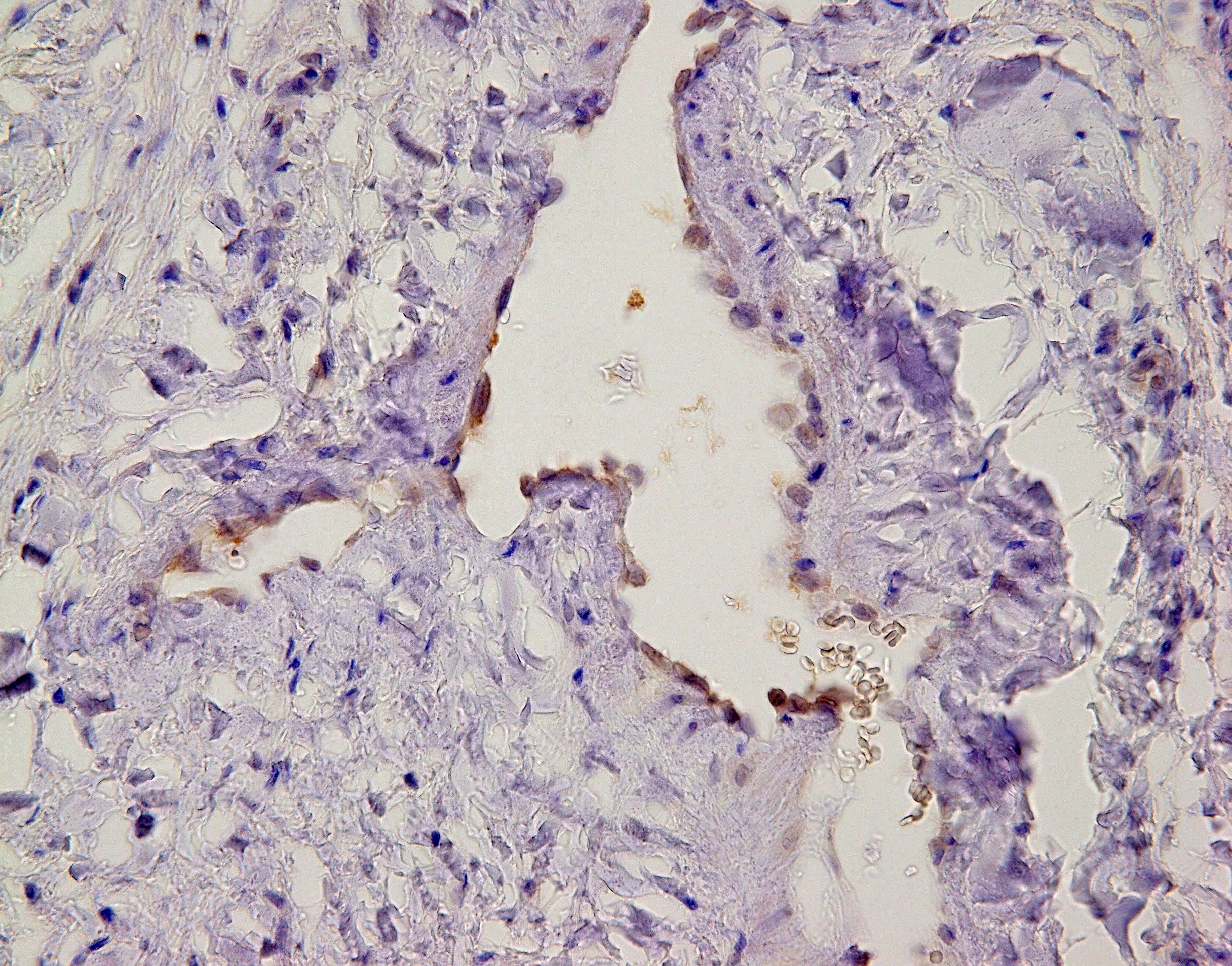

Supplement: Supplementary file 10 — Source data Fig. 5 [file 44321_2025_235_MOESM10_ESM.zip › Figure 5/Figure 5P.tif]

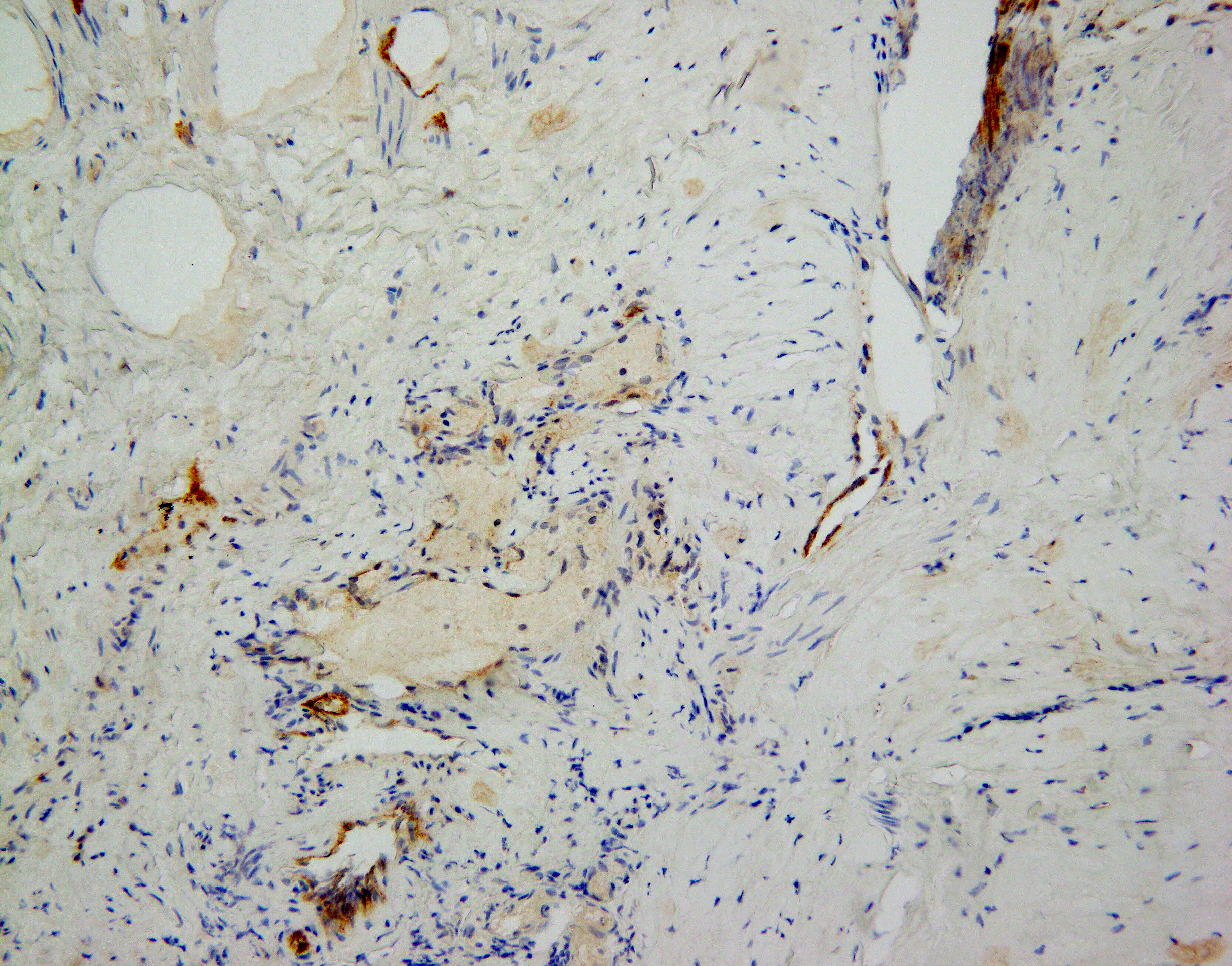

Supplement: Supplementary file 10 — Source data Fig. 5 [file 44321_2025_235_MOESM10_ESM.zip › Figure 5/Figure 5G.tif]

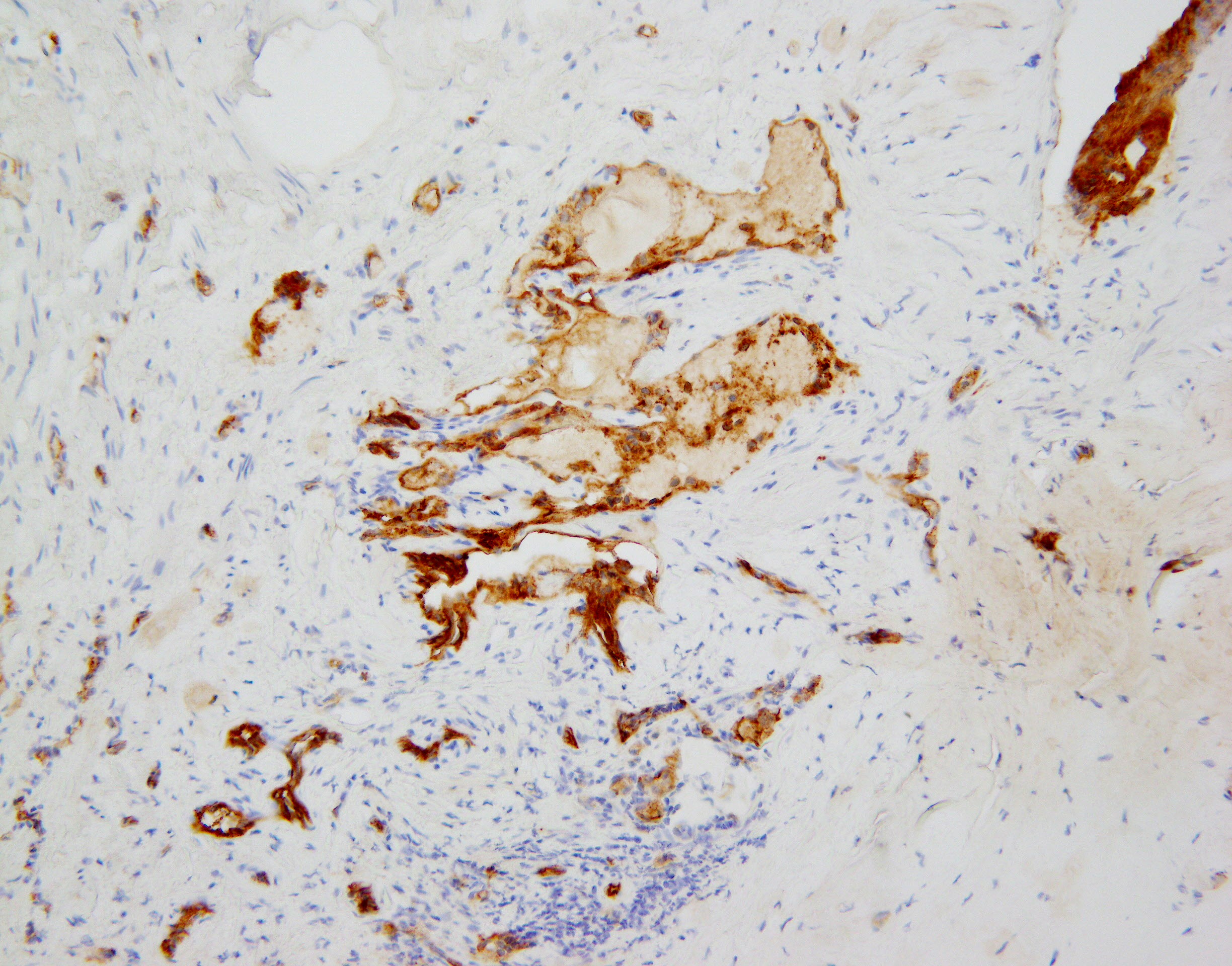

Supplement: Supplementary file 10 — Source data Fig. 5 [file 44321_2025_235_MOESM10_ESM.zip › Figure 5/Figure 5F.tif]

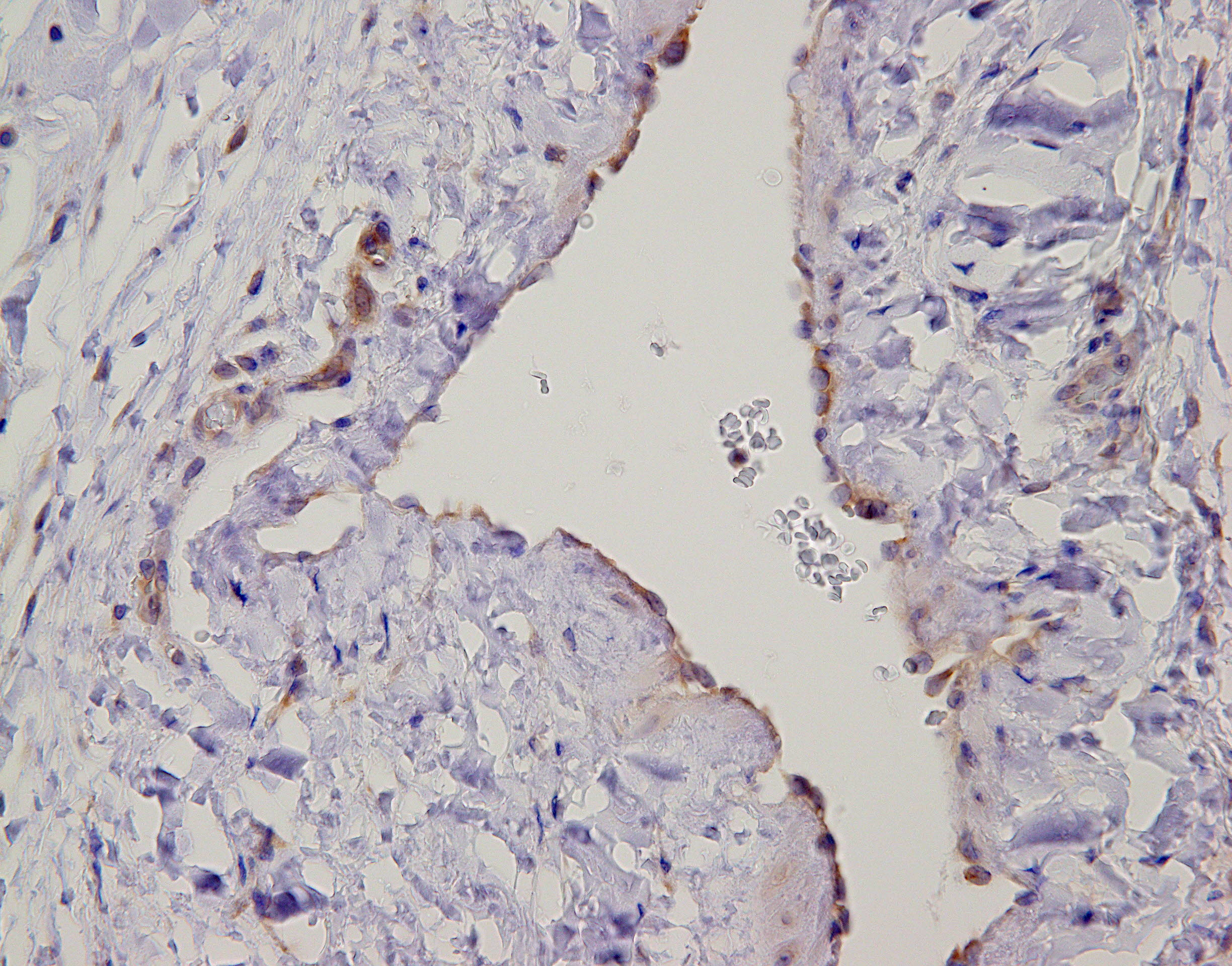

Supplement: Supplementary file 10 — Source data Fig. 5 [file 44321_2025_235_MOESM10_ESM.zip › Figure 5/Figure 5Q.tif]

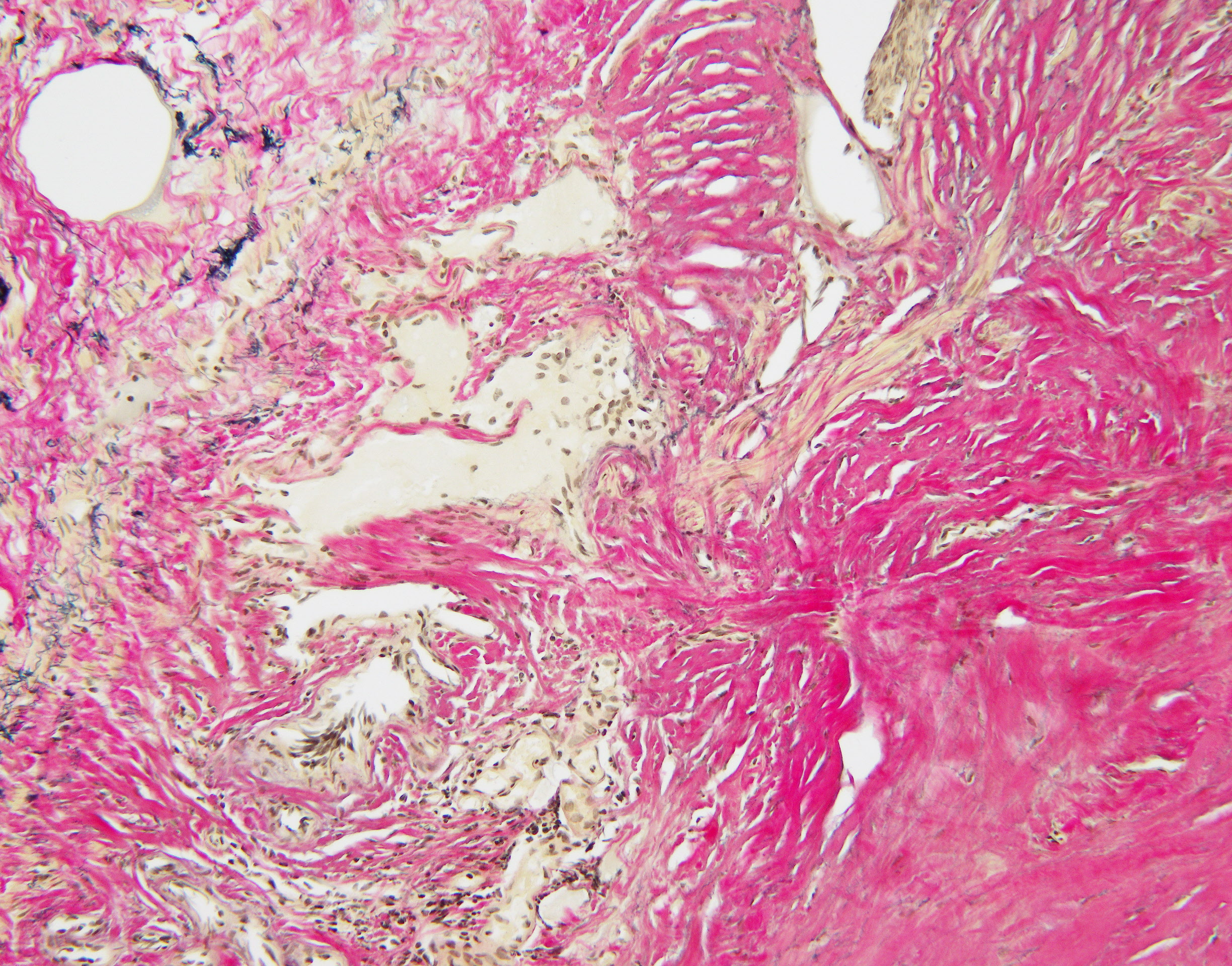

Supplement: Supplementary file 10 — Source data Fig. 5 [file 44321_2025_235_MOESM10_ESM.zip › Figure 5/Figure 5E.JPG]

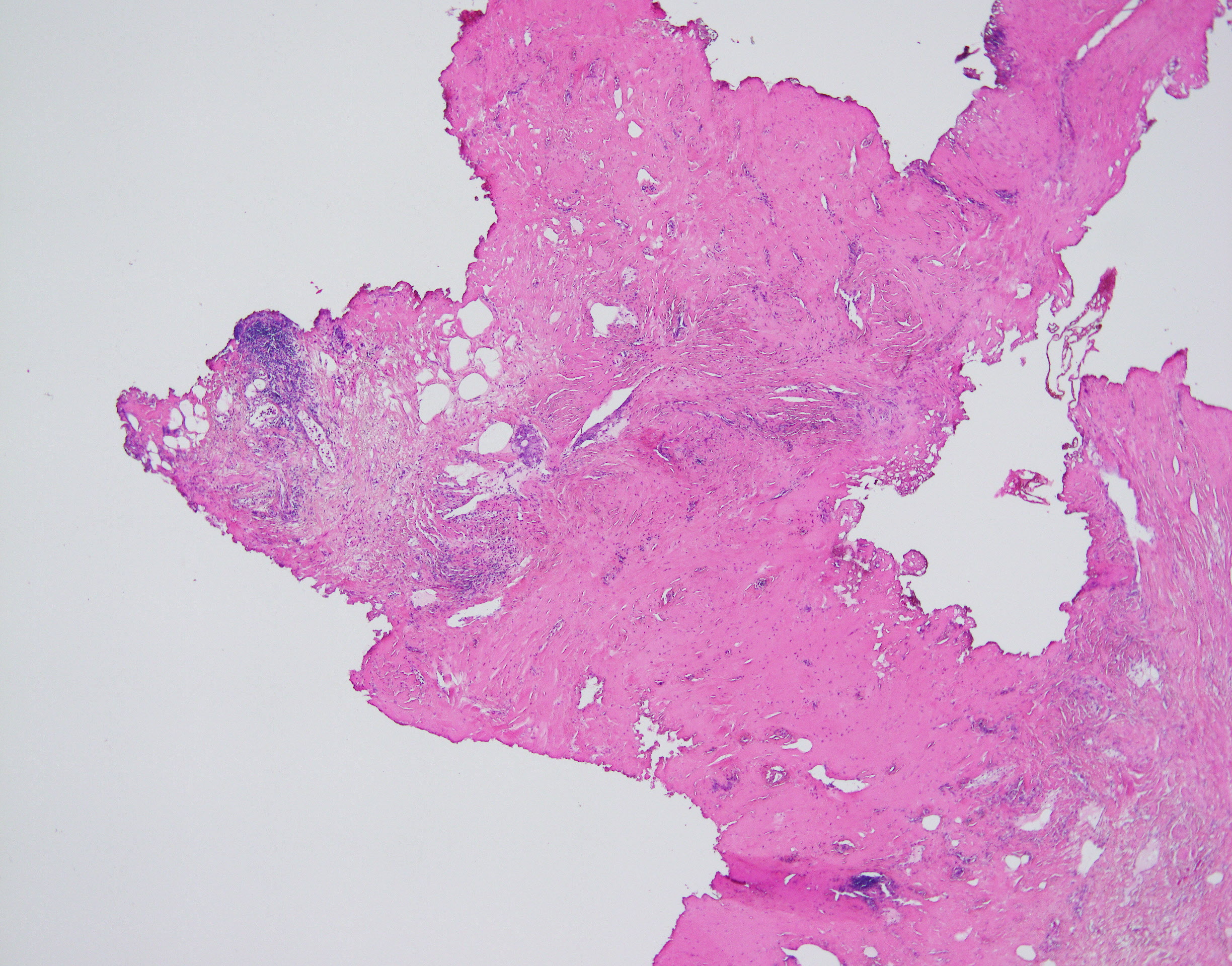

Supplement: Supplementary file 10 — Source data Fig. 5 [file 44321_2025_235_MOESM10_ESM.zip › Figure 5/Figure 5D.JPG]

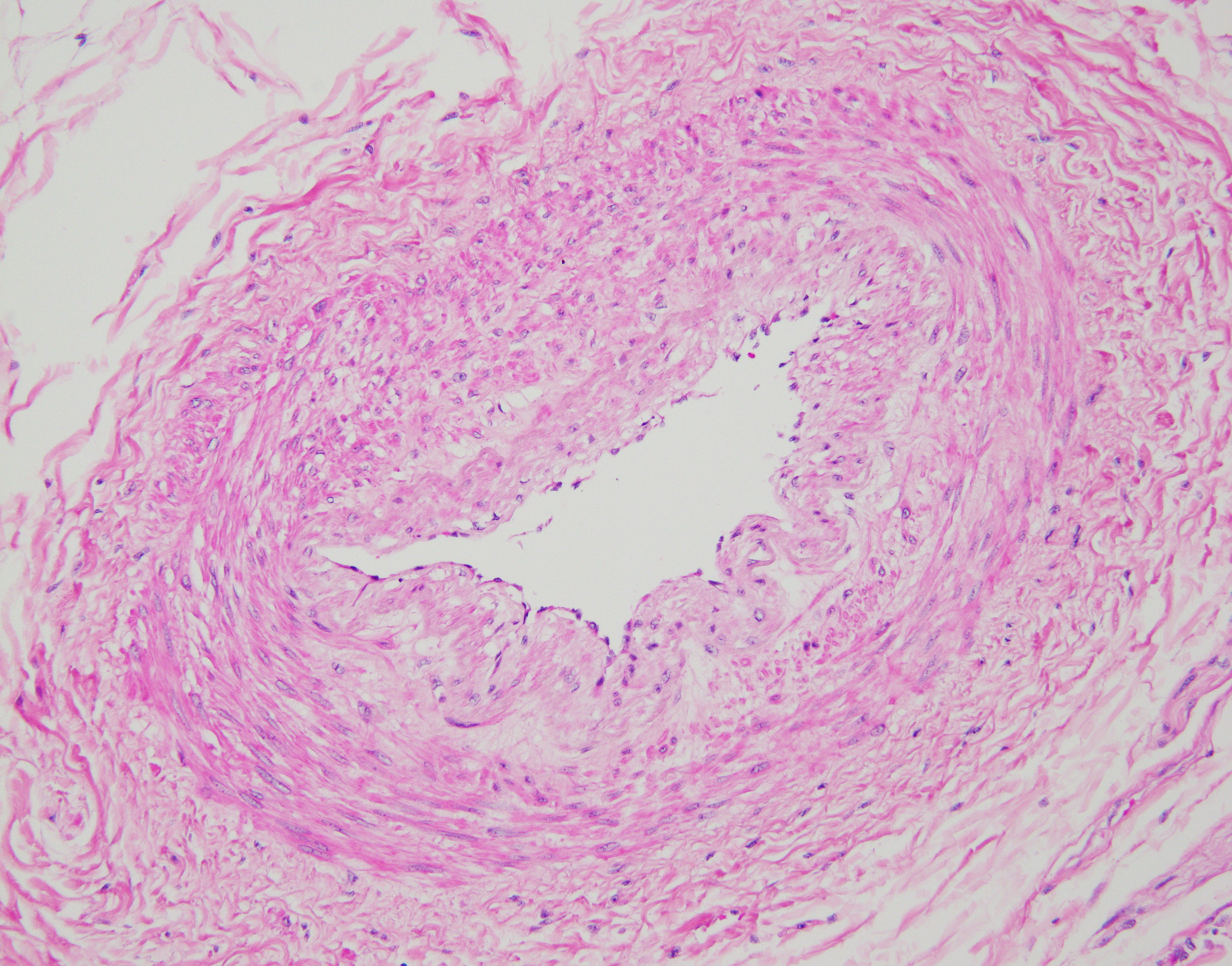

Supplement: Supplementary file 10 — Source data Fig. 5 [file 44321_2025_235_MOESM10_ESM.zip › Figure 5/Figure 5C.JPG]

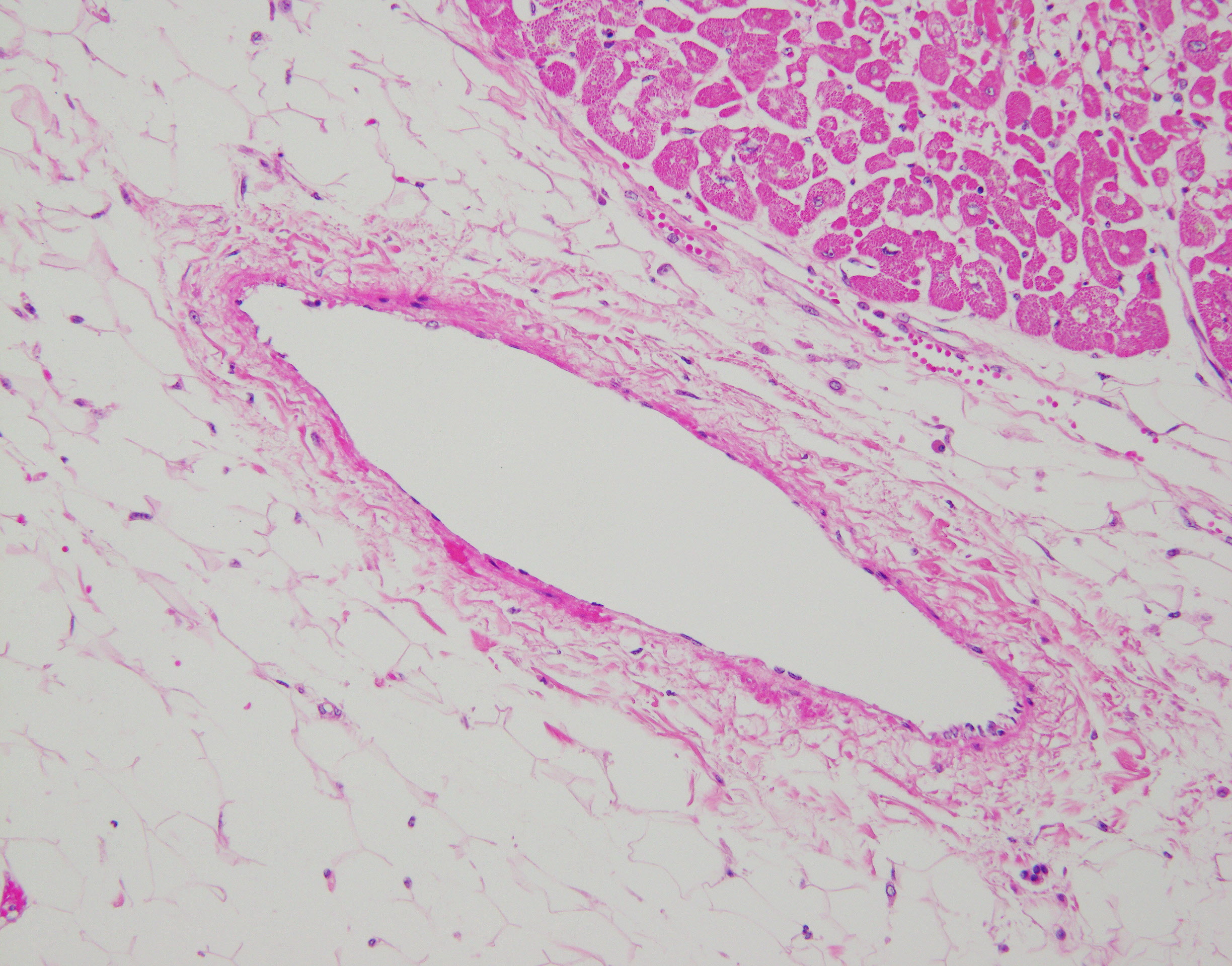

Supplement: Supplementary file 10 — Source data Fig. 5 [file 44321_2025_235_MOESM10_ESM.zip › Figure 5/Figure 5B.JPG]

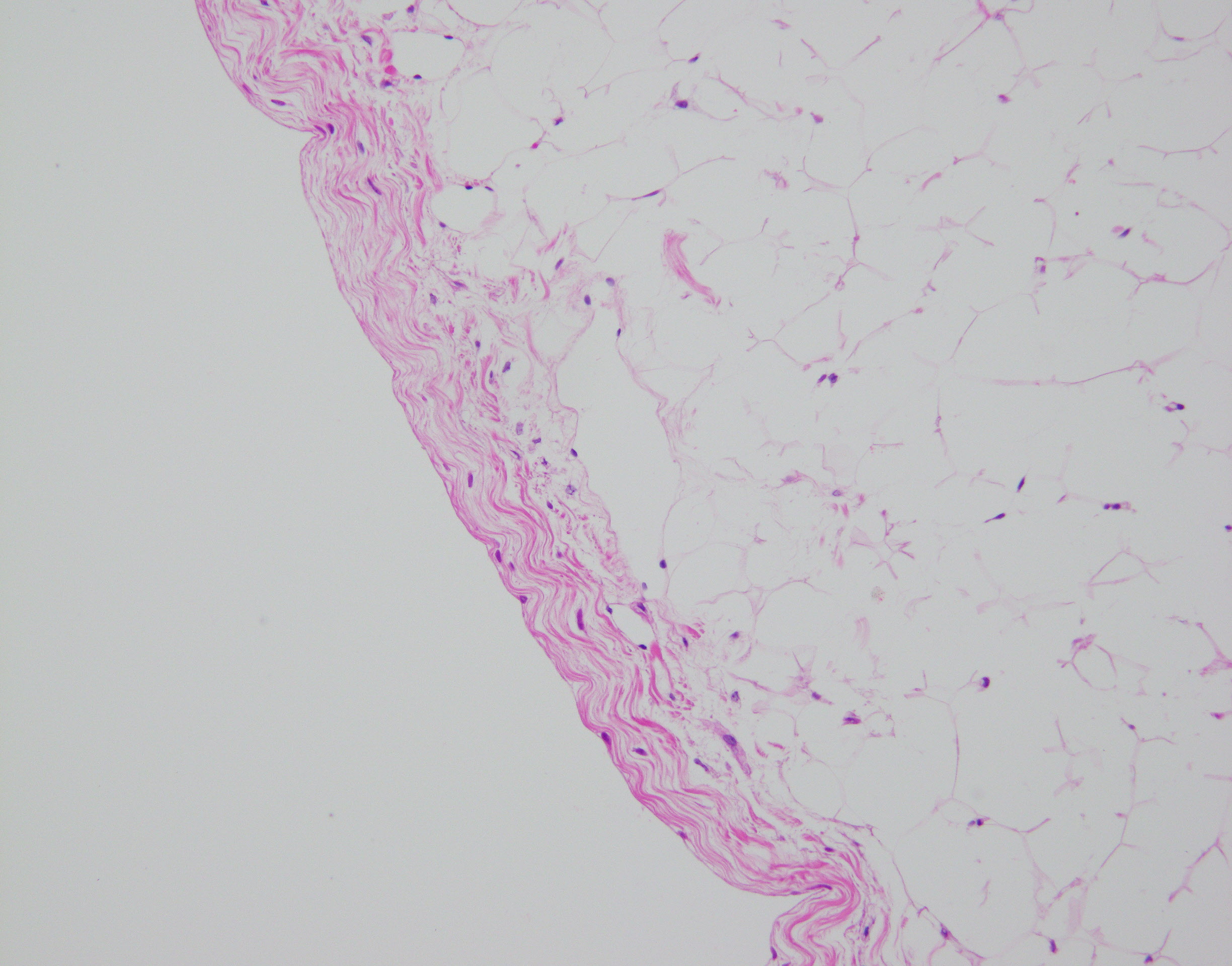

Supplement: Supplementary file 10 — Source data Fig. 5 [file 44321_2025_235_MOESM10_ESM.zip › Figure 5/Figure 5A.JPG]

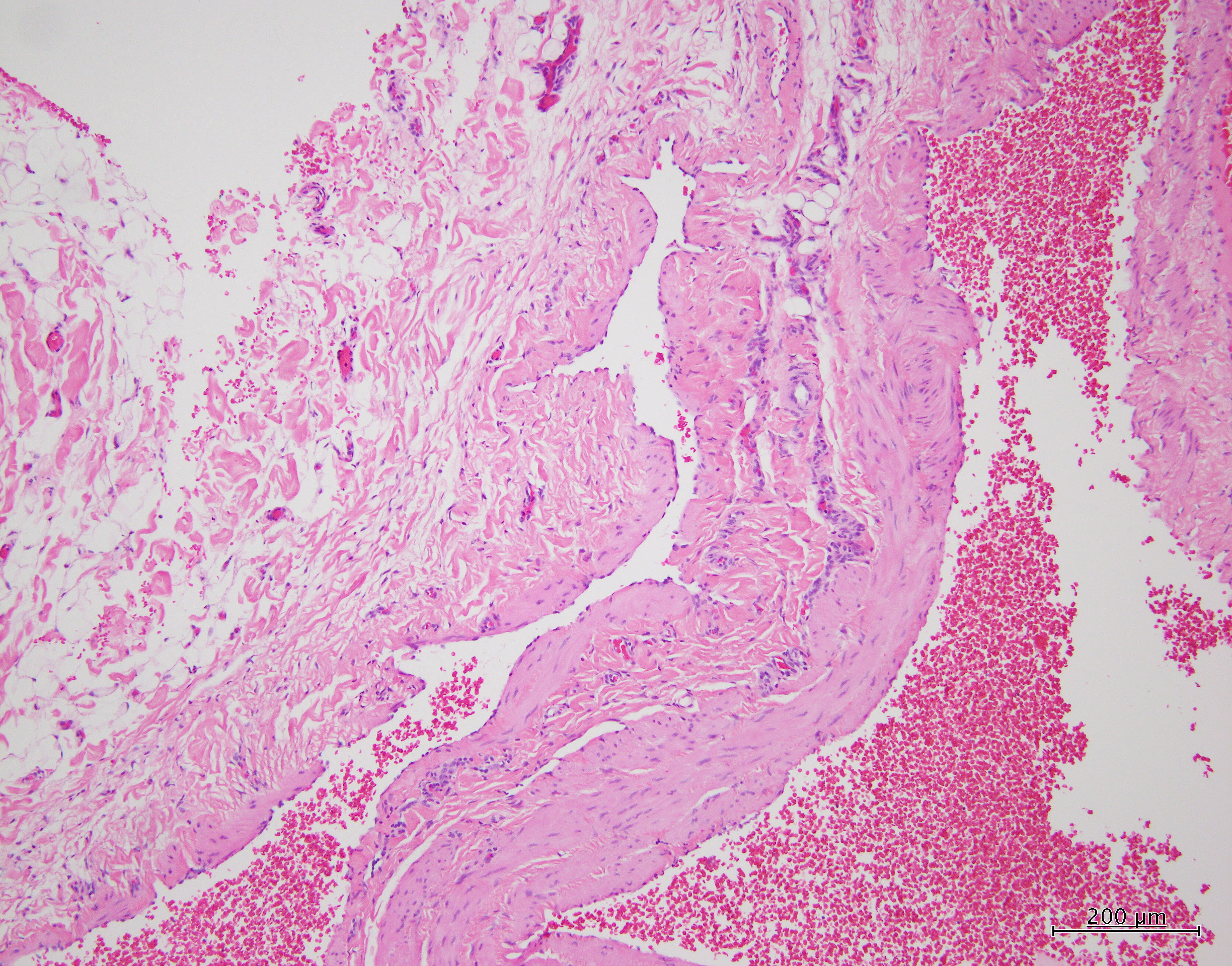

Supplement: Supplementary file 10 — Source data Fig. 5 [file 44321_2025_235_MOESM10_ESM.zip › Figure 5/Figure 5L.JPG]

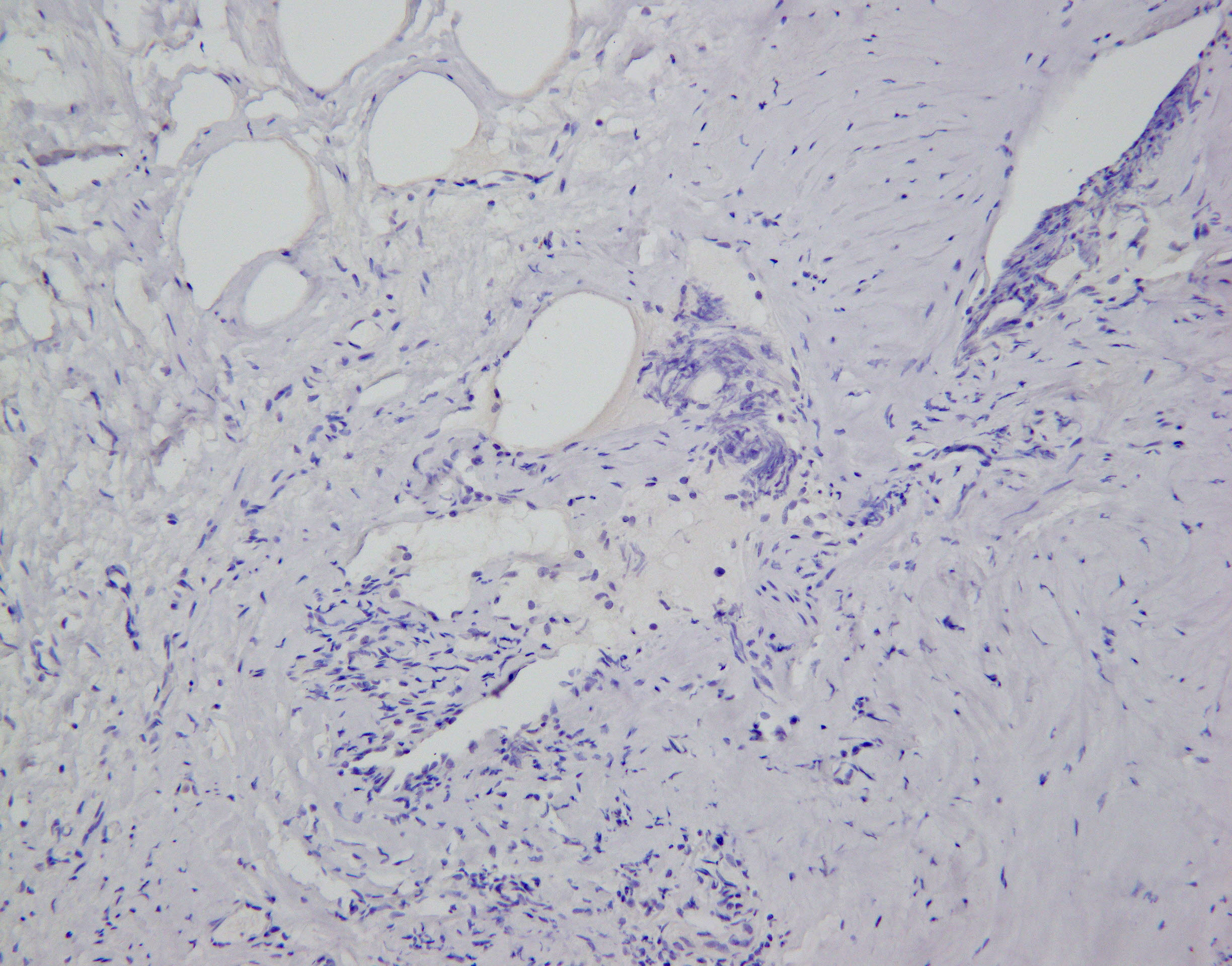

Supplement: Supplementary file 10 — Source data Fig. 5 [file 44321_2025_235_MOESM10_ESM.zip › Figure 5/Figure 5K.tif]

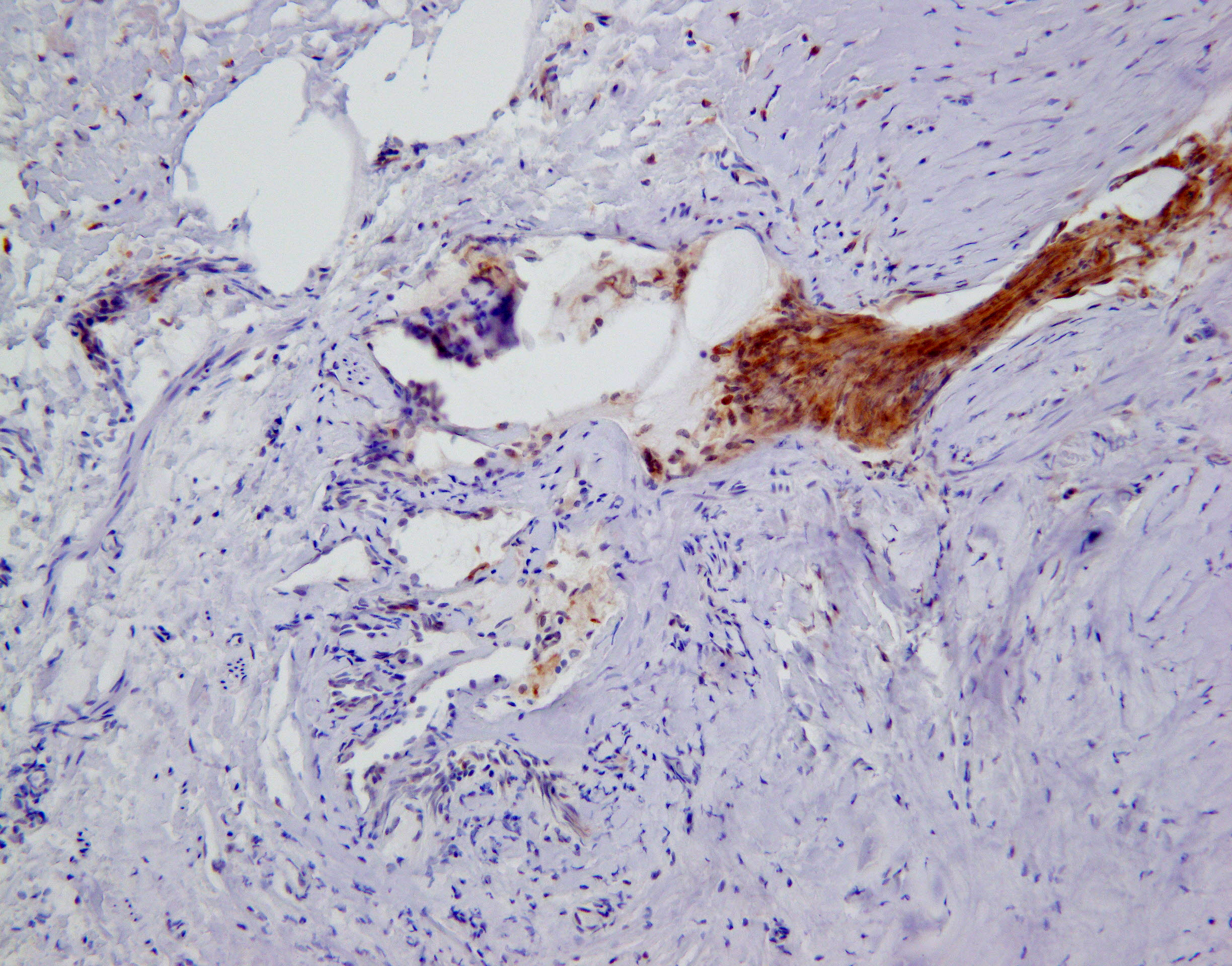

Supplement: Supplementary file 10 — Source data Fig. 5 [file 44321_2025_235_MOESM10_ESM.zip › Figure 5/Figure 5J.tif]

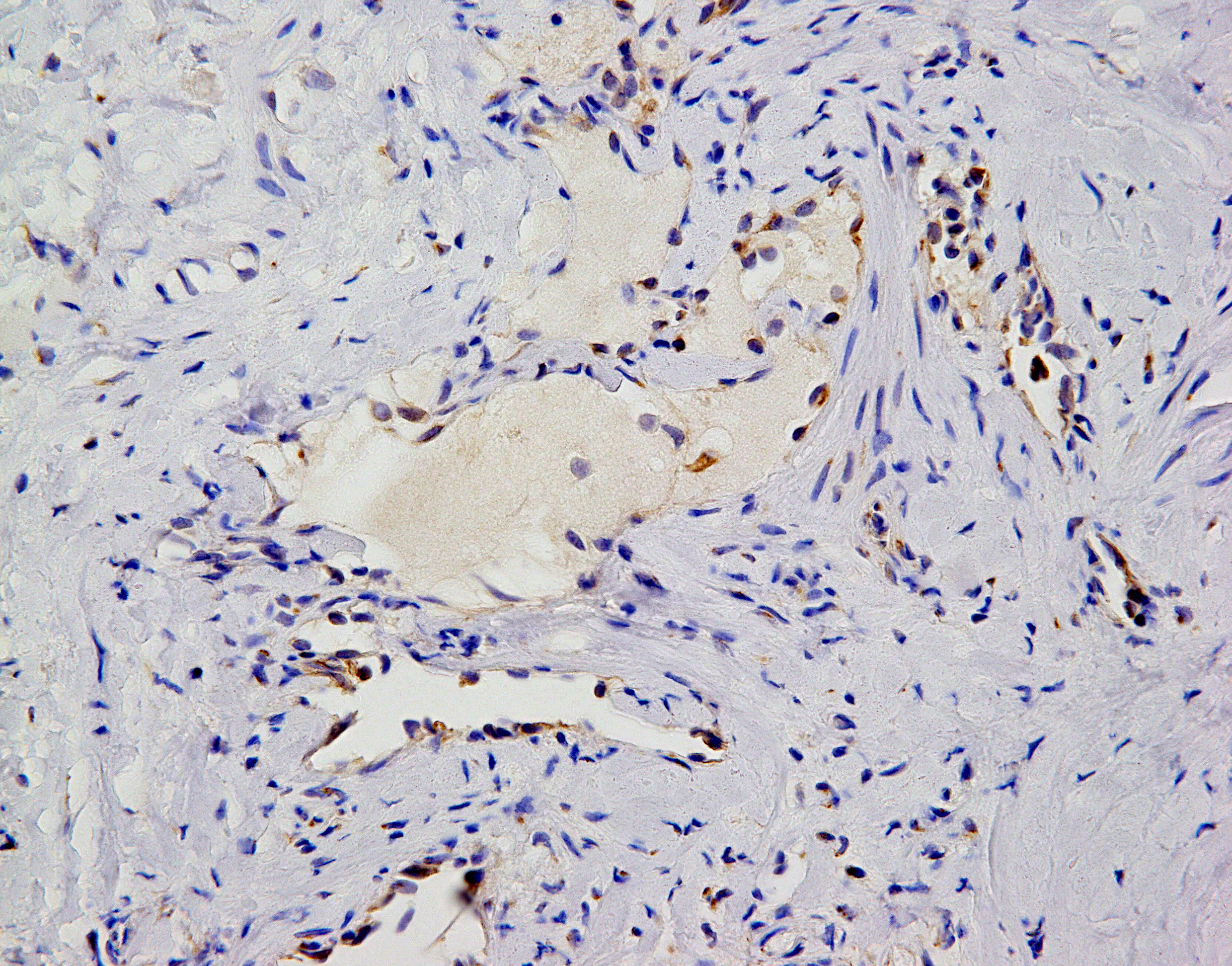

Supplement: Supplementary file 10 — Source data Fig. 5 [file 44321_2025_235_MOESM10_ESM.zip › Figure 5/Figure 5H.tif]

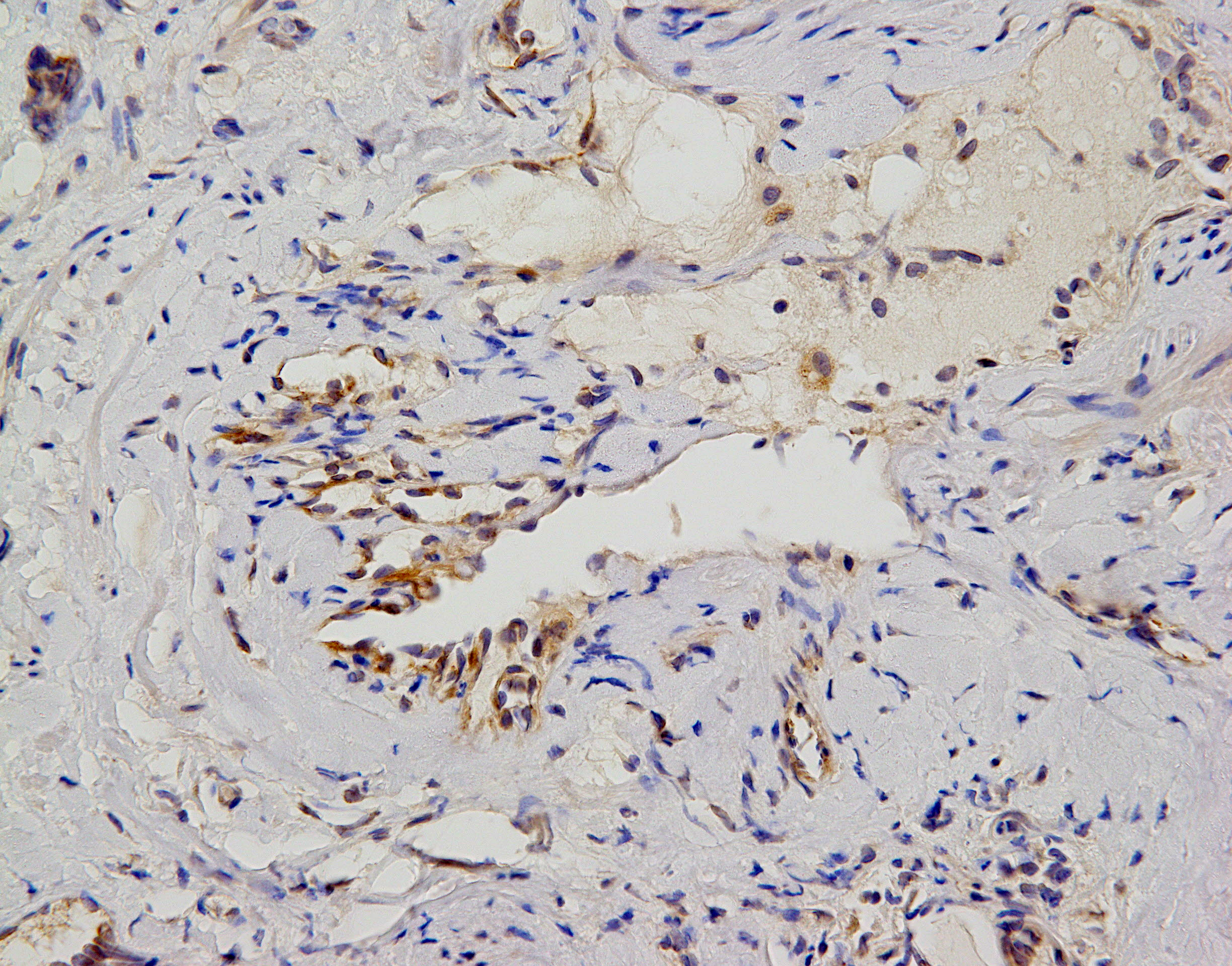

Supplement: Supplementary file 10 — Source data Fig. 5 [file 44321_2025_235_MOESM10_ESM.zip › Figure 5/Figure 5I.tif]

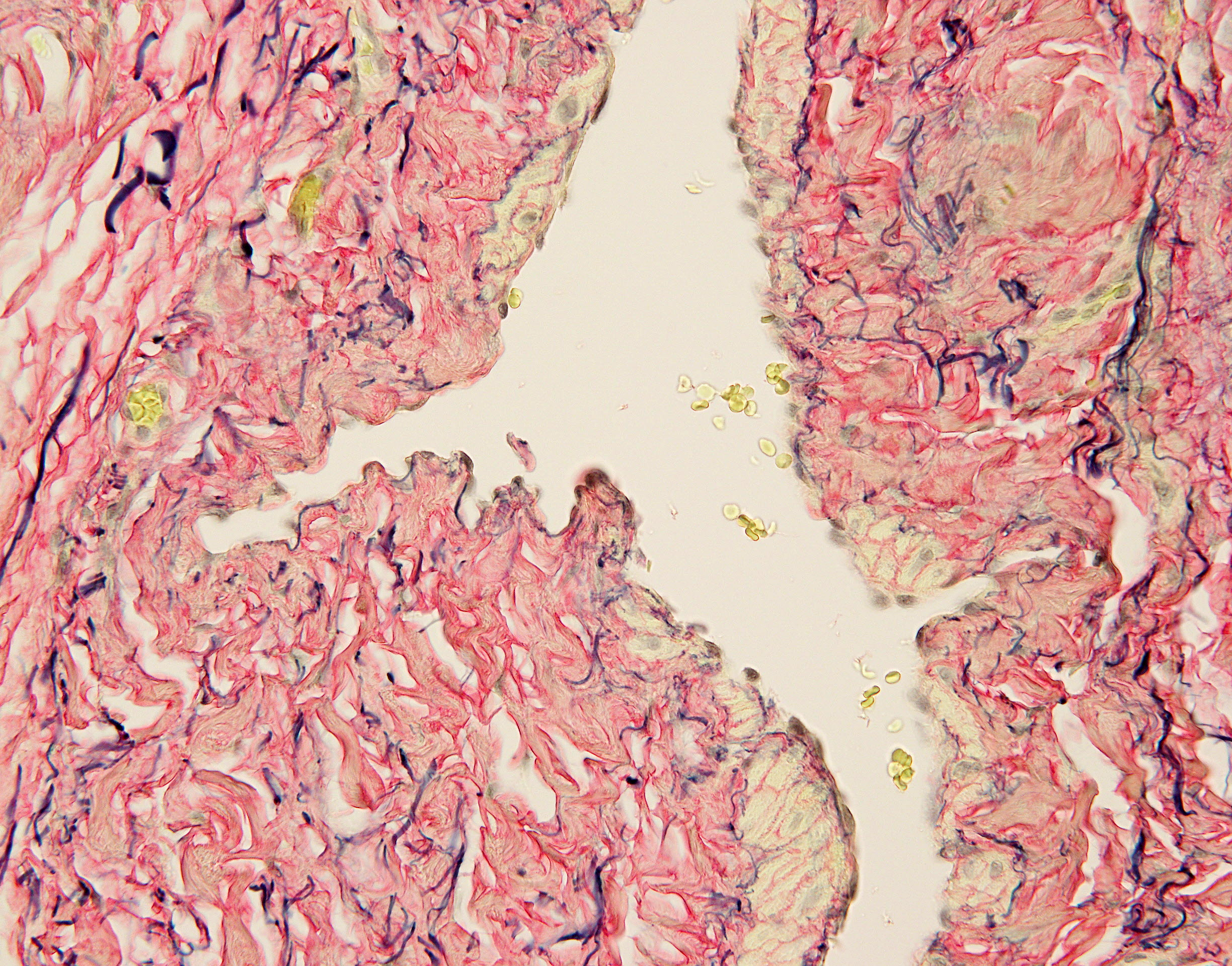

Supplement: Supplementary file 10 — Source data Fig. 5 [file 44321_2025_235_MOESM10_ESM.zip › Figure 5/Figure 5M.tif]

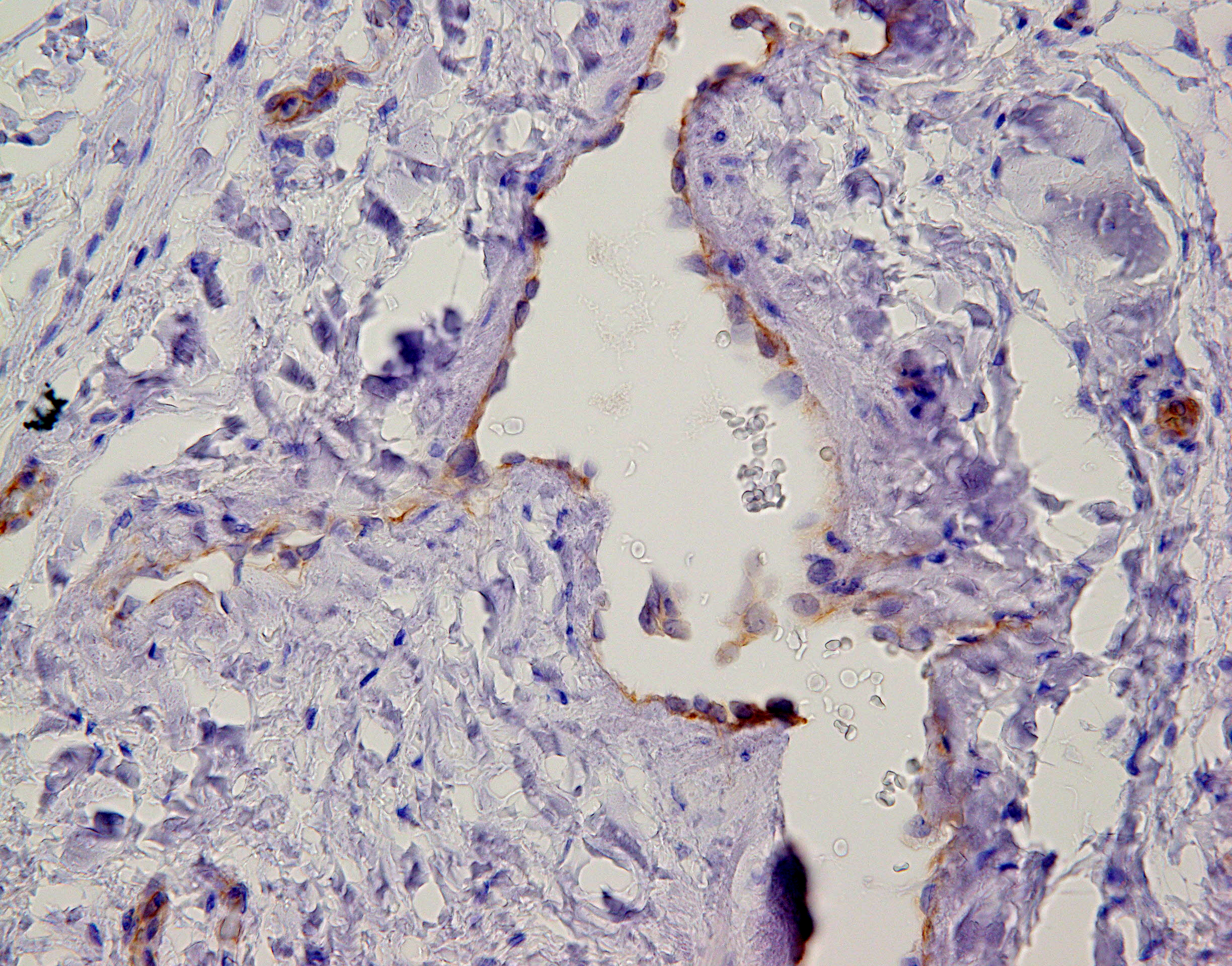

Supplement: Supplementary file 10 — Source data Fig. 5 [file 44321_2025_235_MOESM10_ESM.zip › Figure 5/Figure 5N.tif]

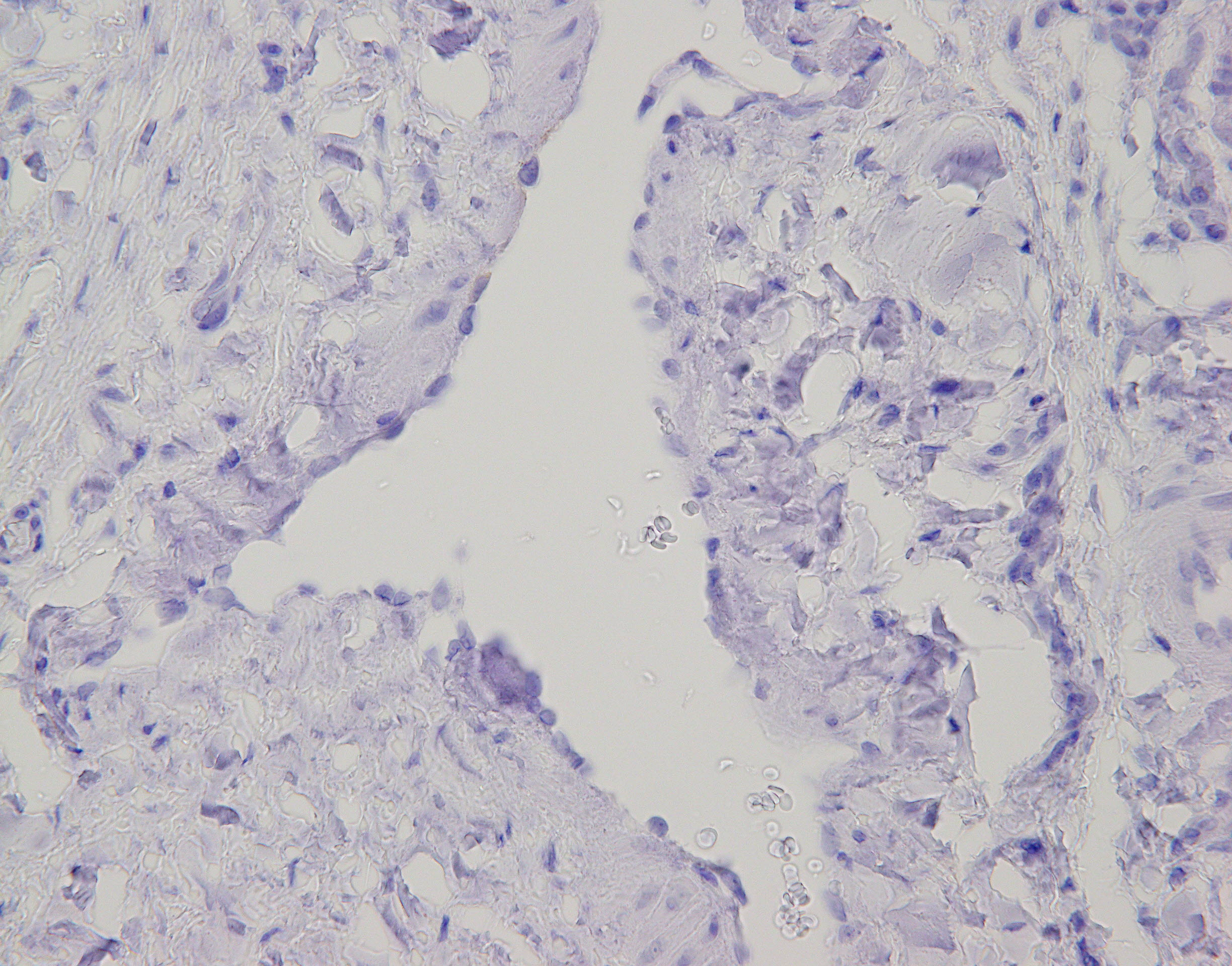

Supplement: Supplementary file 10 — Source data Fig. 5 [file 44321_2025_235_MOESM10_ESM.zip › Figure 5/Figure 5O.tif]

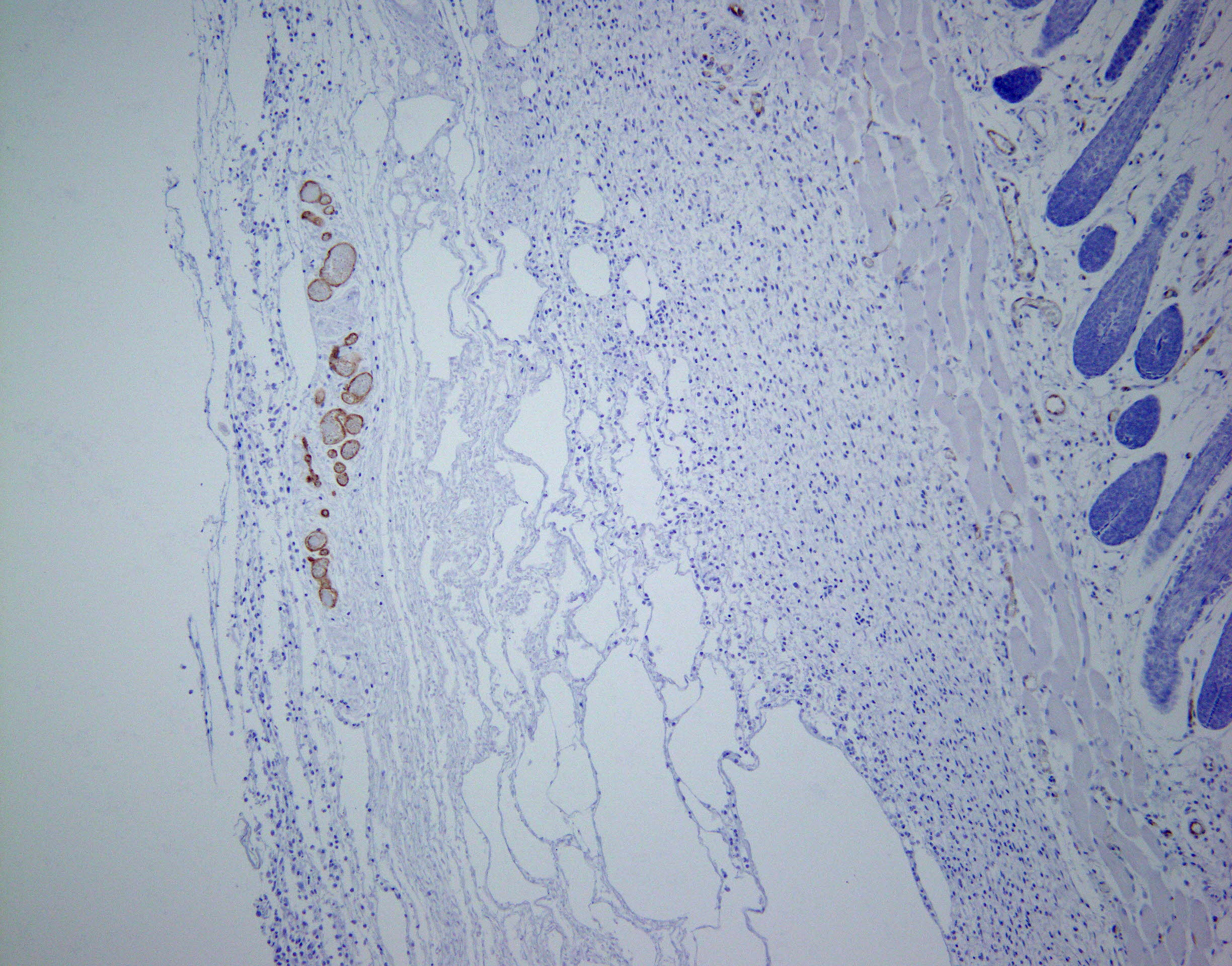

Supplement: Supplementary file 11 — Source data Fig. 6 [file 44321_2025_235_MOESM11_ESM.zip › Figure 6/Figure 6K (New Figure 6P).tif]

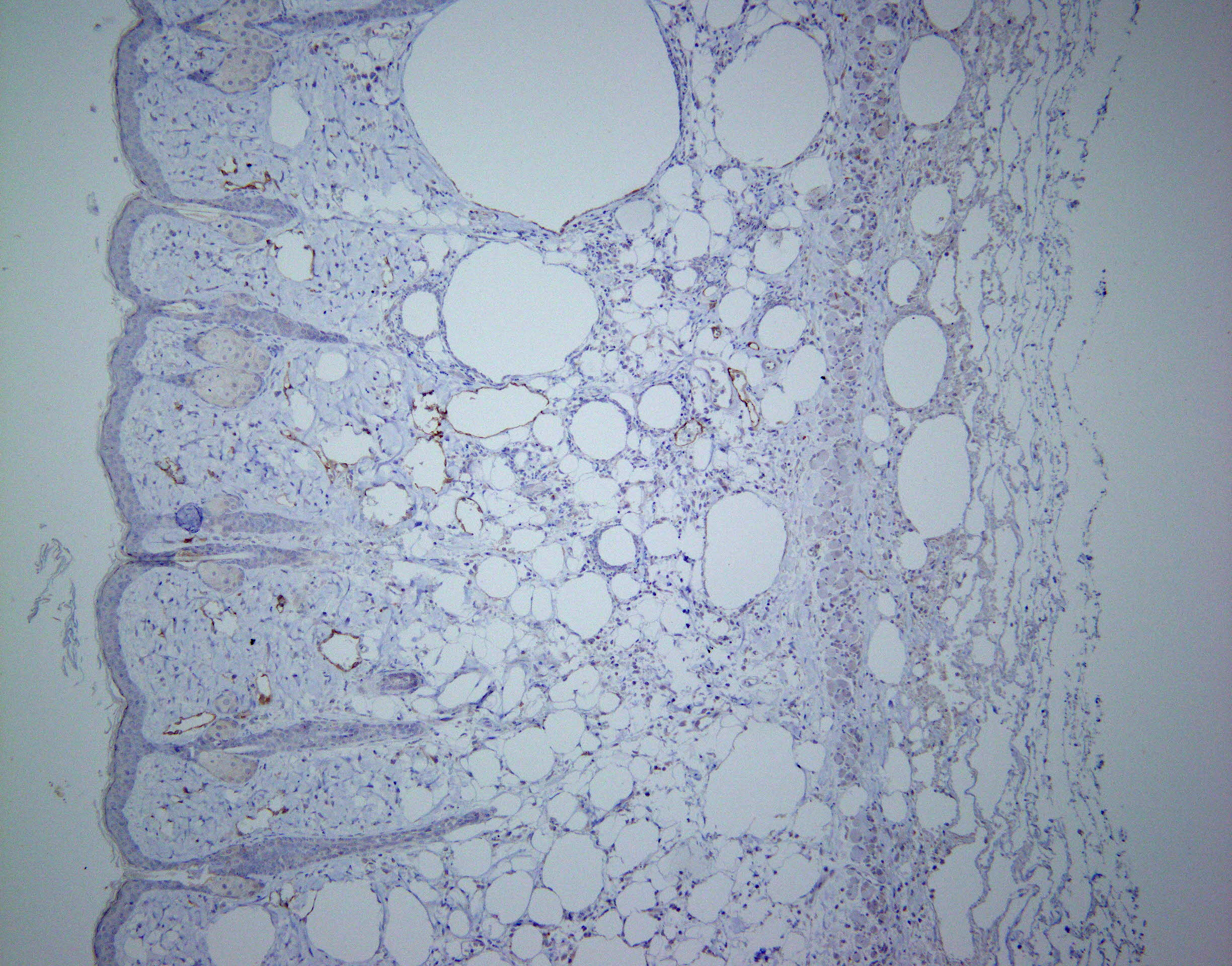

Supplement: Supplementary file 11 — Source data Fig. 6 [file 44321_2025_235_MOESM11_ESM.zip › Figure 6/Figure 6S (New Figure 6Z).tif]

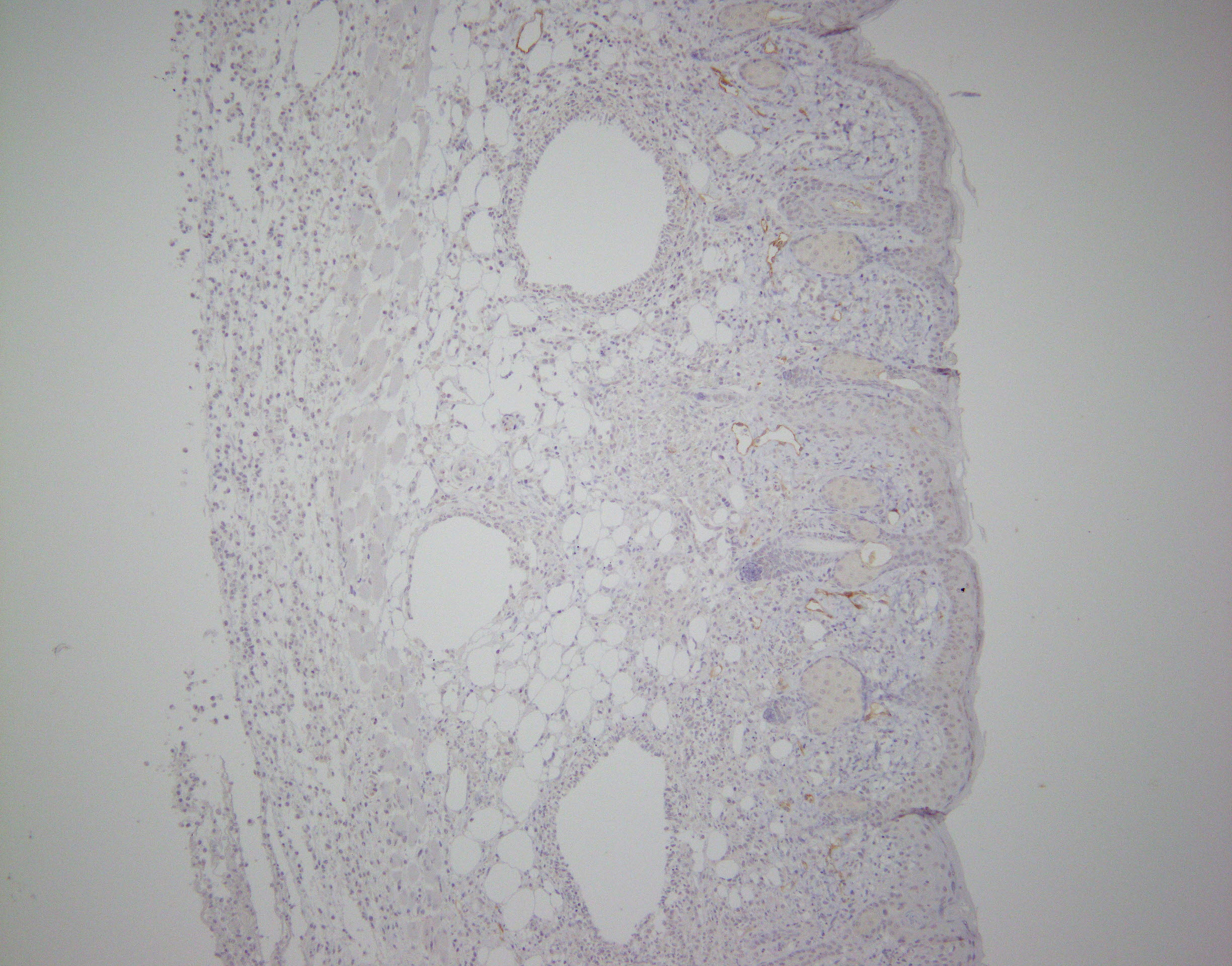

Supplement: Supplementary file 11 — Source data Fig. 6 [file 44321_2025_235_MOESM11_ESM.zip › Figure 6/Figure 6Q (New Figure 6V).tif]

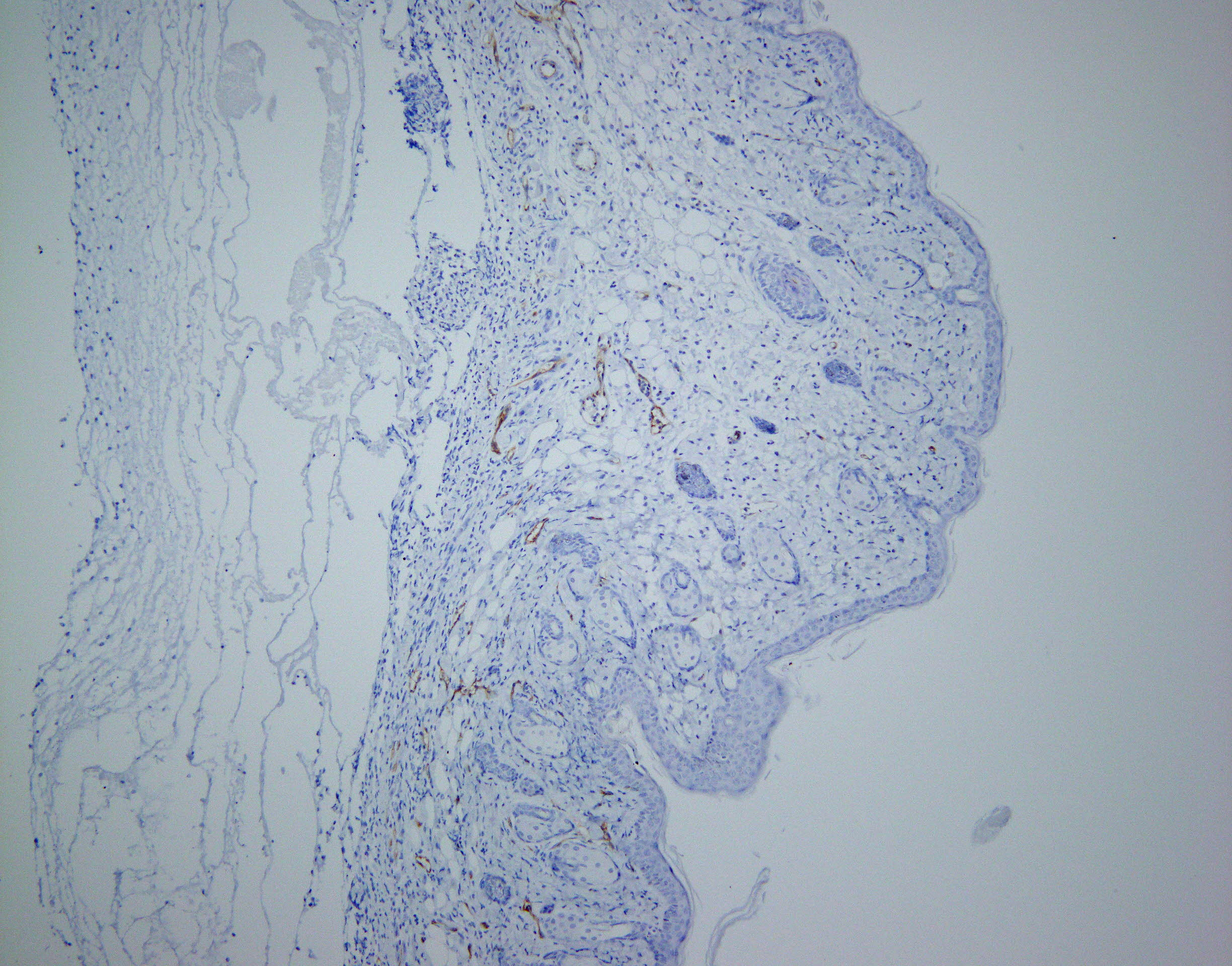

Supplement: Supplementary file 11 — Source data Fig. 6 [file 44321_2025_235_MOESM11_ESM.zip › Figure 6/Figure 6J (New Figure 6N).tif]

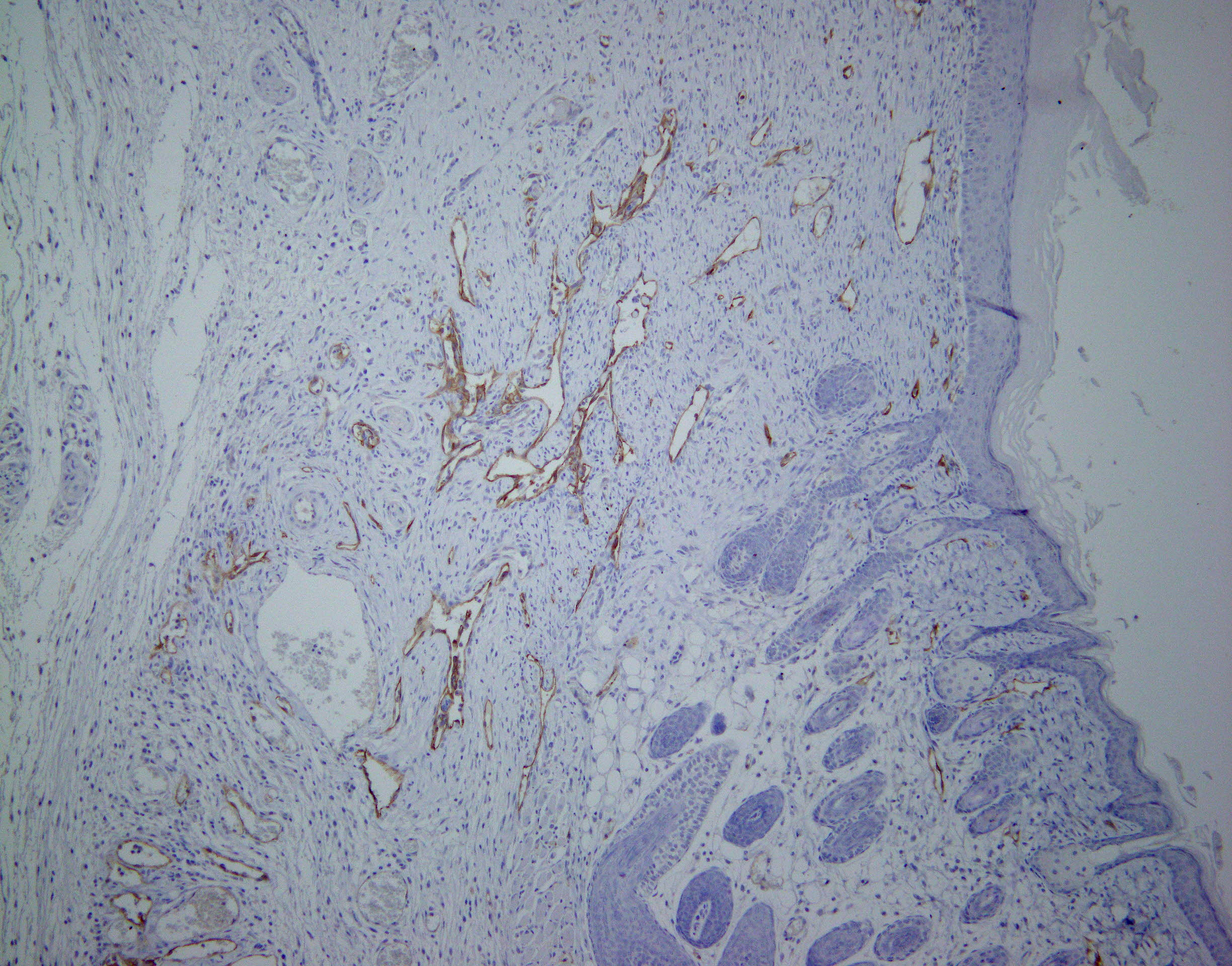

Supplement: Supplementary file 11 — Source data Fig. 6 [file 44321_2025_235_MOESM11_ESM.zip › Figure 6/Figure 6O (New Figure 6W).tif]

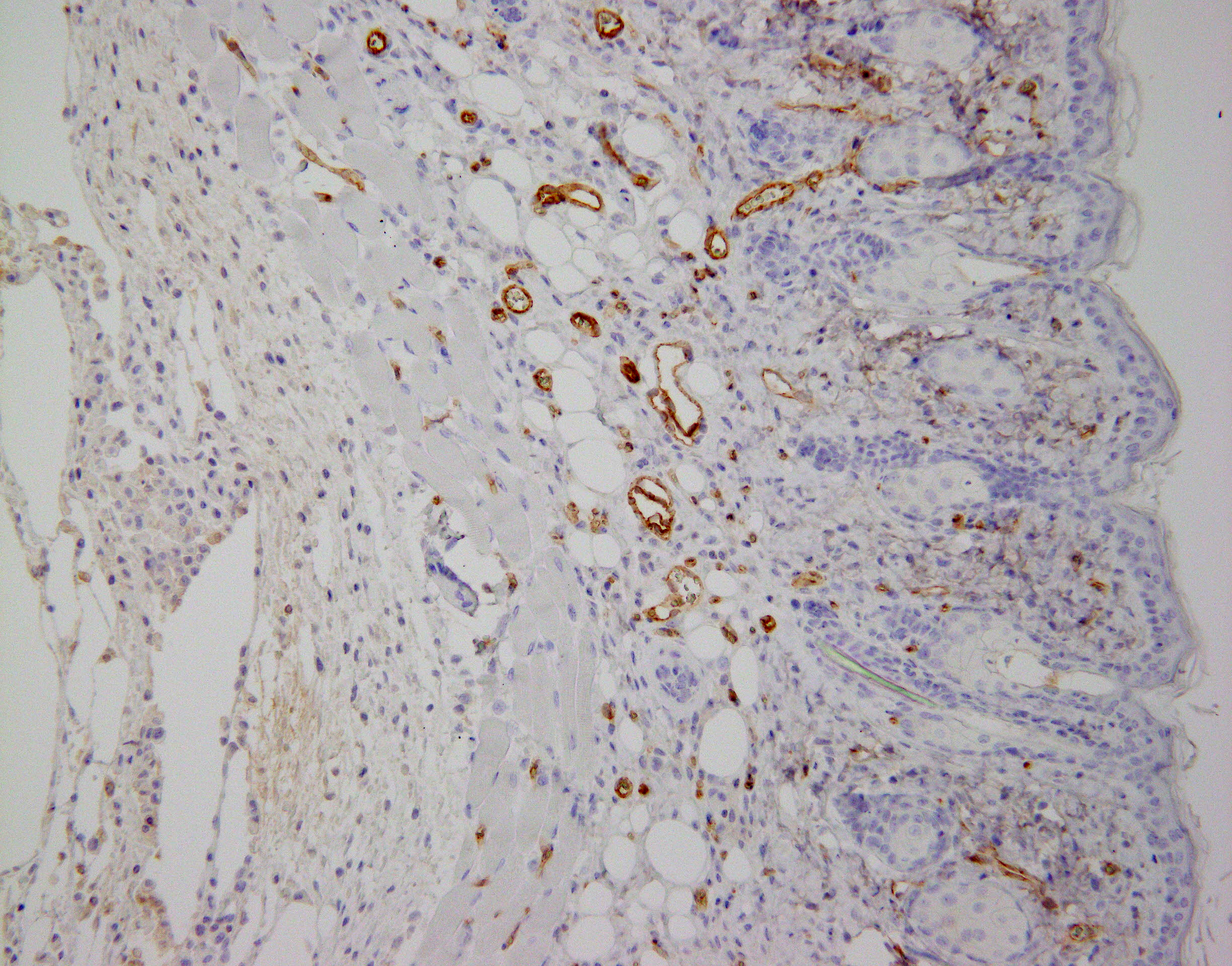

Supplement: Supplementary file 11 — Source data Fig. 6 [file 44321_2025_235_MOESM11_ESM.zip › Figure 6/Figure 6C (New Figure 6E).tif]

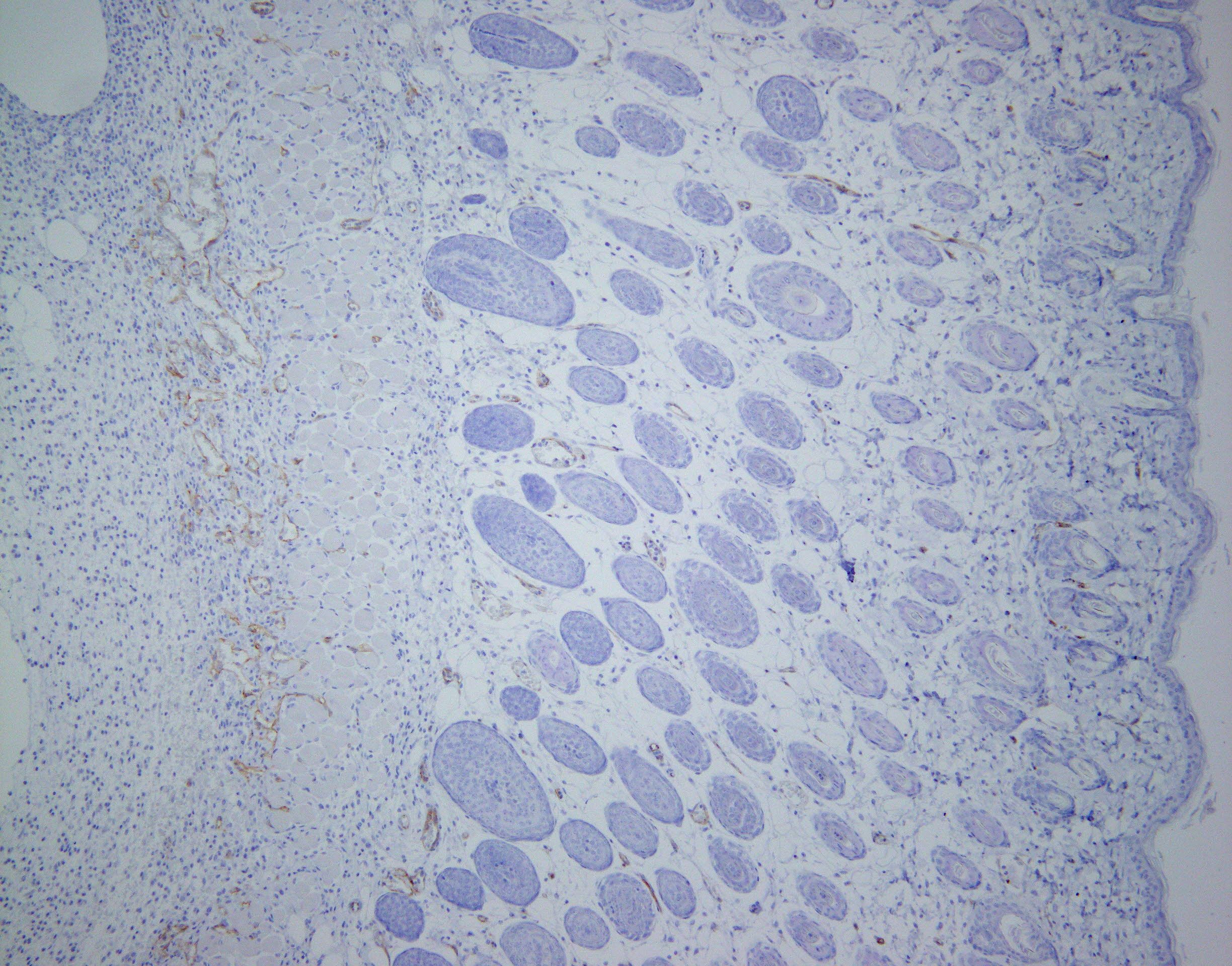

Supplement: Supplementary file 11 — Source data Fig. 6 [file 44321_2025_235_MOESM11_ESM.zip › Figure 6/Figure 6H (New Figure 6O).tif]

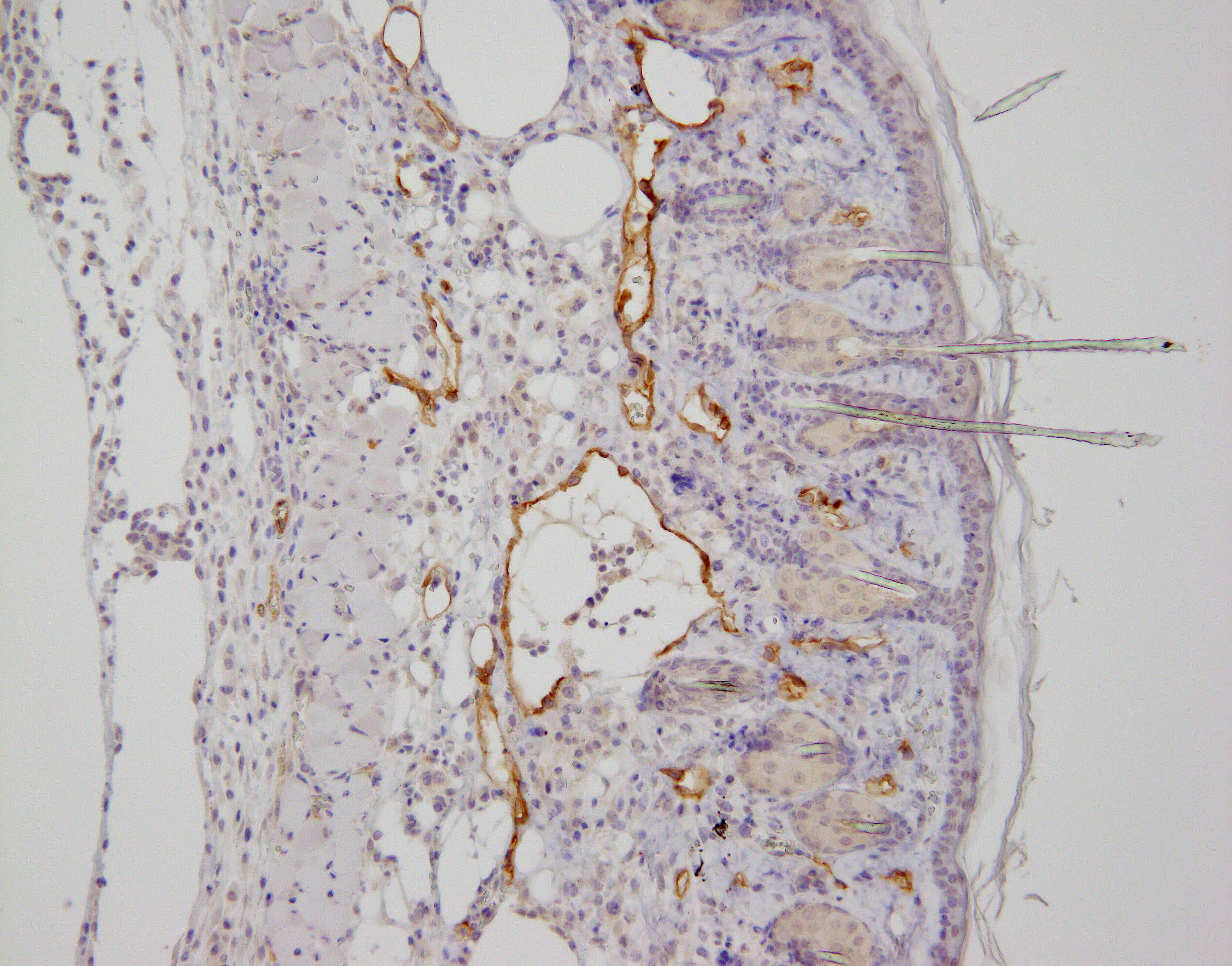

Supplement: Supplementary file 11 — Source data Fig. 6 [file 44321_2025_235_MOESM11_ESM.zip › Figure 6/Figure 6C (New Figure 6F).tif]

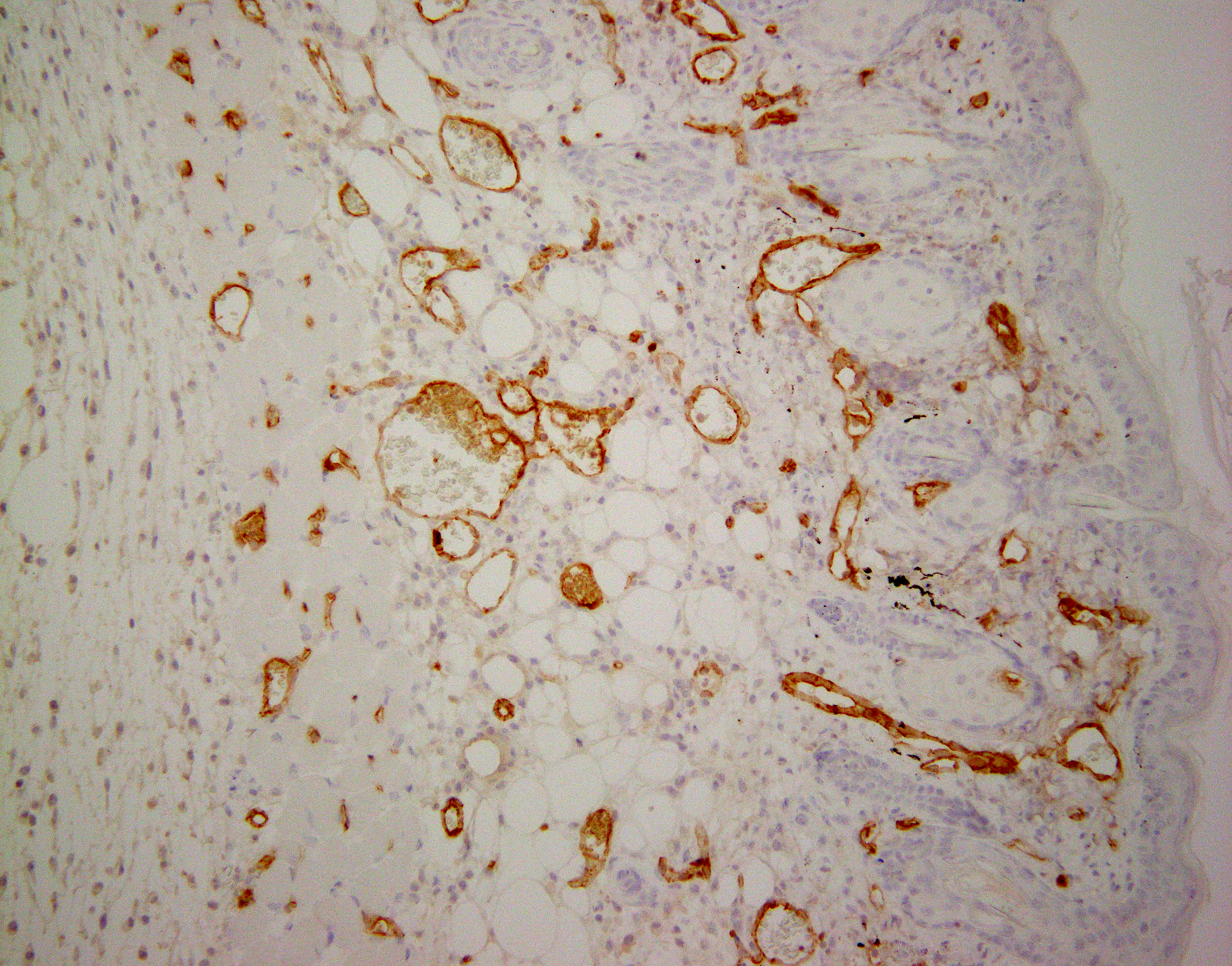

Supplement: Supplementary file 11 — Source data Fig. 6 [file 44321_2025_235_MOESM11_ESM.zip › Figure 6/Figure 6F (New Figure 6K).tif]

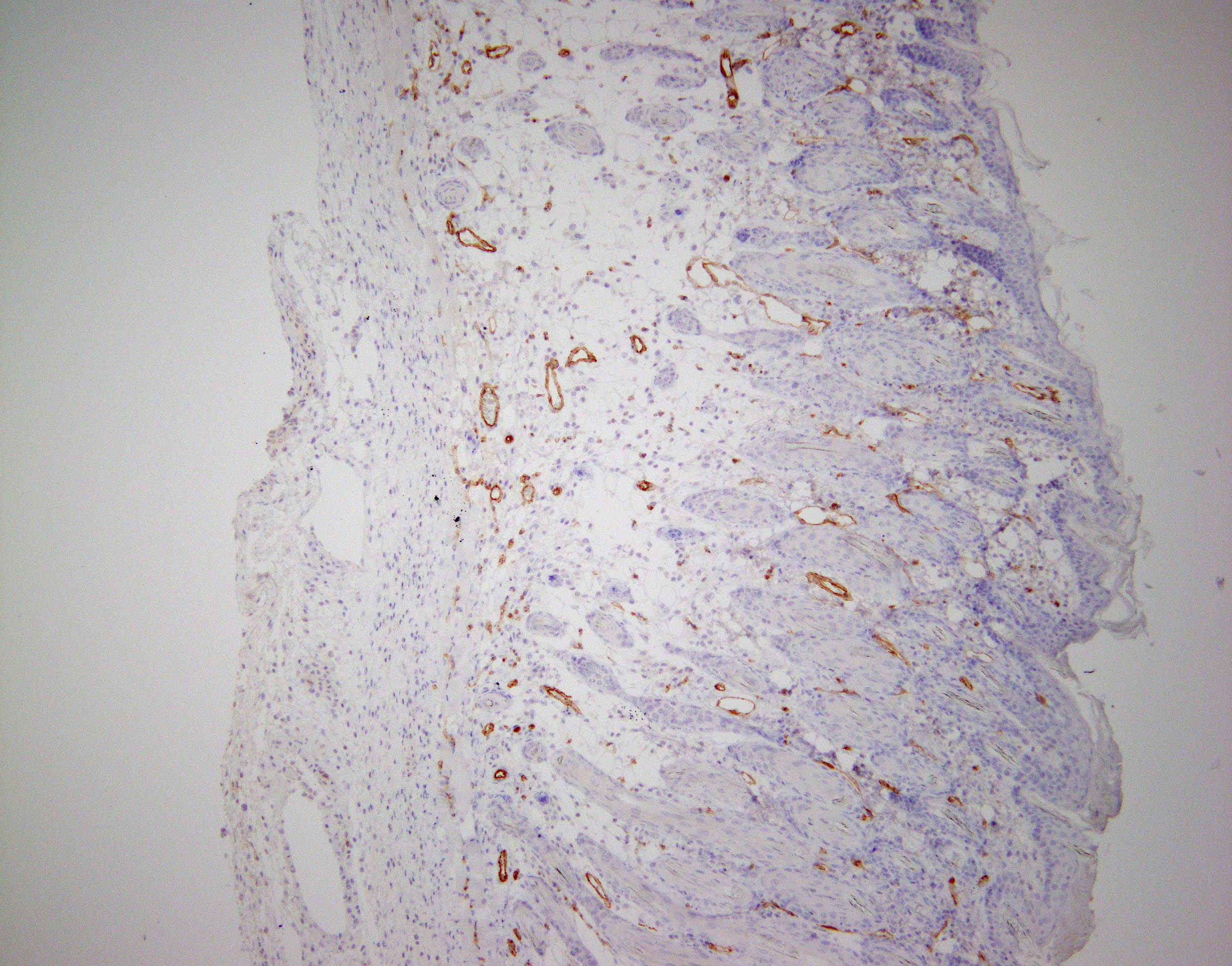

Supplement: Supplementary file 11 — Source data Fig. 6 [file 44321_2025_235_MOESM11_ESM.zip › Figure 6/Figure 6I (New Figure 6L).tif]

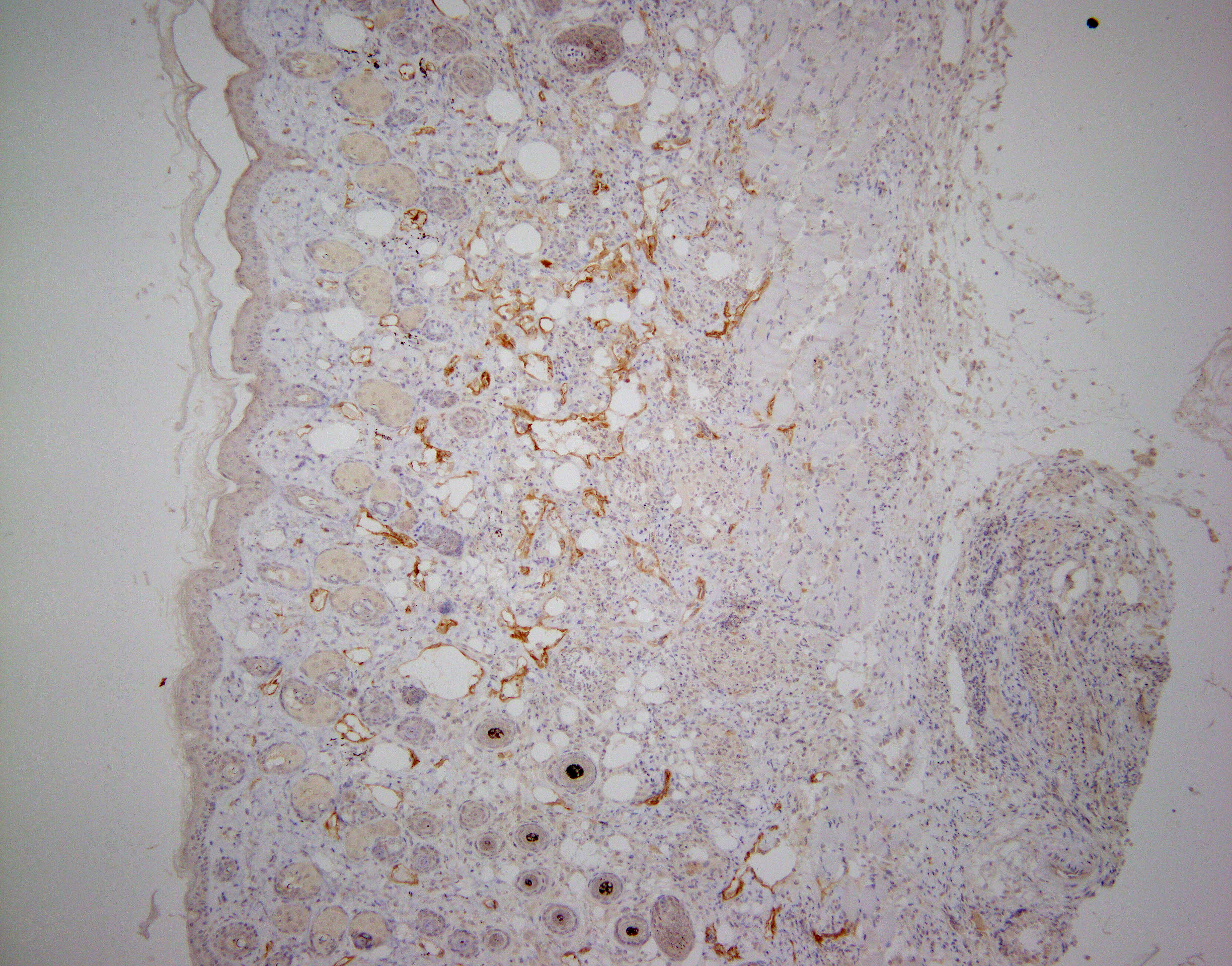

Supplement: Supplementary file 11 — Source data Fig. 6 [file 44321_2025_235_MOESM11_ESM.zip › Figure 6/Figure 6N (New Figure 6U).tif]

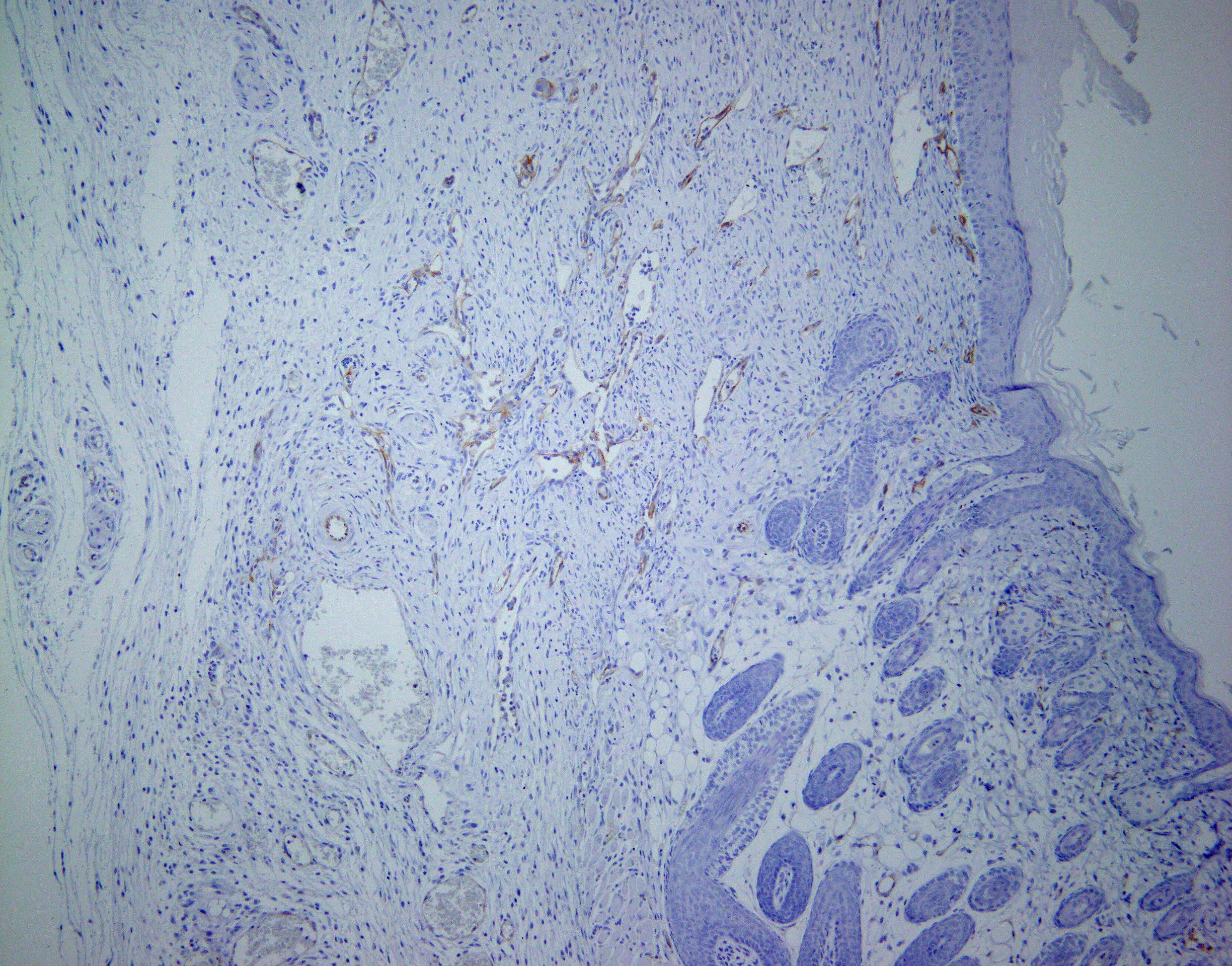

Supplement: Supplementary file 11 — Source data Fig. 6 [file 44321_2025_235_MOESM11_ESM.zip › Figure 6/Figure 6G (New Figure 6M).tif]

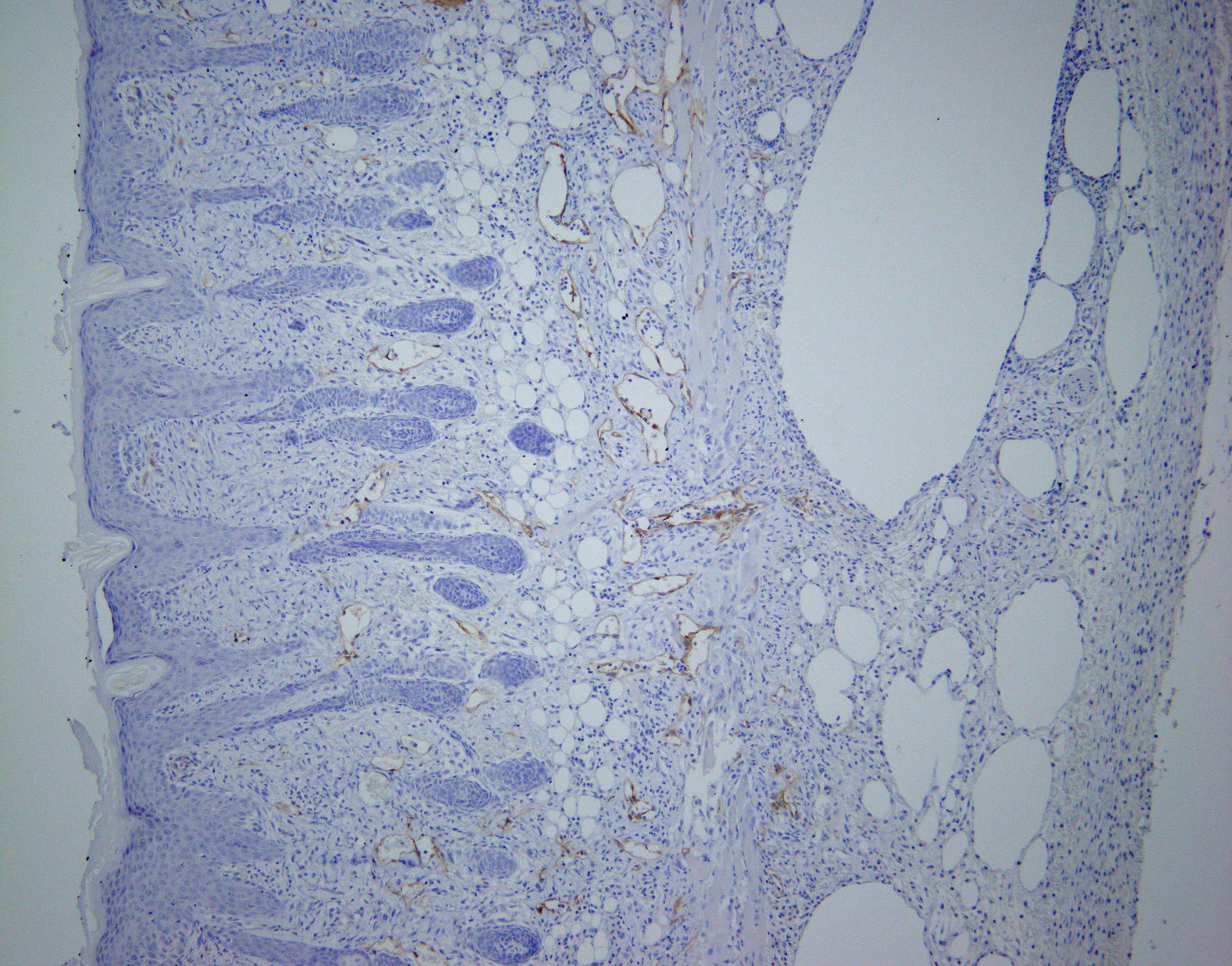

Supplement: Supplementary file 11 — Source data Fig. 6 [file 44321_2025_235_MOESM11_ESM.zip › Figure 6/Figure 6P (New Figure 6Y).tif]

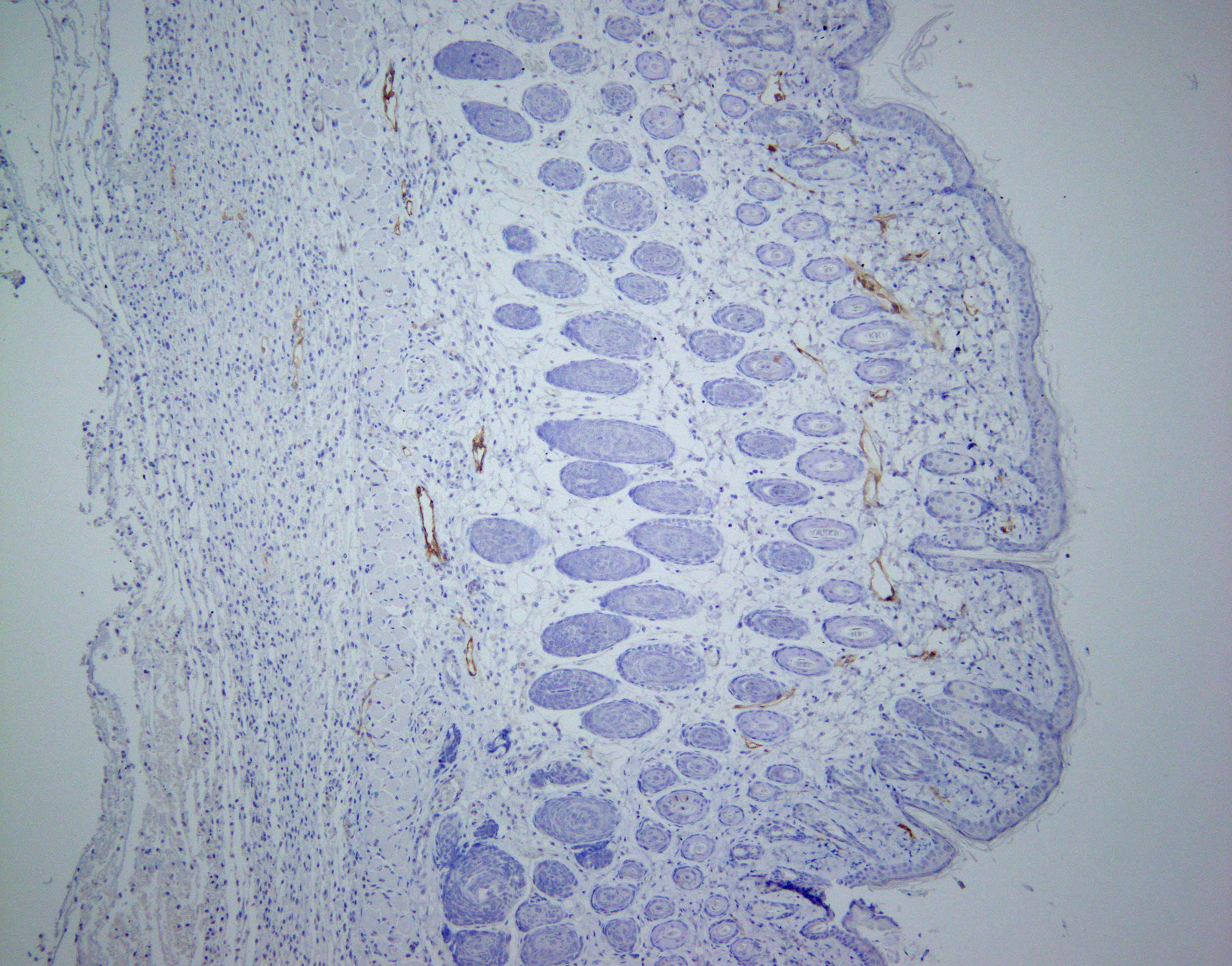

Supplement: Supplementary file 11 — Source data Fig. 6 [file 44321_2025_235_MOESM11_ESM.zip › Figure 6/Figure 6R (New Figure 6X).tif]

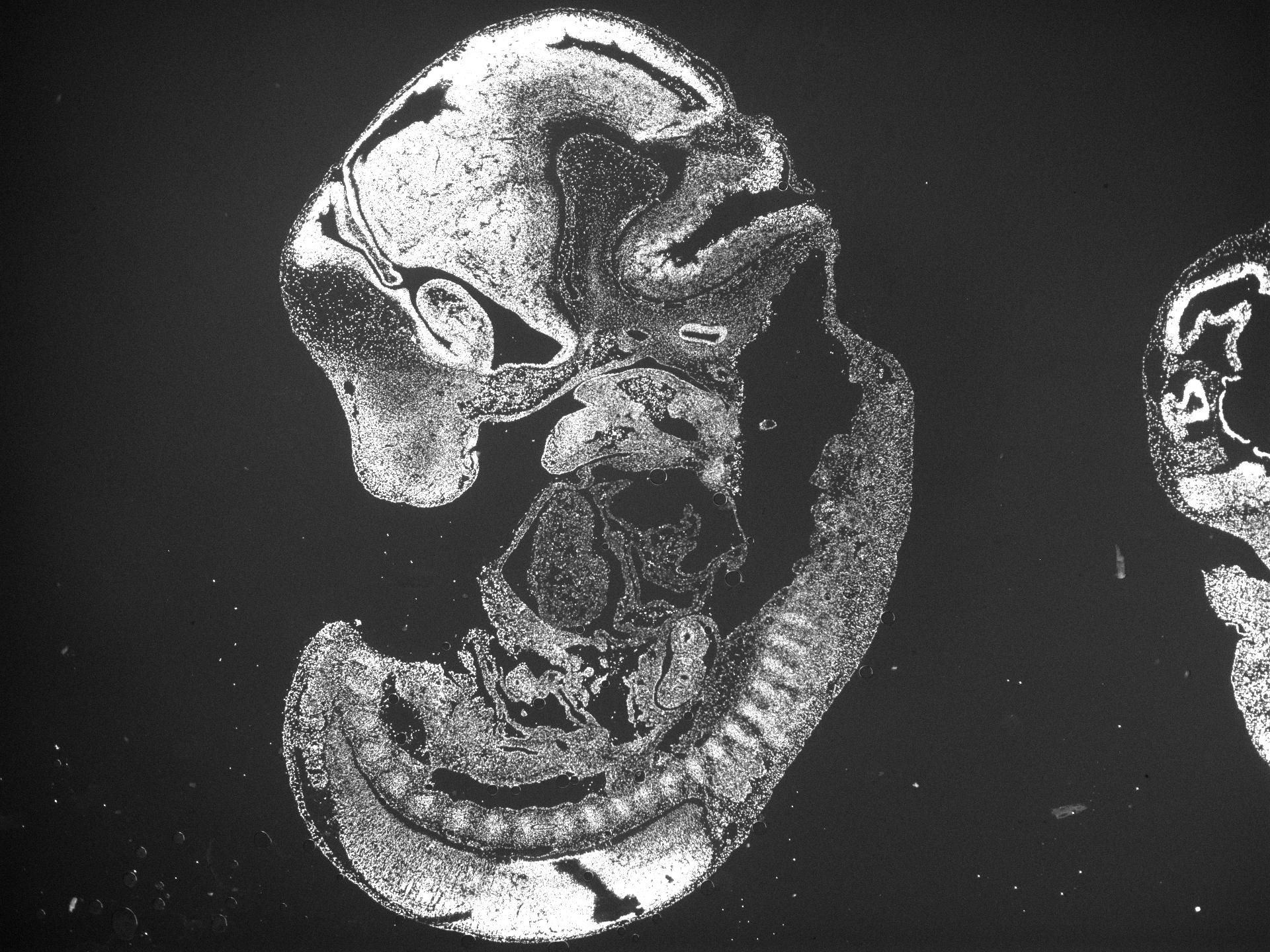

Supplement: Supplementary file 12 — Source data Fig. 1 [file 44321_2025_235_MOESM12_ESM.zip › Figure 1/Figure 1B-2.tif]

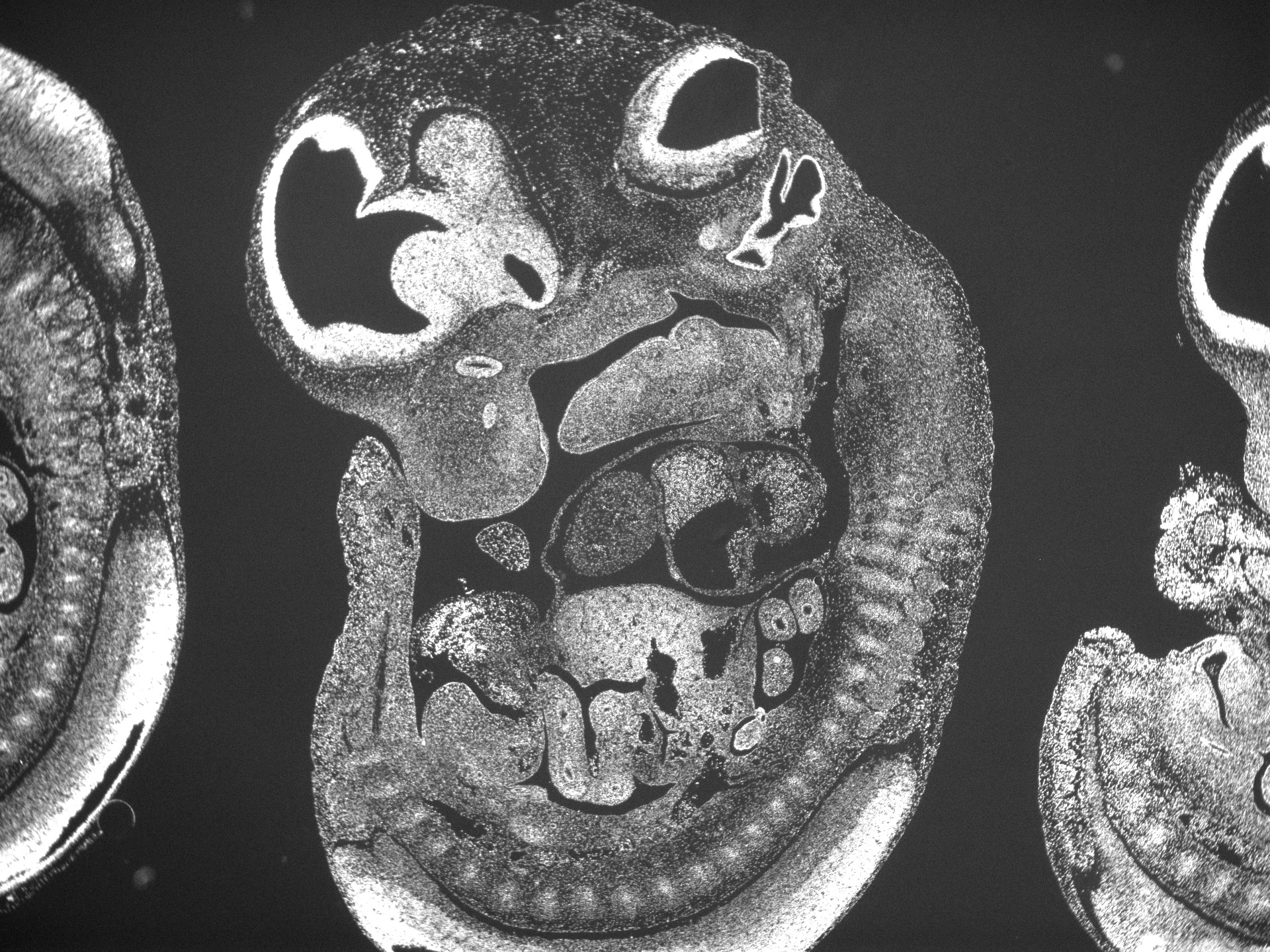

Supplement: Supplementary file 12 — Source data Fig. 1 [file 44321_2025_235_MOESM12_ESM.zip › Figure 1/Figure 1B-1.jpg]

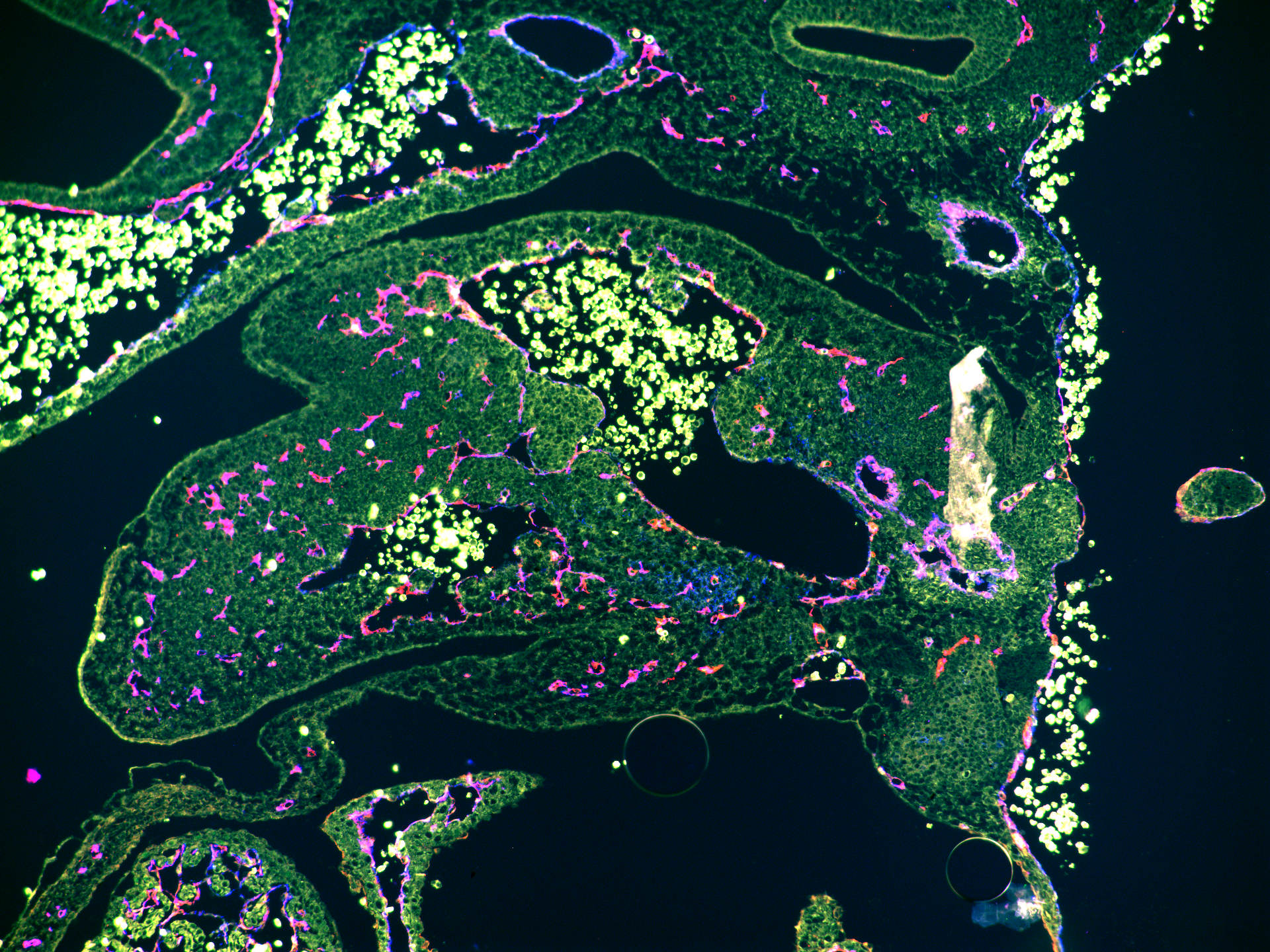

Supplement: Supplementary file 12 — Source data Fig. 1 [file 44321_2025_235_MOESM12_ESM.zip › Figure 1/Figure 1H.tif]

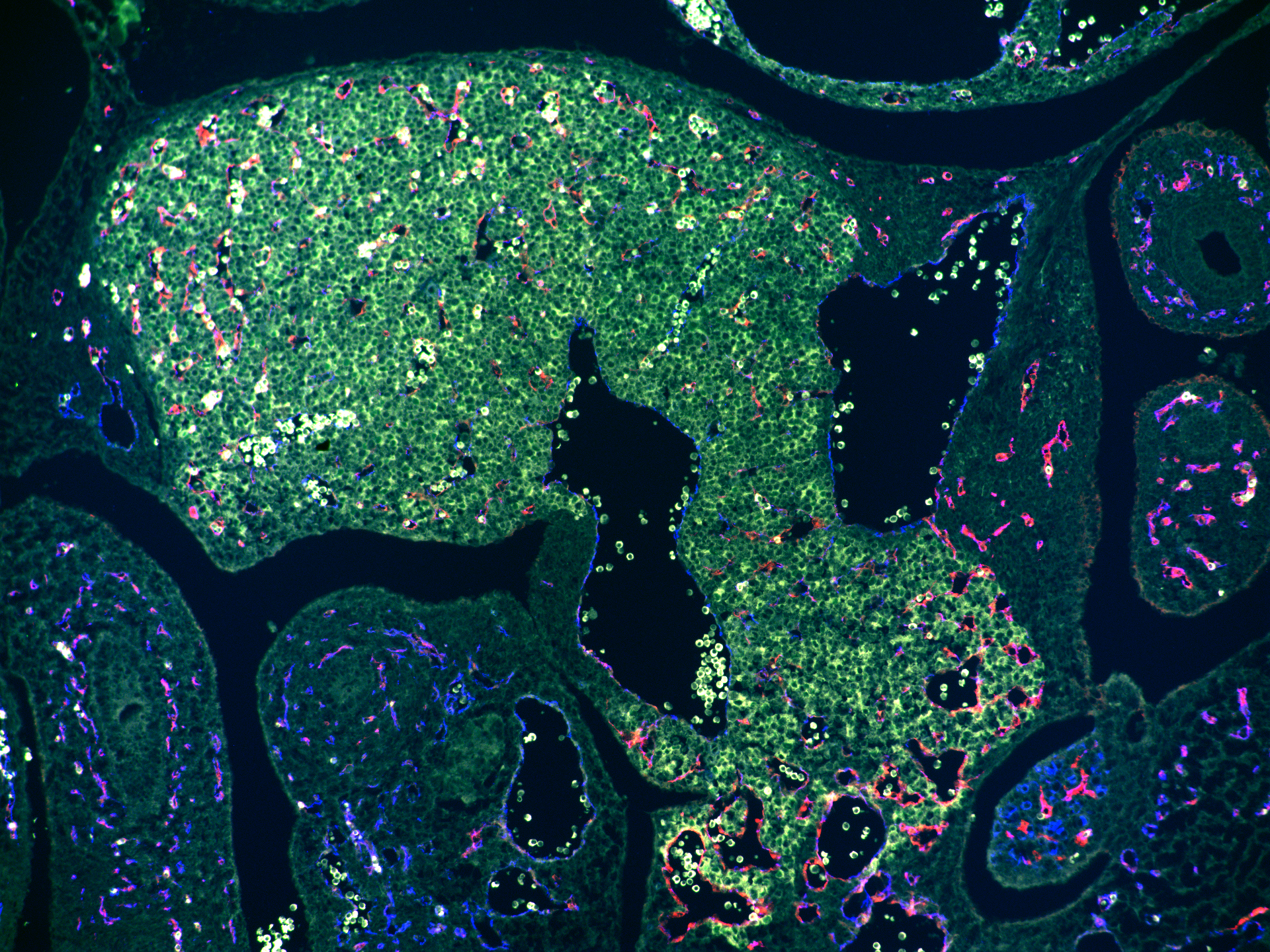

Supplement: Supplementary file 12 — Source data Fig. 1 [file 44321_2025_235_MOESM12_ESM.zip › Figure 1/Figure 1K.tif]

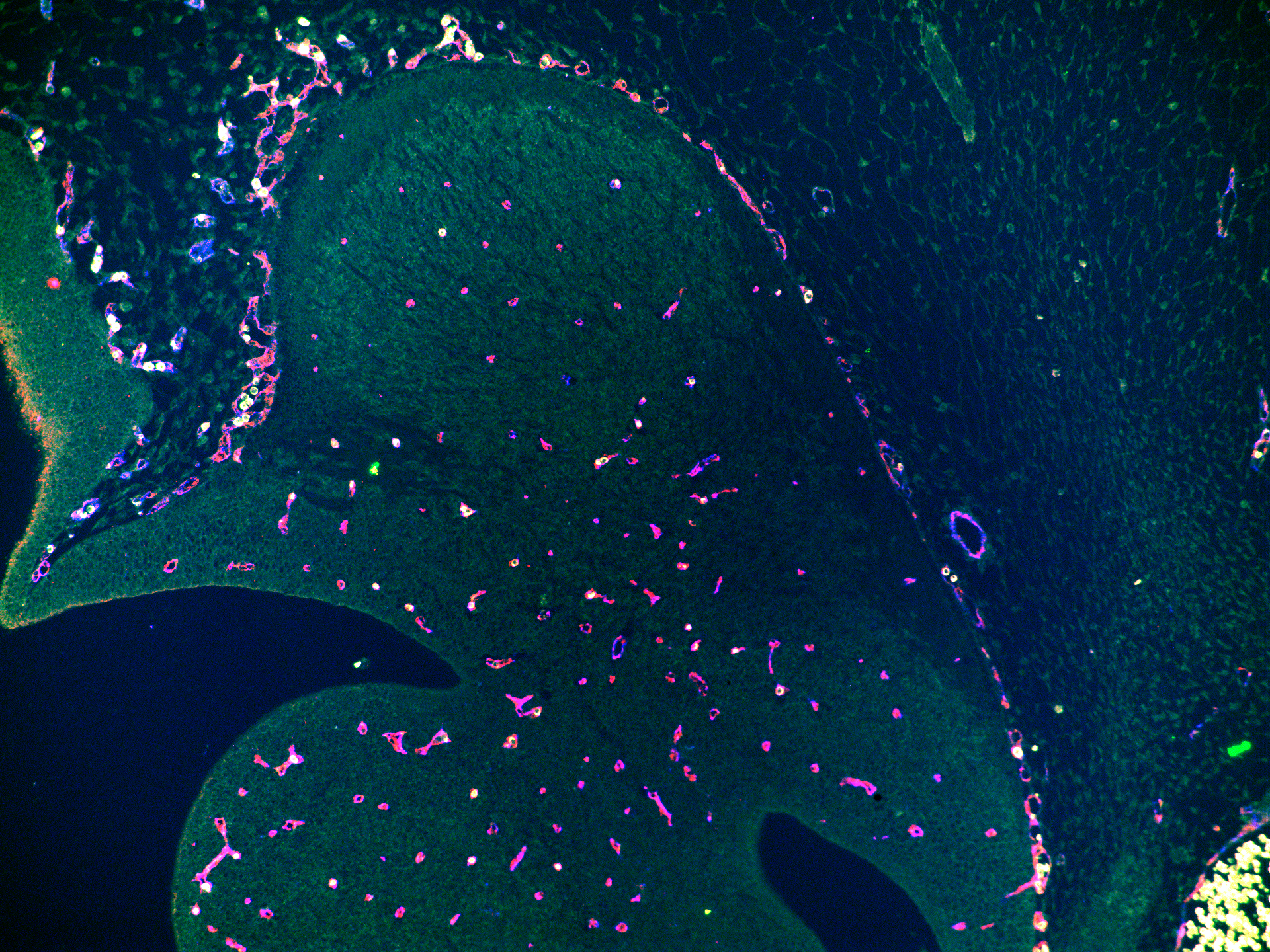

Supplement: Supplementary file 12 — Source data Fig. 1 [file 44321_2025_235_MOESM12_ESM.zip › Figure 1/Figure 1O.tif]

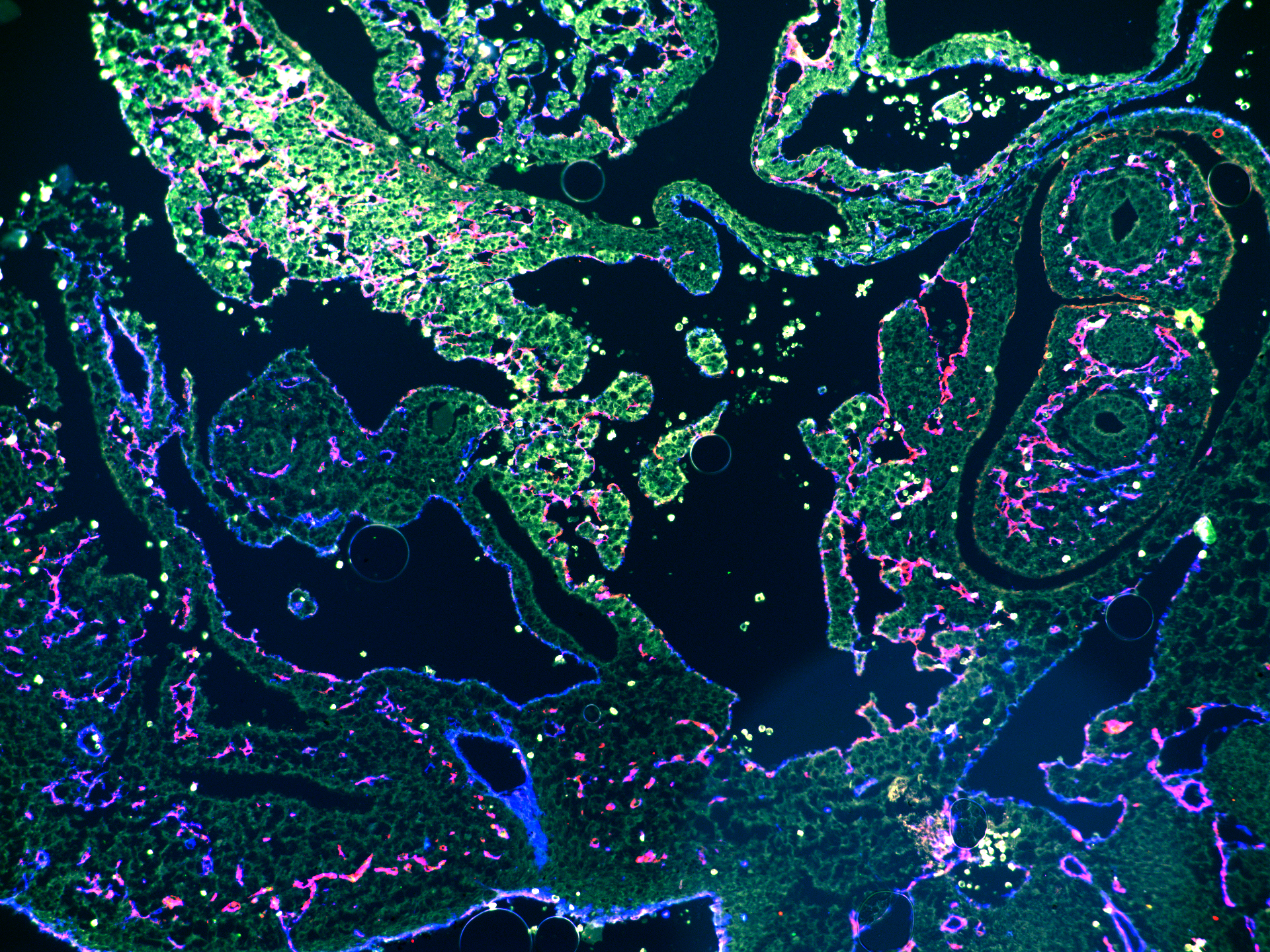

Supplement: Supplementary file 12 — Source data Fig. 1 [file 44321_2025_235_MOESM12_ESM.zip › Figure 1/Figure 1L.tif]

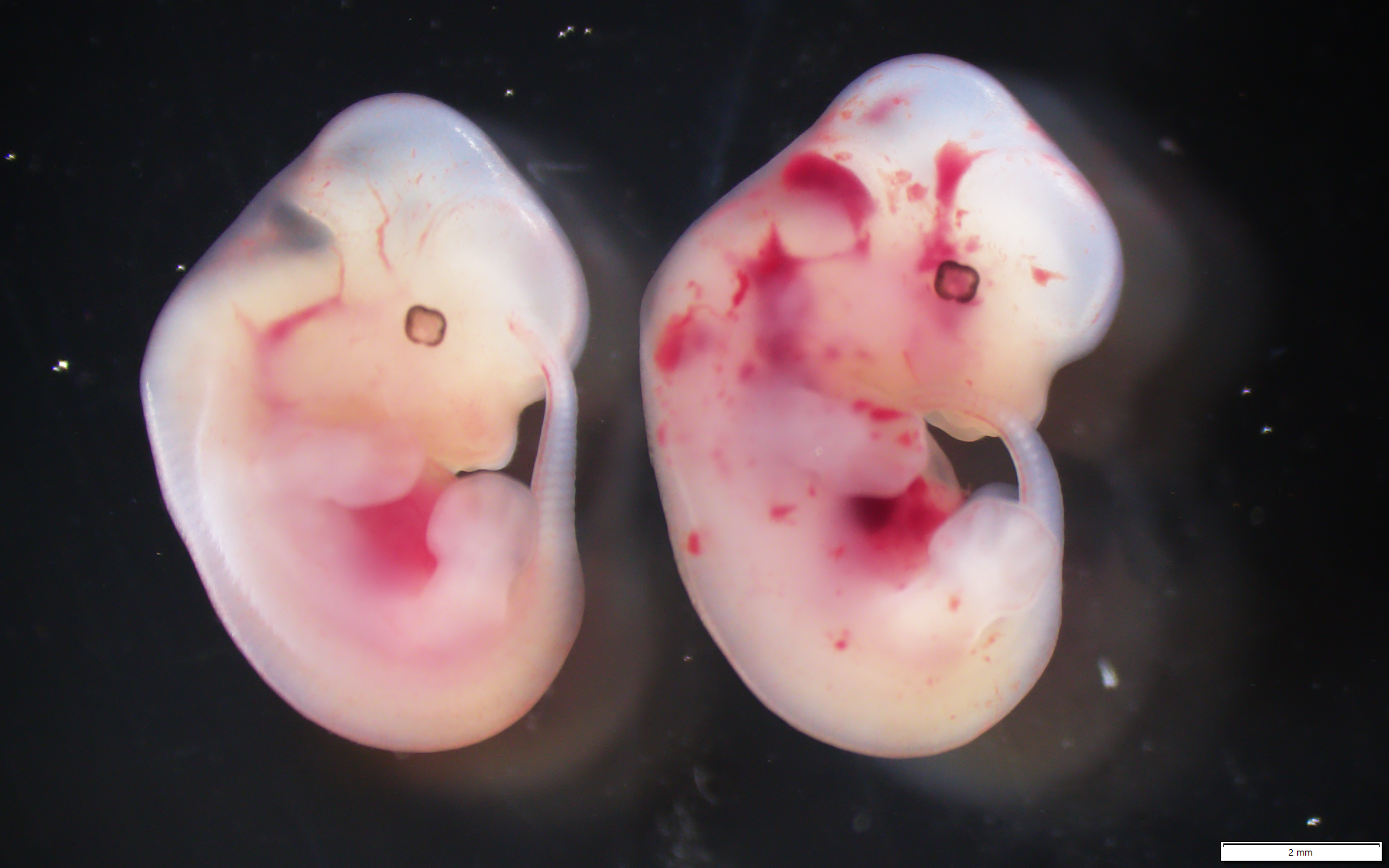

Supplement: Supplementary file 12 — Source data Fig. 1 [file 44321_2025_235_MOESM12_ESM.zip › Figure 1/Figure 1A.tif]

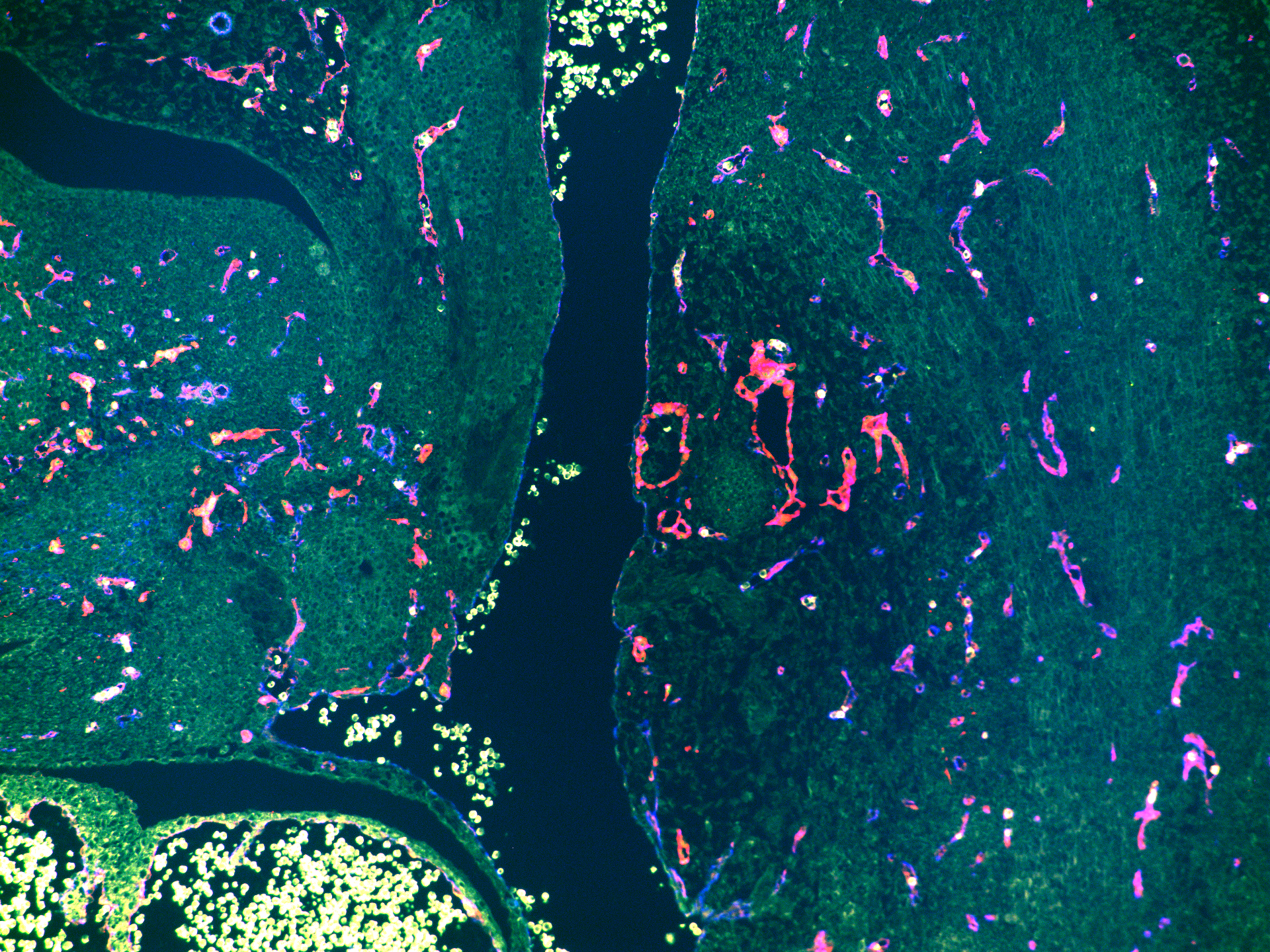

Supplement: Supplementary file 12 — Source data Fig. 1 [file 44321_2025_235_MOESM12_ESM.zip › Figure 1/Figure 1C.tif]

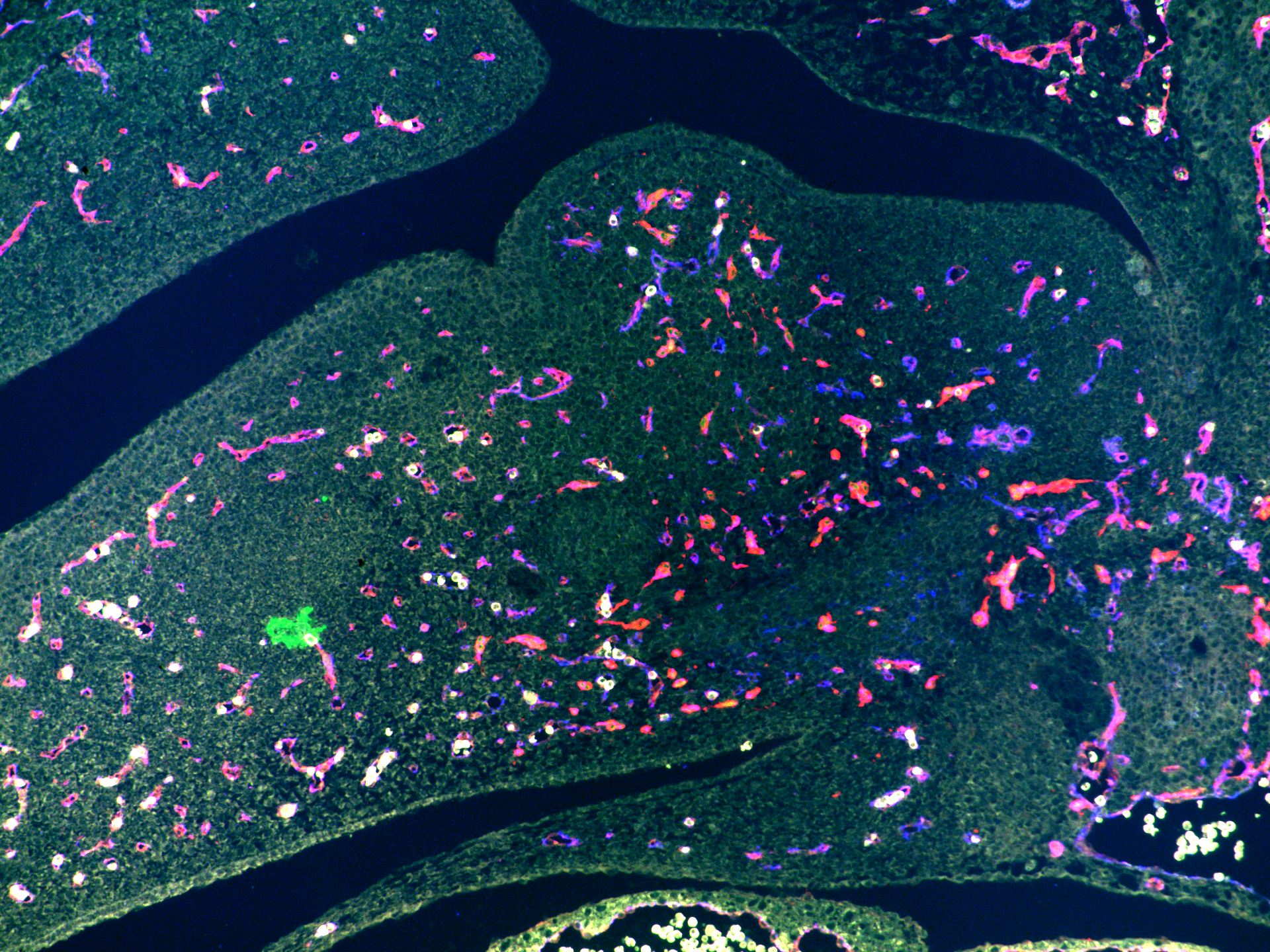

Supplement: Supplementary file 12 — Source data Fig. 1 [file 44321_2025_235_MOESM12_ESM.zip › Figure 1/Figure 1G.tif]

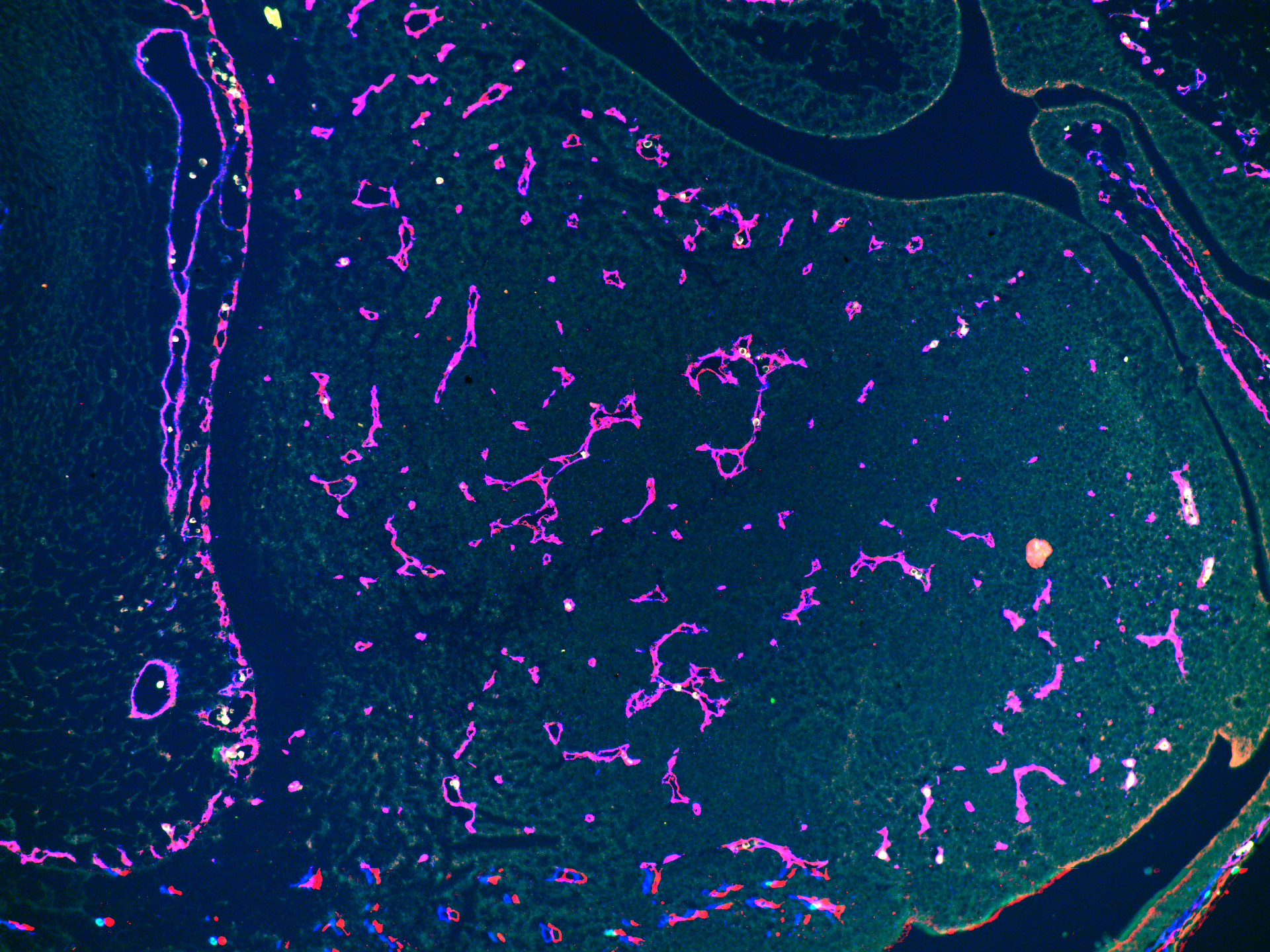

Supplement: Supplementary file 12 — Source data Fig. 1 [file 44321_2025_235_MOESM12_ESM.zip › Figure 1/figure 1P.tif]

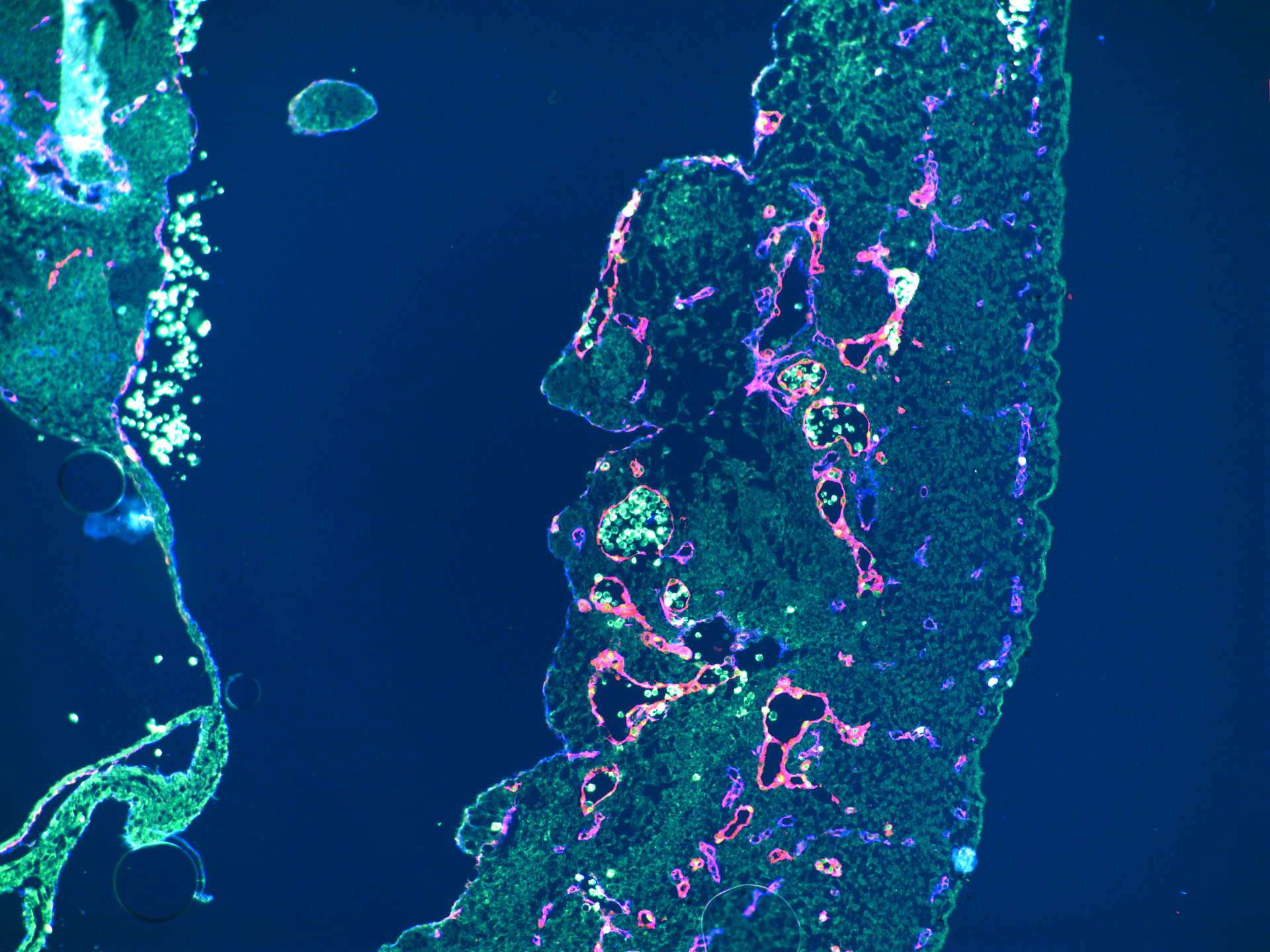

Supplement: Supplementary file 12 — Source data Fig. 1 [file 44321_2025_235_MOESM12_ESM.zip › Figure 1/Figure 1D.tif]
