# Supplementary material for: Embryological cellular origins and hypoxia-mediated mechanisms in PIK3CA-driven refractory vascular malformations
Source: EMBO Mol Med. 2025 Apr 16;17(6):1289–324. doi: 10.1038/s44321-025-00235-1 (PMC12162881; doi:10.1038/s44321-025-00235-1)
Supplement: Supplementary file 13 — Expanded View Figures [file 44321_2025_235_MOESM13_ESM.pdf]

Expanded View Figures

Figure EV1. Pik3ca^{H1047R} expression in *Tie2-Cre* embryos does not significantly affect the endothelium of the aorta, pulmonary artery, or endocardium.

(**A**) Gross morphology of the control and *Tie2-Cre; R26R-Pik3ca^{H1047R}* mutant embryos at E12.5. Comparisons were made between control and mutant embryos ($n = 5$, 5 for each group). In the control, eYFP is not expressed, but in the mutant, eYFP is expressed throughout the body. (**B–K″**) Immunostaining of sagittal sections with the indicated antibodies. An increase in Ki67$^+$ cells in the cardinal vein ECs is observed (**B″″**, **C″″**, red arrows). In mutant mice, eYFP$^+$ cells are observed in both cardinal vein ECs and LECs (**B″″′**, **C″″′**, red arrows). (**D–K″**) Similar analysis in the pulmonary artery, descending aorta, and endocardium. The region outlined by a box in the original image is displayed in an enlarged view in a corresponding panel. (**L–Q**) Statistical analysis of the indicated parameters. CV, cardinal vein; Ao, aorta; PA, pulmonary artery. Each dot represents a value obtained from one sample. Scale bars, 100 μm (**B′–B″″′**, **C′–E″″′**, **D–K″**) and 1 mm (**A**). The nonparametric Mann–Whitney *U* test was used for statistical analysis, with exact p-values indicated. ns ≥0.05. Error bars represent the mean ± standard error of the mean (SEM).

 © The Author(s)

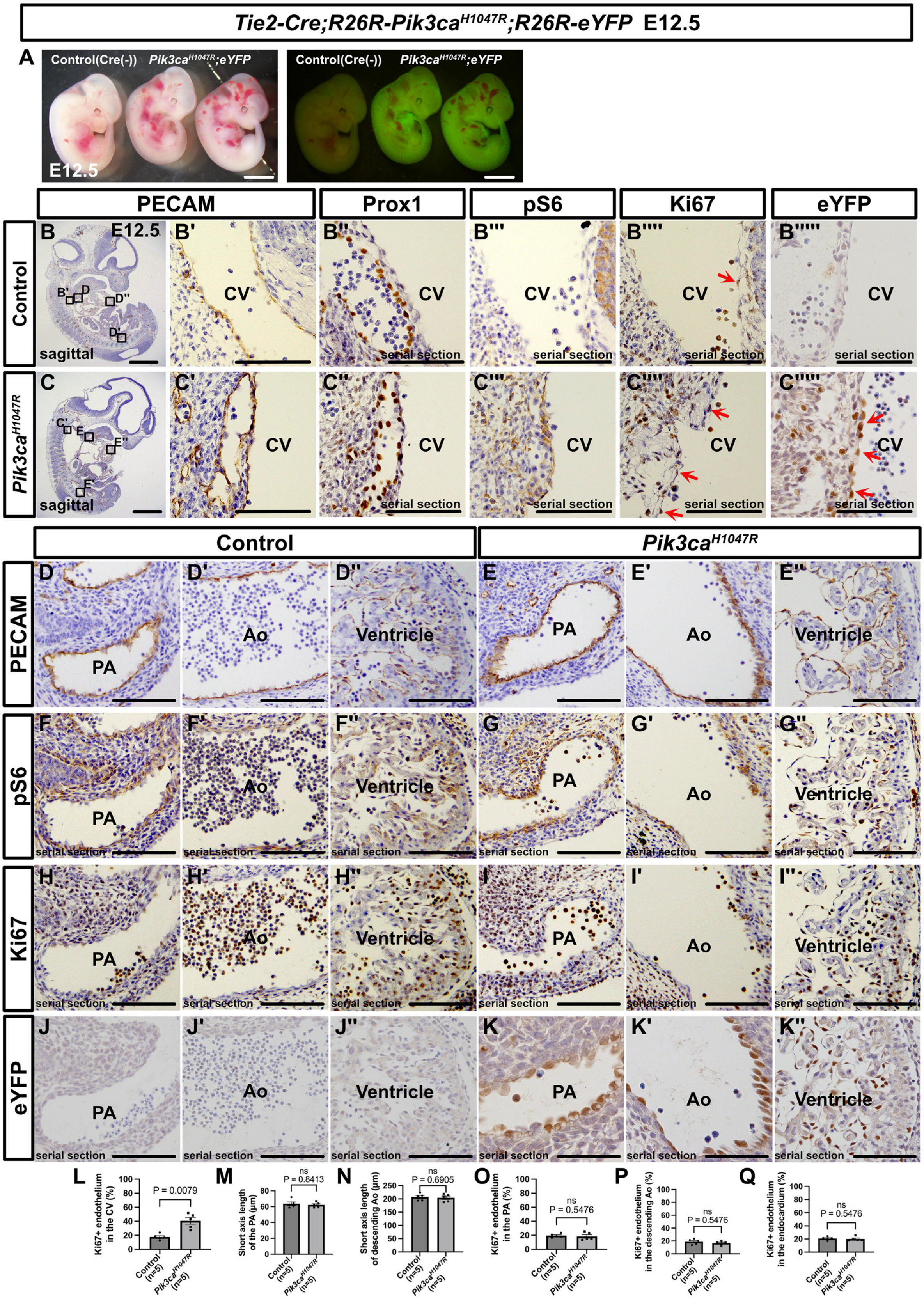

Tie2-Cre;R26R-Pik3caH1047R;R26R-eYFP E12.5
A
Control(Cre(-)) Pik3caH1047R;eYFP
E12.5
PECAM
Prox1
pS6
Ki67
eYFP
Control
Pik3caH1047R
sagittal
serial section
CV
Control
Pik3caH1047R
PA
Ao
Ventricle
L
M
N
O
P
Q
Ki67+ endothelium in the CV (%)
P = 0.0079
Short axis length of the PA (μm)
ns P = 0.8413
Short axis length of descending Ao (μm)
ns P = 0.6905
Ki67+ endothelium in the PA (%)
ns P = 0.5476
Ki67+ endothelium in the descending Ao (%)
ns P = 0.5476
Ki67+ endothelium in the endocardium (%)
ns P = 0.5476
Control (n=5)
Pik3caH1047R (n=5)

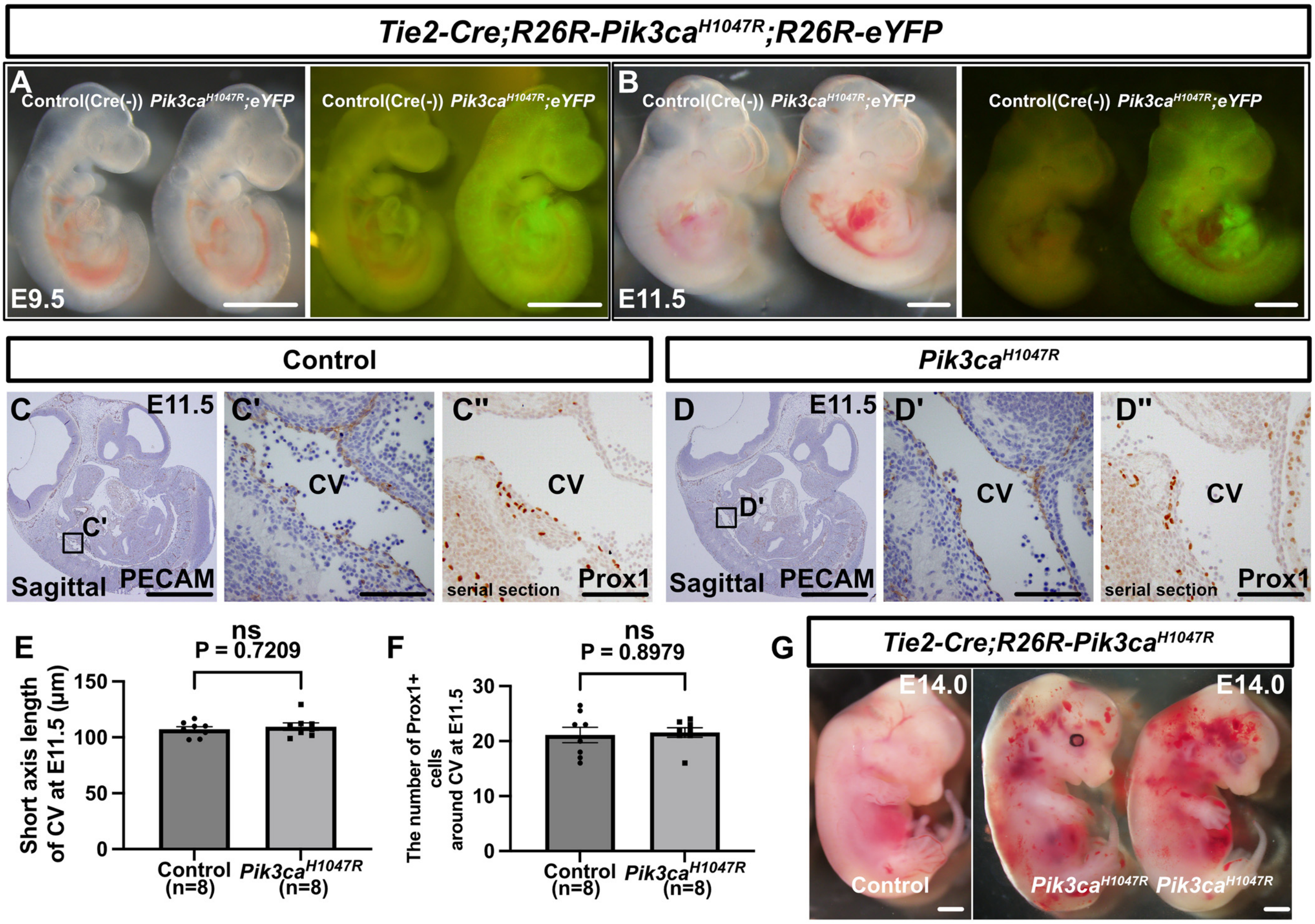

Figure EV2. Pik3ca^{H1047R} expression in *Tie2-Cre* embryos does not produce a discernible phenotype at E11.5.

(**A**, **B**) Gross morphology of control and *Tie2-Cre; R26R-Pik3ca^{H1047R}* mutant embryos at E9.5 ($n = 4$ each for control and mutant) and at E11.5 ($n = 8$ each for control and mutant). (**C–D″**) Immunostaining of sagittal sections with the indicated antibodies at E11.5. (**E**, **F**) Neither overall phenotype, short-axis length of the cardinal vein (CV), nor the number of Prox1^{+} cells around the CV differed between control and mutant embryos. (**G**) Gross morphology of control and *Tie2-Cre; R26R-Pik3ca^{H1047R}* mutant embryos at E14.0. The region outlined by a box in the original image is displayed in an enlarged view in a corresponding panel. Each dot represents a value from a single sample. CV cardinal vein. Scale bars, 100 µm (**C′–C″**, **D′–D″**) and 1 mm (**A**, **B**, **C**, **D**). Statistical analysis was performed using the nonparametric Mann–Whitney *U* test, with exact *P* values indicated. ns ≥ 0.05. Error bars represent the mean ± standard error of the mean (SEM).

 © The Author(s)

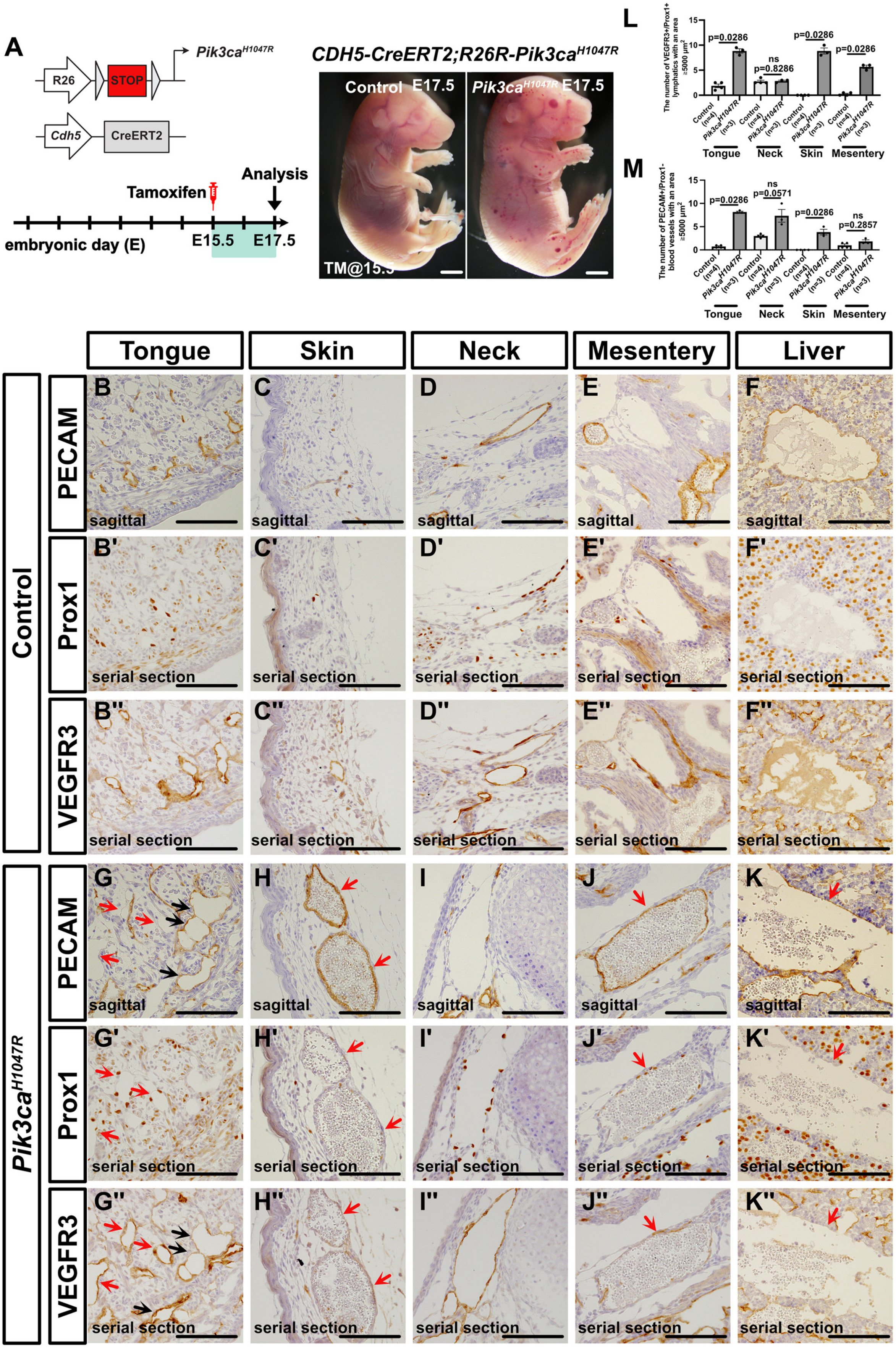
A
Pik3ca H1047R
R26
STOP
Cdh5
CreERT2
Tamoxifen
Analysis
embryonic day (E)
E15.5
E17.5
CDH5-CreERT2;R26R-Pik3ca H1047R
Control E17.5
Pik3ca H1047R E17.5
TM@15.5
L
M
Tongue
Neck
Skin
Mesentery
Tongue
Skin
Neck
Mesentery
Liver
Control
Pik3ca H1047R
PECAM
Prox1
VEGFR3
sagittal
serial section

Figure EV3. Pik3ca^{H1047R} expression in endothelial cells at E15.5 leads to systemic vascular malformations.

(**A**) Schematic of the experimental schedule and gross morphology of control and *CDH5-CreERT2; R26R-Pik3ca^{H1047R}* mutant embryos at E17.5 following tamoxifen administration to pregnant mice at E15.5. Comparisons were made between control and mutant embryos ($n = 4$, 3 for each group). (**B–K″**) Sagittal section immunohistochemistry using the indicated antibodies. (**B–B″**, **G–G″**) In mutant embryos, mildly dilated PECAM$^-$/Prox1$^+$/VEGFR3$^+$ lymphatic vessels (red arrows) and an increased number of PECAM$^+$/Prox1$^-$/VEGFR3$^+$ blood vessels (black arrows) are observed in the tongue. (**C–C″**, **H–H″**) Aberrant, dilated PECAM$^+$/partially Prox1$^+$/ partially VEGFR3$^+$ vasculatures are seen in the skin (red arrows). (**D–D″**, **I–I″**) No significant differences in the neck (larynx) are observed between the control and mutant embryos. (**E–E″**, **J–J″**) In the mesentery, aberrant, dilated PECAM$^+$/partially Prox1$^+$/partially VEGFR3$^+$ vasculatures similar to those in the skin are seen (red arrows). (**F–F″**, **K–K″**) In the liver, dilated PECAM$^+$/Prox1$^-$/partially VEGFR3$^+$ blood vessels are observed. The number of PECAM+ vessels with an area ≥20,000 μm^2 in the liver is 0 ± 0 (median ± SEM) ($n = 4$) in controls and 1.5 ± 0.33 (median ± SEM) ($n = 3$) in mutants ($P = 0.0286$). (**L**, **M**) Statistical analysis in the tongue, neck, skin, and mesentery. Each dot represents a value obtained from one sample. Scale bars: 100 μm (**B–K″**) and 2 mm (**A**). The nonparametric Mann–Whitney *U* test was used for statistical analysis, with exact *P* values indicated. ns ≥ 0.05. Error bars represent the mean ± standard error of the mean (SEM).

 © The Author(s)

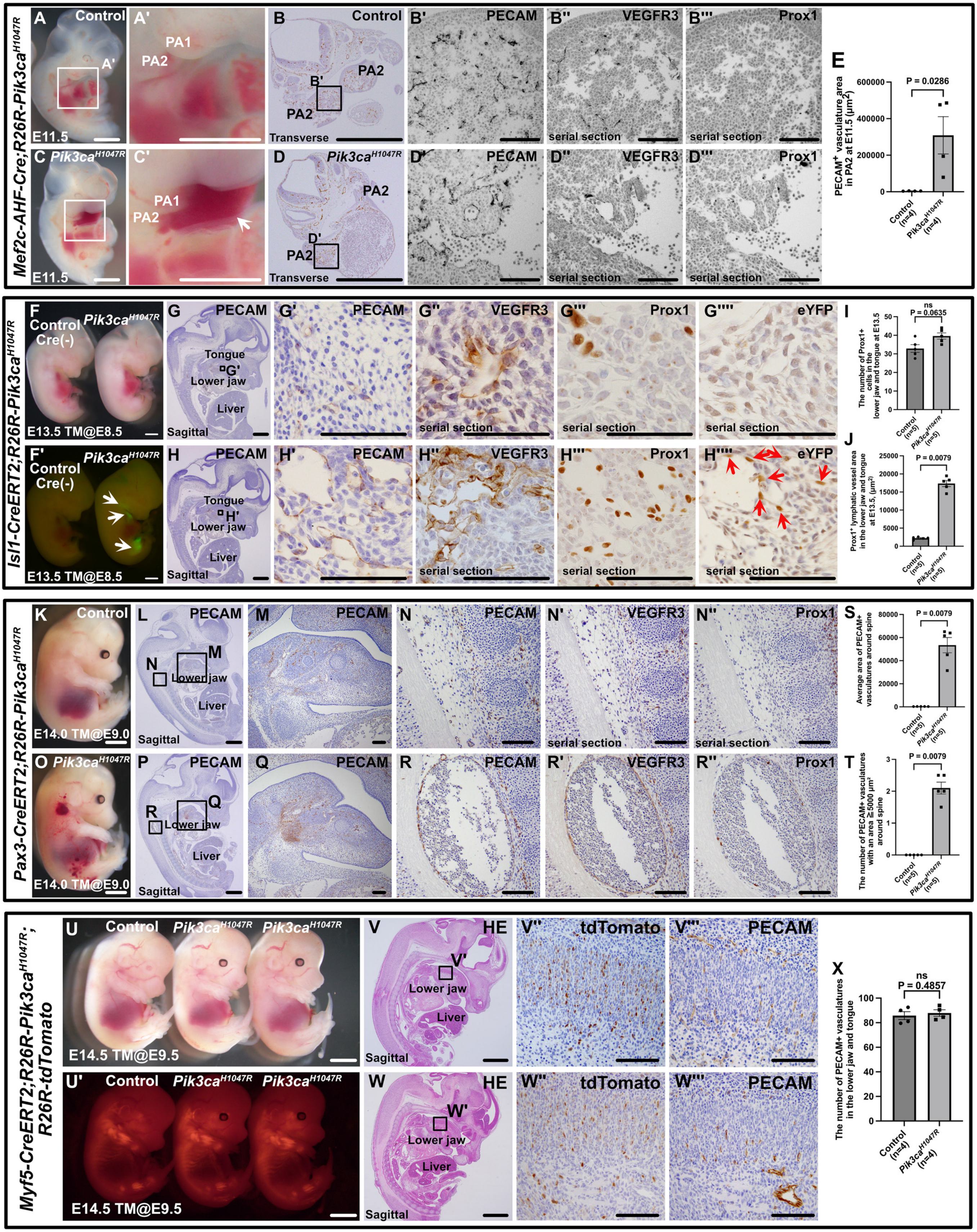

Figure EV4. Pik3ca^{H1047R} expression in CPM induces vascular malformations in the head and neck.

(**A**, **A′**, **C**, **C′**) Gross morphology of the control and *Mef2c-AHF-Cre; R26R-Pik3ca^{H1047R}* mutant embryos at E11.5. In mutant embryos, enlarged blood-filled vasculatures were evident in PA1 and PA2 (white arrow). Comparisons were made between control and mutant embryos ($n = 4$, 4 for each group). (**B–B‴**, **D–D‴**) Transverse section immunostaining with the indicated antibodies. (**E**) Mutant embryos show dilated PECAM^{+}/Prox1^{-}/partially VEGFR3^{+} blood vessels extending from the first to the second pharyngeal arches. (**F**, **F′**) Gross morphology of the control and *Isl1-CreERT2; R26R-Pik3ca^{H1047R}; R26R-eYFP* mutant embryos at E13.5, after tamoxifen administration at E8.5. Comparisons were made between control and mutant embryos ($n = 5$, 5 for each group). eYFP expression is observed from the lower jaw to the neck and outflow tracts, and in the genital region (white arrows). (**G–G⁗**, **H–H⁗**, **I**, **J**) Mutant embryos show dilated PECAM^{+}/Prox1^{+}/VEGFR3^{+} lymphatic vessels between the lower jaw and tongue. The number of Prox1^{+} cells in these lymphatic vessels did not differ between the control and mutant groups. These vessels are also eYFP^{+} (red arrows). (**K**, **O**) Gross morphology of the control and *Pax3-CreERT2; R26R-Pik3ca^{H1047R}* mutant embryos at E14.0, after tamoxifen administration at E9.0. Comparisons were made between control and mutant embryos ($n = 5$, 5 for each group). (**L–N″**, **P–R″**, **S**, **T**) No vascular malformations are seen in the head and neck of mutant embryos, but dilated PECAM^{+}/partially Prox1^{+}/VEGFR3^{+} blood-filled vessels are observed around the spine. (**U**, **U′**) Gross morphology of the control and *Myf5-CreERT2; R26R-Pik3ca^{H1047R}; R26R-tdTomato* mutant embryos at E14.5, after tamoxifen administration at E9.5. Comparisons were made between control and mutant embryos ($n = 4$, 4 for each group). (**V**, **W**) Sagittal section H&E staining. (**V″**, **V‴**, **W″**, **W‴**) Sagittal section immunostaining with the indicated antibodies. (**X**) No differences were observed in the number of PECAM^{+} vasculatures in the lower jaw and tongue. PA pharyngeal arch. The region outlined by a box in the original image is displayed in an enlarged view in a corresponding panel. Scale bars: 100 μm (**B′–B‴**, **D′–D‴**, **G′–G⁗**, **H′–H⁗**, **M–N″**, **Q–R″**, **V″**, **V‴**, **W″**, **W‴**), 1 mm (**A**, **B**, **C**, **D**, **G**, **H**, **K**, **L**, **O**, **P**, **V**, **W**), and 2 mm (**F**, **F′**, **U**, **U′**). The nonparametric Mann–Whitney *U* test was used for statistical analysis, with exact *P* values indicated. ns ≥ 0.05. Error bars represent the mean ± standard error of the mean (SEM).

 © The Author(s)

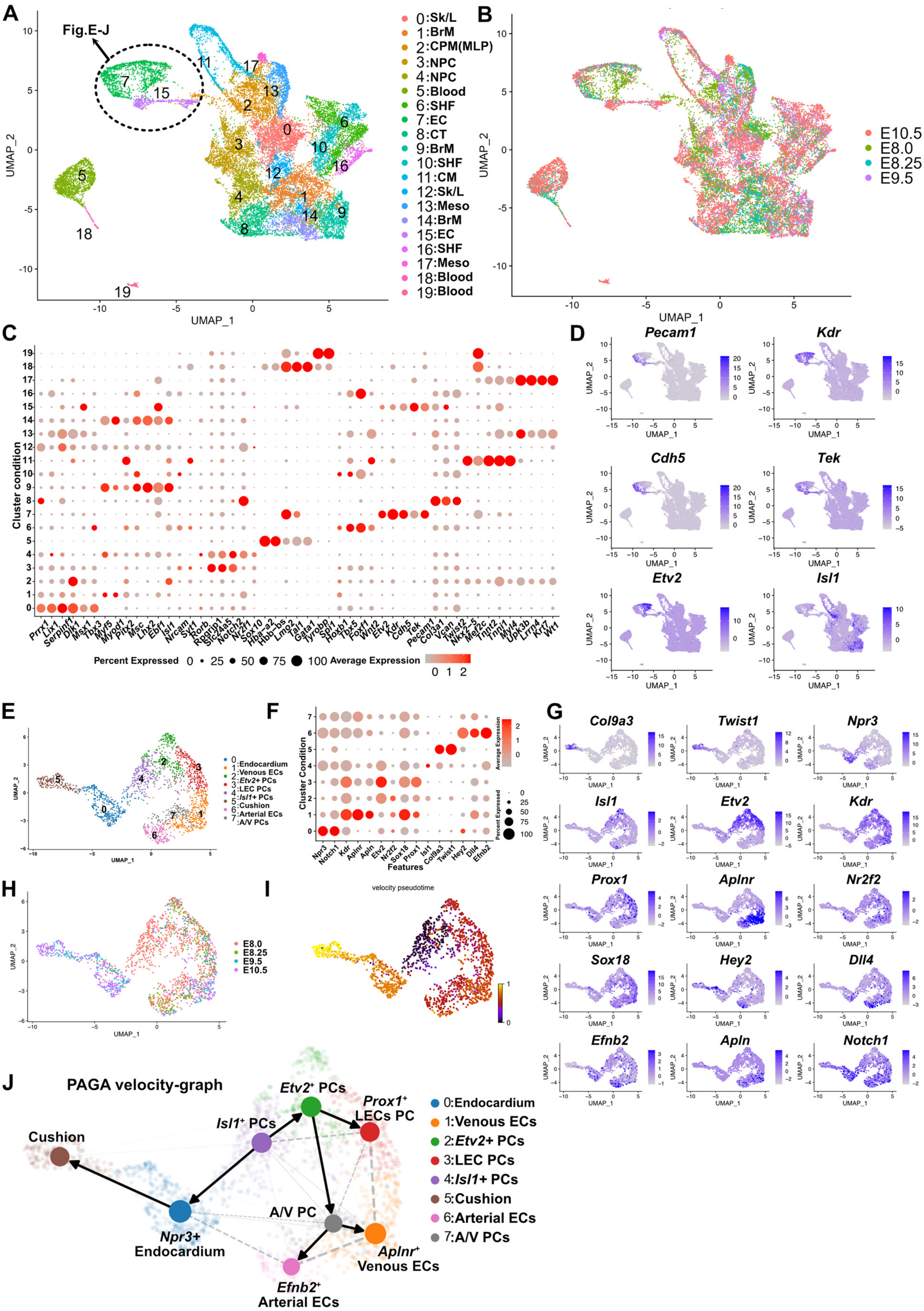

A
Fig.E-J
0:Sk/L
1:BrM
2:CPM(MLP)
3:NPC
4:NPC
5:Blood
6:SHF
7:EC
8:CT
9:BrM
10:SHF
11:CM
12:Sk/L
13:Meso
14:BrM
15:EC
16:SHF
17:Meso
18:Blood
19:Blood
UMAP_1
UMAP_2
B
E10.5
E8.0
E8.25
E9.5
C
Cluster condition
Prrx1 Lix1 Serpinf1 Dlk1 Msx1 Tbx3 Myf5 Myod1 Pitx2 Msc Lhx2 Ebf1 Isl1 Nrcam Syt1 Rorb Rgs5 Rspo1 Slc4a5 Notch2 Nr2f1 Sox10 Hba-a2 Hbb-bs Lmo2 Tal1 Gata1 Tyrobp Spi1 Hoxb1 Tbx5 Foxf1 Wnt2 Etv2 Kdr Cdh5 Tek Pecam1 Col3a1 Vcan Twist2 Nkx2-5 Mef2c Tnnt2 Tnni1 Myl4 Upk3b Lrrn4 Krt7 Wt1
Percent Expressed 0 25 50 75 100
Average Expression 0 1 2
D
Pecam1
Kdr
Cdh5
Tek
Etv2
Isl1
E
0:Endocardium
1:Venous ECs
2:Etv2+ PCs
3:LEC PCs
4:Isl1+ PCs
5:Cushion
6:Arterial ECs
7:A/V PCs
F
Cluster Condition
Npr3 Notch1 Kdr Aplnr Apln Etv2 Nr2f2 Sox18 Prox1 Isl1 Col9a3 Twist1 Hey2 Dll4 Efnb2
Features
Average Expression
Percent Expressed
G
Col9a3
Twist1
Npr3
Isl1
Etv2
Kdr
Prox1
Aplnr
Nr2f2
Sox18
Hey2
Dll4
Efnb2
Apln
Notch1
H
E8.0
E8.25
E9.5
E10.5
I
velocity pseudotime
J
PAGA velocity-graph
Etv2+ PCs
Prox1+ LECs PC
Isl1+ PCs
Cushion
A/V PC
Npr3+ Endocardium
Aplnr+ Venous ECs
Efnb2+ Arterial ECs
0:Endocardium
1:Venous ECs
2:Etv2+ PCs
3:LEC PCs
4:Isl1+ PCs
5:Cushion
6:Arterial ECs
7:A/V PCs

Figure EV5. Predicted differentiation lineage of endothelial cells from the CPM.

(**A–G**) Re-analysis of scRNA-seq data (Nomaru et al, 2021) of *Mesp1*$^{+}$ CPM at E8.0-E10.5. (**A**) UMAP plot with clusters color-coded (0–19): 0: Sk/L, skeleton/limb progenitor cells; 1: BrM, branchial muscle; 2: CPM (MLP), cardiopharyngeal mesoderm (multilineage progenitor cells); 3, 4: NPC, neural progenitor cells; 5: blood, blood cells; 6: SHF, second heart field; 7: EC, endothelial cells; 8: CT, Connective tissue; 9: BrM, branchial muscle; 10: SHF, second heart field; 11: CM, cardiomyocyte; 12: Sk/L, skeleton/limb progenitor cells; 13: Meso, mesothelium; 14: BrM, branchial muscle; 15: EC, endothelial cell; 16: SHF, second heart field; 17: Meso, mesothelium; 18,19: blood, blood cells. Black dotted circle indicates endothelial clusters, which underwent sub-clustering analysis. (**B**) UMAP plot color-coded by embryonic day: green (E8.0), light blue (E8.25), purple (E9.5), and red (E10.5). (**C**) Heatmap displaying the average expression levels of marker genes per cluster. (**D**) UMAP plot showing the expression levels of endothelial markers. (**E**) UMAP plot with clusters color-coded (0-7): 0: Endocardium; 1: Venous EC, venous endothelial cells; 2: *Etv2*$^{+}$ PCs, *Etv2*$^{+}$ endothelial progenitor cells; 3: LEC PCs, lymphatic endothelial progenitor cells; 4: *Isl1*$^{+}$ PCs, *Isl1*$^{+}$ endothelial progenitor cells; 5: Cushion, cushion tissue; 6: Arterial ECs, arterial endothelial cells; 7: A/V PCs, arterial and venous endothelial progenitor cells. (**F**) Heatmap displaying the average expression levels of marker genes per cluster. (**G**) Heatmap displaying the average expression levels of marker genes per cluster. (**H**) UMAP plot, color-coded by embryonic day, with red (E8.0), green (E8.25), light blue (E9.5), and purple (E10.5). (**I**) UMAP plot representing pseudotime calculated from RNA velocity analysis. (**J**) PAGA graph illustrating the predicted differentiation lineage of CPM-derived endothelial cells based on RNA velocity.

 © The Author(s)

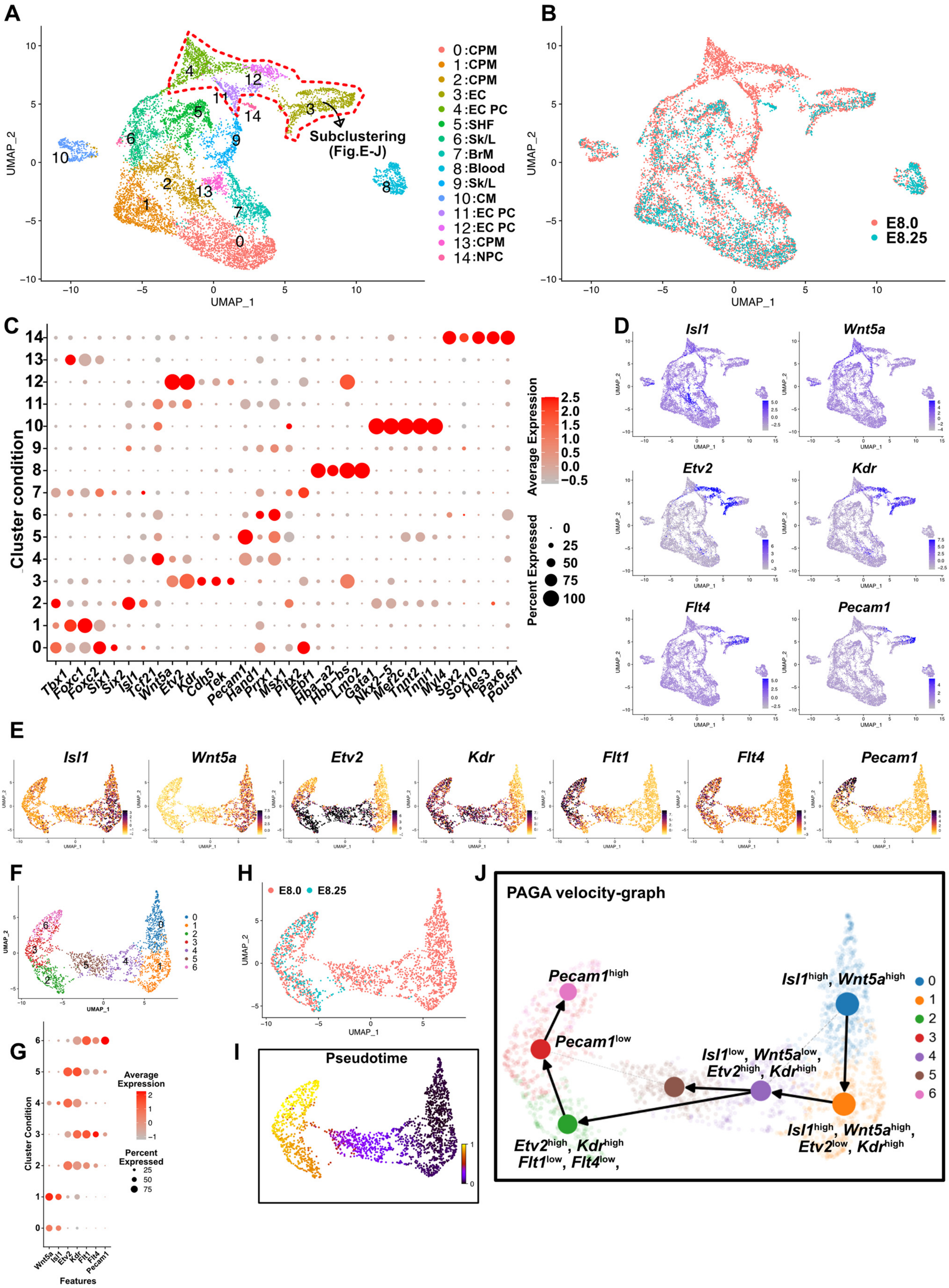
A
0:CPM
1:CPM
2:CPM
3:EC
4:EC PC
5:SHF
6:Sk/L
7:BrM
8:Blood
9:Sk/L
10:CM
11:EC PC
12:EC PC
13:CPM
14:NPC
Subclustering (Fig.E-J)
UMAP_1
UMAP_2
B
E8.0
E8.25
C
Cluster condition
Average Expression
Percent Expressed
Tbx1 Foxc1 Foxc2 Six1 Six2 Isl1 Tcf21 Wnt5a Etv2 Kdr Cdh5 Tek Pecam1 Hand1 Prrx1 Msx1 Pitx2 Ebf1 Hba-a2 Hbb-bs Lmo2 Gata1 Nkx2-5 Mef2c Tnnt2 Tnni1 Myl4 Sox2 Sox10 Hes3 Pax6 Pou5f1
D
Isl1
Wnt5a
Etv2
Kdr
Flt4
Pecam1
E
Isl1
Wnt5a
Etv2
Kdr
Flt1
Flt4
Pecam1
F
G
Average Expression
Percent Expressed
Cluster Condition
Features
Wnt5a Isl1 Etv2 Kdr Flt1 Flt4 Pecam1
H
E8.0
E8.25
I
Pseudotime
J
PAGA velocity-graph
Pecam1high
Pecam1low
Isl1high, Wnt5ahigh
Isl1low, Wnt5alow, Etv2high, Kdrhigh
Isl1high, Wnt5ahigh, Etv2low, Kdrhigh
Etv2high, Kdrhigh Flt1low, Flt4low,

◀ **Figure EV6. The downregulation of *Isl1* and the expression of *Etv2* drive the differentiation of endothelial cells from the CPM.**

(**A**) UMAP plot color-coded by cluster (0-14): 0, 1, 2: CPM, cardiopharyngeal mesoderm; 3: EC, endothelial cells; 4: EC PC, endothelial cell progenitors; 5: SHF, second heart field; 6, 9: Sk/L, skeleton/limb progenitor cells; 7: BrM, branchial muscle; 8: blood, blood cell; 9: BrM, branchial muscle; 10: CM, cardiomyocyte; 11, 12: EC PC, endothelial cell progenitors; 13: CPM, cardiopharyngeal mesoderm; 14: NPC, neural progenitor cells. (**B**) UMAP plot color-coded by embryonic day, with red representing E8.0 and light blue representing E8.25. (**C**) Heatmap showing the average expression levels of marker genes for each cluster. (**D**) UMAP plot displaying the expression levels of CPM or endothelial cell marker genes. (**E**) Sub-clustering analysis of clusters 3, 4, 11, and 12, showing the endothelial cell marker genes. (**F**) UMAP plot with clusters color-coded (0-6). (**G**) Heatmap showing the average expression levels of marker genes for each cluster. (**H**) UMAP plot, color-coded by embryonic day (red: E8.0, light blue: E8.25). (**I**) UMAP plot showing pseudotime calculated from RNA velocity analysis. (**J**) PAGA graph illustrating the predicted differentiation trajectory from CPM to endothelial cells based on RNA velocity.

 © The Author(s)

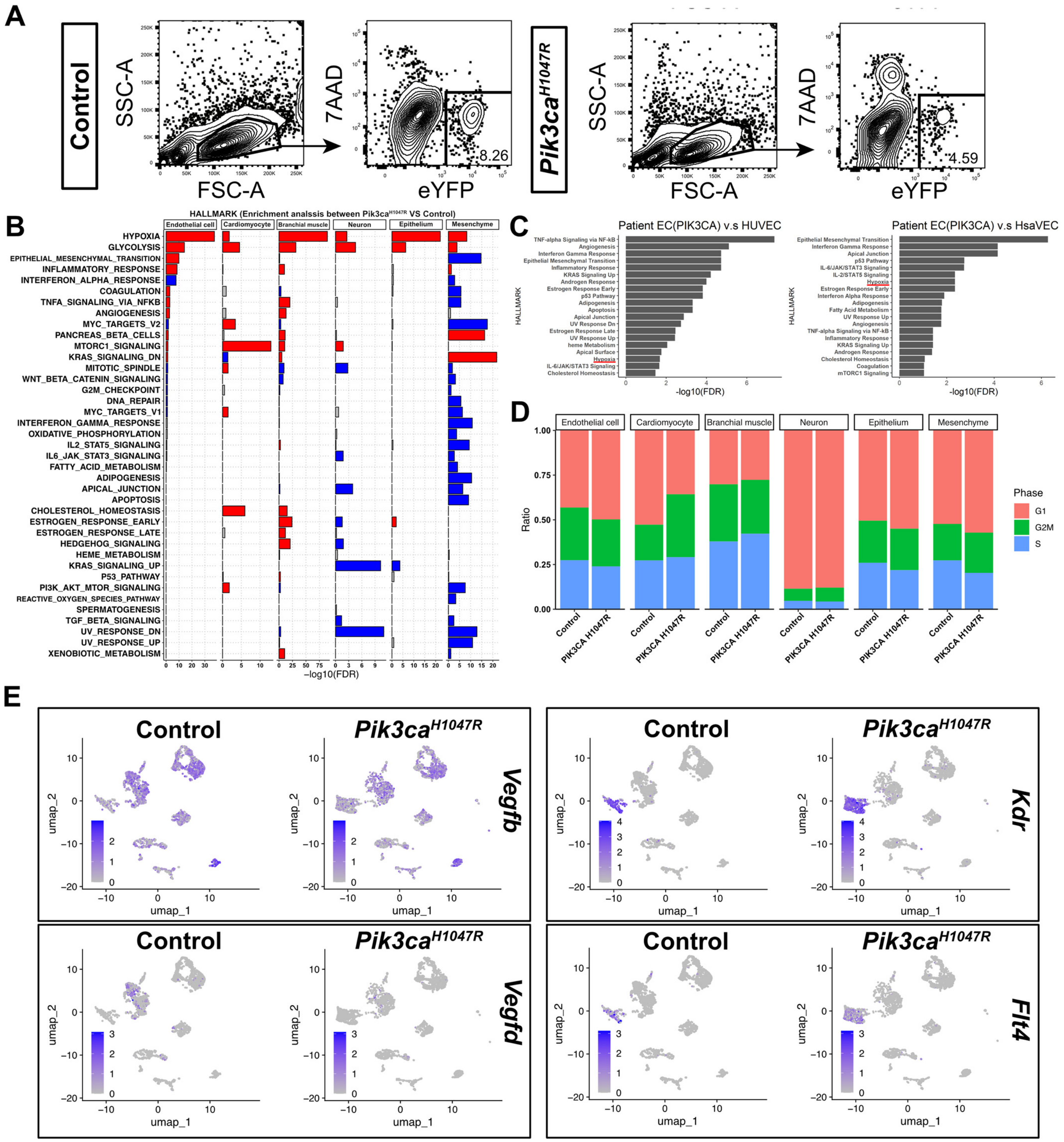

Figure EV7. Pik3ca^{H1047R} expression enhances hypoxia signaling across multiple cell types.

(**A**, **B**, **D**, **E**) scRNA-seq analysis of eYFP^{+} cells sorted by FACS from *Isl1-cre;R26R-eYFP* and *Isl1-cre;R26R-Pik3ca^{H1047R};R26R-eYFP* embryos. (**A**) FACS sorting of eYFP+ cells. (**B**) Enrichment analysis from scRNA-seq comparing different cell types. Each bar chart shows Hallmark gene sets that exhibited significant changes in different cell types when comparing control and mutant groups. Red bars indicate Hallmark gene sets with higher enrichment scores in the mutant group, blue bars indicate higher scores in the control group, and gray bars indicate non-significant gene sets. The *x* axis represents -log10(FDR), and significant Hallmark gene sets were defined as FDR < 0.1. (**C**) Re-analysis of bulk-RNA-seq data (Jauhiainen et al, 2023) from endothelial cells derived from PIK3CA mutated venous malformations (VM) (Patient EC (PIK3CA)) compared to control endothelial cells (Human umbilical venous endothelial cells: HUVEC: or Human saphenous vein endothelial cells: HsaVEC). The left bar chart shows the top 20 significantly enriched Hallmark gene sets in *PIK3CA* mutated Patient ECs compared to HUVEC, while the right chart shows enrichment in *PIK3CA* mutated Patient ECs compared to HsaVEC. Differentially expressed genes were defined as having a fold change >1.5 and FDR < 0.05. The *x* axis represents -log10(FDR), and significant Hallmark gene sets were defined as FDR < 0.1. (**D**) Proportions of cells in each phase of the cell cycle (G1/S/G2-M) across cell types. (**E**) UMAP plots showing expression levels of *Vegfb, Vegfd, Kdr,* and *Flt4* by condition.

© The Author(s)

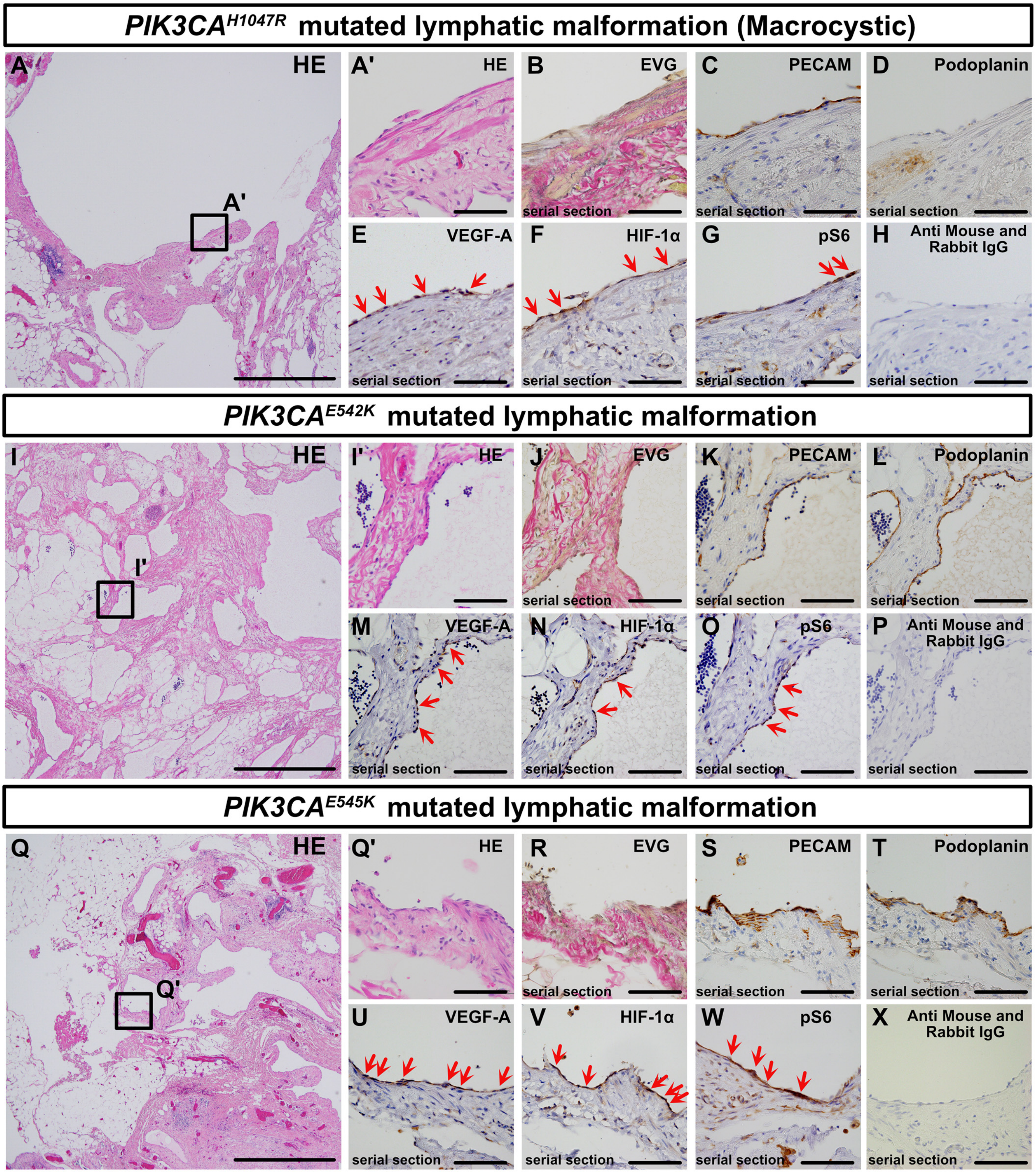

Figure EV8. HIF-1α and VEGF-A expression in malformed lymphatic endothelial cells with *PIK3CA*^{E542K} and *PIK3CA*^{E545K} mutations.

(**A–X**) Hematoxylin and eosin (HE) staining, special stains [elastica van Gieson (EVG), elastica Sirius red (ESR)—collagen fibers appear red, elastic fibers black, muscle tissue yellow], and immunohistochemistry using the indicated antibodies. No signal was detected in the negative controls using only secondary antibodies (**H**, **P**, **X**). (**A–H**) Macrocystic type lymphatic malformations. VEGF-A, HIF-1α, and pS6 are expressed in the ECs of malformed lymphatic vessels (**E–G**, red arrows). (**I–P′′′′′**) Similar expression patterns are observed in lymphatic malformations with the *PIK3CA*^{E542K} mutation, where VEGF-A, HIF-1α, and pS6 are detected in malformed LECs (**M–O**, red arrows). (**Q–X**) The same findings are present in lymphatic malformations with the *PIK3CA*^{E545K} mutation, showing VEGF-A, HIF-1α, and pS6 expression in the malformed endothelial cells (red arrows, **U–X**). The region outlined by a box in the original image is displayed in an enlarged view in a corresponding panel. Scale bars: 100 μm (**A′–H**, **I′–P**, **Q′–X**), 1 mm (**A**, **I**, **Q**).

 © The Author(s)

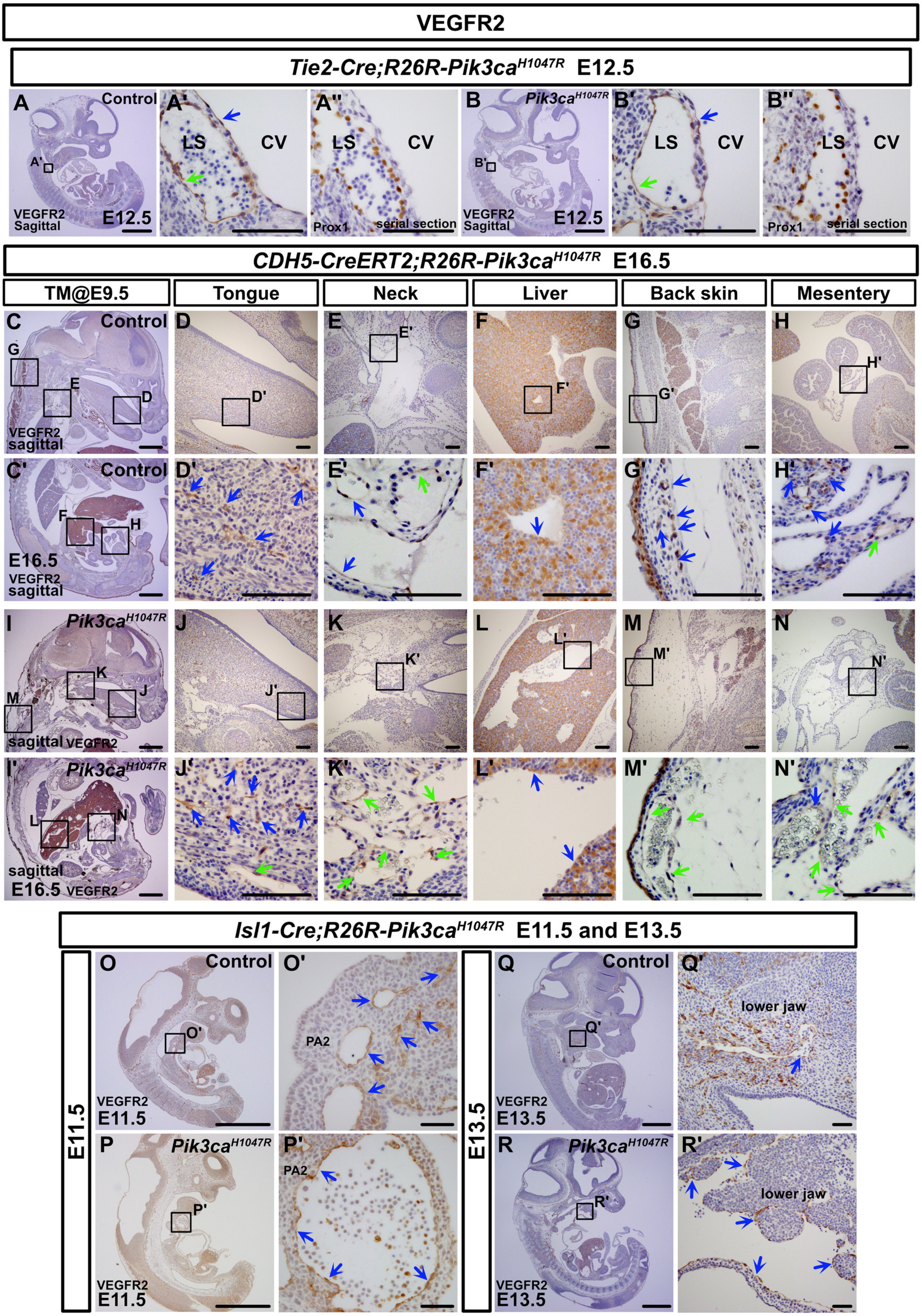
VEGFR2
Tie2-Cre;R26R-Pik3caH1047R E12.5
A Control A' LS CV A'' LS CV Prox1 serial section
VEGFR2 Sagittal E12.5
B Pik3caH1047R B' LS CV B'' LS CV Prox1 serial section
VEGFR2 Sagittal E12.5
CDH5-CreERT2;R26R-Pik3caH1047R E16.5
TM@E9.5
Tongue
Neck
Liver
Back skin
Mesentery
C Control VEGFR2 sagittal
C' Control E16.5 VEGFR2 sagittal
I Pik3caH1047R sagittal VEGFR2
I' Pik3caH1047R sagittal E16.5 VEGFR2
Isl1-Cre;R26R-Pik3caH1047R E11.5 and E13.5
E11.5
O Control VEGFR2 E11.5
O' PA2
P Pik3caH1047R VEGFR2 E11.5
P' PA2
E13.5
Q Control VEGFR2 E13.5
Q' lower jaw
R Pik3caH1047R VEGFR2 E13.5
R' lower jaw

Figure EV9. VEGFR2 is broadly expressed in blood and lymphatic vessels during embryogenesis.

(**A–R′**) Sagittal sections were immunostained with the indicated antibodies. In the serial sections, lymphatic vessels can be distinguished from blood vessels based on their anatomical characteristics (in panels **A–B**″, Prox1 staining delineates the position of lymphatic vessels; in (**C–R**′), lymphatic vessels can be identified by their location in the serial sections in Figs. 2 and 3). Lymphatic vessels are marked with green arrows, and blood vessels with blue arrows. (**A–B**″) *Tie2-Cre; R26R-Pik3ca^{H1047R}* control and mutant embryos at E12.5, serial sections from Fig. 1B. (**C–N**′) *CDH5-CreERT2; R26R-Pik3ca^{H1047R}* control and mutant embryos treated with tamoxifen at E9.5 and analyzed at E16.5, serial sections from Fig. 2C, C′, J, J′. (**O–R**′) *Isl1-Cre; R26R-Pik3ca^{H1047R}* control and mutant embryos at E11.5 and E13.5, serial sections from Fig. 3B, C, E, H. CV cardinal vein, LS lymph sac, PA2 second pharyngeal arch. The region outlined by a box in the original image is displayed in an enlarged view in a corresponding panel. Figure EV9A″ is the same sample image as Fig. EV1B″, but it has been reused to demonstrate the Prox1^{+} lymphatic vessels. Scale bars: 100 μm (**A**′, **A**″, **B**′, **B**″, **D–H**, **D**′–**H**′, **J–N**, **J**′–**N**′, **O**′, **P**′, **Q**′, **R**′), and 1 mm (**A**, **B**, **C**, **C**′, **I**, **I**′, **O**, **P**, **Q**, **R**).

 © The Author(s)
